# Synthesis, Reactivity, and Antibacterial Activity of *gem*-Difluoroalkene, Difluoromethyl, and Trifluoromethyl β-Lactams

**DOI:** 10.1021/acs.orglett.3c04094

**Published:** 2024-01-16

**Authors:** Monika Skibinska, Alicja Warowicka, Henryk Koroniak, Tomasz Cytlak, Benoît Crousse

**Affiliations:** † Faculty of Biology, Adam Mickiewicz University, Uniwersytetu Poznańskiego 6, 61-614 Poznań, Poland; ‡ Faculty of Chemistry, Adam Mickiewicz University, Uniwersytetu Poznańskiego 8, 61-614 Poznań, Poland; § BioCIS UMR 8076 CNRS, Building Henri Moissan, Université Paris-Saclay, 17 avenue des sciences, 91400 Orsay, France; ∥ Centre for Advanced Technologies, Adam Mickiewicz University, Uniwersytetu Poznańskiego 10, 61-614 Poznań, Poland

## Abstract

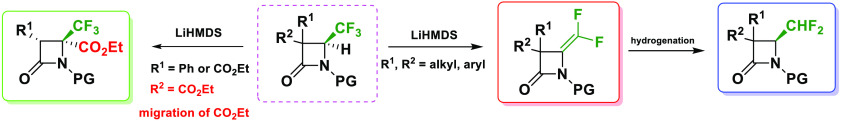

New *gem*-difluoroalkenes were synthesized by the dehydrofluorination of the corresponding 4-CF_3_-β-lactams. An unexpected rearrangement mechanism of the ester moiety dependent on a stabilizing negative charge was observed. Hydrogenation to 4-CHF_2_-β-lactams was successful from *gem*-difluoro-β-lactams.

*Gem*-difluoroalkenes are valuable fluorinated substructures and can be applied to prepare pharmaceuticals, agrochemicals, and materials.^1^ In addition, this moiety is characterized by unique features, such as its metabolic stability, the electronegative nature of the included fluorine atoms, and the chemical reactivity as electrophiles. The ability of *gem*-difluoroalkenes to mimic the carbonyl^2^ and amide groups^3^ has attracted attention for modifying biologically active compounds.^4^*gem*-Difluoroalkenes have served as irreversible electrophilic targets for inhibiting various enzymes.^5^*gem*-Difluoroalkenes, a versatile and readily available raw material class, have potential for preparing complex fluorinated compounds.^6^ Although the synthesis of the *gem*-difluoroalkenes is very well described,^7^ the presence of these motifs in β-lactams is not reported in the literature. Indeed, β-lactams (2-azetidinones) are of major interest for their biological properties, such as antibiotics^8^ and enzyme inhibitors,^9^ and their utility as intermediates in organic chemistry. Moreover, the combination of β-lactams and the difluoromethylene group could lead to an unprecedented new series of fluorinated molecules as potential biologically active compounds.^10^ These *gem*-difluoroalkene β-lactams serve also as a bioisostere of azetidine-2,4-diones (4-oxo-β-lactams), which are already known as a bioactive species, e.g., selective inhibitors of human neutrophil and human leukocyte elastase (Scheme 1).^11^

**Scheme 1:**
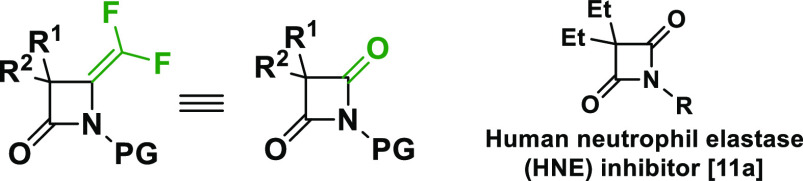
Geometry Comparison of *gem*-Difluoroalkene to Carbonyl Group

We previously reported the stereoselective synthesis of 3-mono and 3-disubstituted 4-CF_3_-β-lactams.^12^ Following this work, we planned to further study the deprotonation in the α position of the CF_3_ group of C-3 disubstituted β-lactams. Surprisingly, there is no precedent at the dehydrofluorination of CF_3_ into *gem*-difluoroalkene in lactams. We expect the formation of the *gem*-difluoroalkene without ring opening of the β-lactams, as observed in other families.^13^ Accordingly, we focused on access to *gem*-difluoroalkene β-lactams to study their reactivity and to examine their antibacterial activity (Scheme 2).

**Scheme 2:**
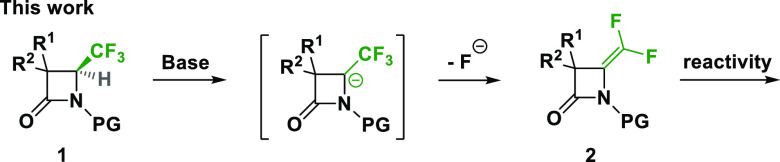
Dehydrofluorination of CF_3_-β-Lactams **1**

The dehydrofluorination reaction was tested in the presence of TEA (4 equiv), *t*-BuOK (2 equiv), *n*-BuLi (2–5 equiv), and LiHMDS (2 equiv) in a different time at room temperature, at reflux or −78 °C (Table 1, entries 1–4). The reactions were monitored by TLC and ^19^F NMR of the crude reaction. However, in any case, no traces of desired product **2a** were observed. Adding a distinct excess of LiHMDS (3–6 equiv) at −78 °C resulted in the formation of product traces (Table 1, entry 5). When we lowered the temperature to −10 °C by adding 2 equiv of LiHMDS, no reaction occurred (entry 6; Figure 1, spectrum B, SI). However, the addition of 3 equiv of LiHMDS under the same conditions afforded the corresponding *gem*-difluoroalkene **2a** without even a trace of ring-opening product but still with unreacted substrate **1a** (entry 7; Figure 1, spectrum C, SI). The addition of 4–5 equiv of LiHMDS led to the complete transformation of substrate **1a** into the corresponding *gem*-difluoroalkene **2a** (entry 8; Figure 1, spectra D, SI). The stable compound could be isolated after purification on silica gel in 66% yield. In another attempt, adding 6 equiv of LiHMDS induced formation side products (entry 9; Figure 1, spectra E, SI). Application of 10 equiv of LiHMDS (entry 10) gave the total decomposition of starting lactam **1a**.

**Table 1:**
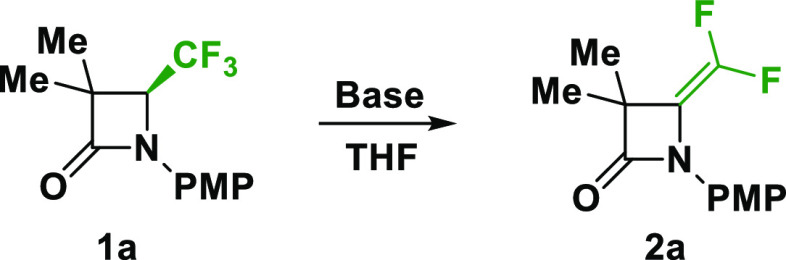
Various Conditions to Obtain *gem*-Difluoroalkene **2a**

Entry	Base	*T*°C	Time	2a, yield%
1	Et_3_N, 4 equiv	rt to reflux	2 h	n.r.^a^
2	*t*-BuOK, 2 equiv	–55 °C to rt	2 h	n.r
3	*n*-BuLi, 2–5 equiv	–78 °C to rt	2 h	n.r.
4	LiHMDS, 2 equiv	–78 °C to rt	45 min	n.r.
5	LiHMDS, 3–6 equiv	–78 °C to rt	45 min	traces
6	LiHMDS, 2 equiv	–10 °C to rt	45 min	n.r.
7	LiHMDS, 3 equiv	–10 °C to rt	45 min	32%
8	LiHMDS, 4–5 equiv	–10 °C to rt	45 min	66%
9	LiHMDS, 6 equiv	–10 °C to rt	45 min	44%^b^
10	LiHMDS, 10 equiv	–10 °C to rt	45 min	decomposition

a No reaction.

b Formation of side products.

With these results, the optimized conditions were explored for other 3-disubstituted 4-CF_3_-β-lactams, formerly prepared in our laboratory (**1b, c, e, f, j, k, l**)^12a^ or synthesized particularly to enlarge the family of compounds (**1d, g, h, i, m**, SI), according to our previous procedure. These substituted β-lactams have aryl and alkyl functions such as methyl, ethyl, benzyl, and allyl groups. The corresponding *gem*-difluoroalkenes **2b**–**2m** were obtained in moderate to good yields (Scheme 3). Surprisingly, in the case of 3,3-dicarboxylate-4-CF_3_-β-lactam **1n**, the expected 3,3-dicarboxylate-4-difluoromethylene product **2n** was not observed, using the previous conditions (4 equiv of LiHMDS in THF at −10 °C to rt). In the crude mixture, only one diastereoisomer of product **3** was identified as still possessing the CF_3_ group and was isolated in 57% yield (Scheme 4). The structure and stereochemistry of **3** were determined by MS and NMR (see Figure 2, SI). During the reaction, after deprotonation of α of the CF_3_ group, an ester group migrated from position 3 to position 4. This reaction progressed with excellent stereoselectivity. The analysis suggested that the H-3 and CF_3_ group are *cis* configured to each other.

**Scheme 3:**
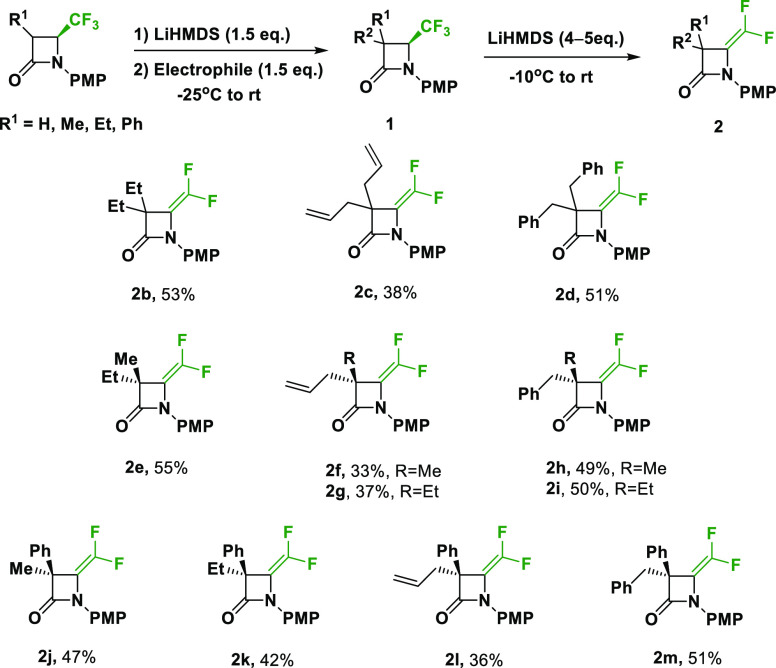
Formation of *gem*-Difluoroalkenes **2**

**Scheme 4:**
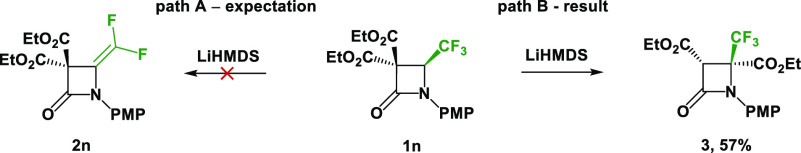
Reaction of 3,3-Dicarboxylate-4-CF_3_-β-lactam **1n**

To further investigate the unexpected migration of the ester group, reactions were performed on 3-carboxylate-3-Me-4-CF_3_-β-lactam **1o** and 3-carboxylate-3-Ph-4-CF_3_-β-lactam **1p**. In the case of 3-carboxylate-3-Me-4-CF_3_-β-lactam **1o**, ethyl 3-carboxylate-3-Me-4-difluoromethylene-β-lactam **2o** was observed as the primary product with 47% yield with only traces of rearranged product **4** (Scheme 5). On the other hand, from the 3-carboxylate-3-Ph-4-CF_3_-β-lactam **1p**, the migration of the ester group occurred to give lactam **5** as the major product, with only traces of the corresponding *gem*-difluoroalkene **2p** (Scheme 5). The structure and relative configuration of the obtained product **5** were determined based on the MS and NMR analysis (see SI).

**Scheme 5:**
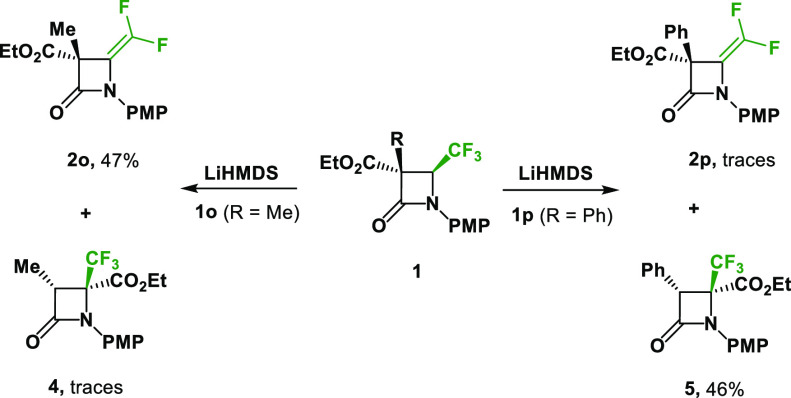
Different Reactivity of 3,3-Dicarboxylate-4-CF_3_-β-lactams **1o**, **1p**

The difference in reactivity between 3,3-dicarboxylate-4-CF_3_-β-lactam **1n**, 3-carboxylate-3-Ph-4-CF_3_-β-lactam **1p**, and 3-carboxylate-3-Me-4-CF_3_-β-lactam **1o** can probably be explained by the delocalization or lack thereof of the negative charge in the core of the lactam according to the group at the C-4 position (Scheme 6). After deprotonation of α to CF_3_, the anion attacks the ester group to give intermediate **A**. The migration of the ester is driven by generation of the more stable anion with the aryl or ester group and the carbonyl. Thus, compounds **3** and **5** are major products. On the other hand, in the case of the methyl group, the enolate ion is significantly destabilized and leads preferentially to the *gem*-difluoroalkene compound **2o**.

**Scheme 6:**
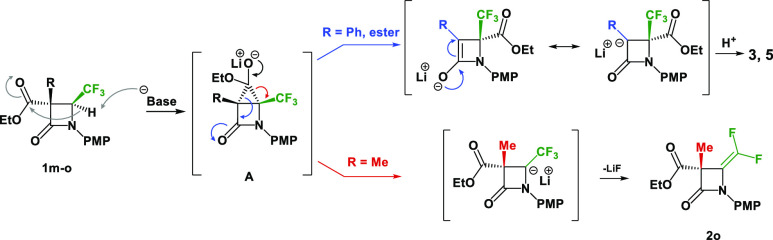
Stabilization of Formed Enolate Ion Facilitating of Rearrangement Products **3** and **5**

During the study, the reaction was carried out on unsubstituted and monosubstituted lactams, 4-CF_3_-β-lactam **6** and 3-methyl-4-CF_3_-β-lactam **7** in the presence of 3 equiv of LiHMDS. Only the starting lactams **6** and **7** are present in the reaction mixture (Scheme 7). By increasing the amount of LiHMDS up to 6–10 equiv, the reaction led to the decomposition of **6** and **7** without traces of *gem*-difluoroalkene **2**. However, from the *cis*/*trans* mixture 3-Me-4-CF_3_-β-lactam **7**, only the *trans* isomer was observed in the crude mixture. C-3 isomerization/racemization occurred for the thermodynamically preferred diastereoisomer 3-Me-4-CF_3_-β-lactam **7**. The same results were observed from the mixture of *cis*/*trans*-3-alkyl/aryl-4-CF_3_-β-lactams, leading to 3-alkyl/aryl-4-CF_3_-β-lactams with the *trans* configuration.

**Scheme 7:**
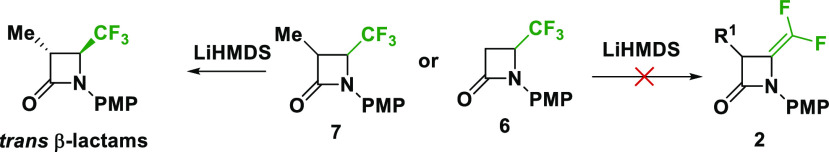
Lack of Reactivity of C-3 Mono- and Unsubstituted 4-CF_3_-β-Lactams **6** and **7** toward Corresponding *gem-*Difluoroalkenes **2**

It is important to note that access to β-lactams possessing the CF_2_H group is rare in the literature.^14^ Moreover, the hydrogenation of *gem*-difluoroalkene moiety can get access to the difluoromethyl group, which is a lipophilic hydrogen bond donor because of the high polarization of the C–H bond, allowing it to act as a “lipophilic bioisostere” of alcohols and thiols.^15^ So, hydrogenation of the *gem*-difluoroalkene group seemed like an excellent alternative to provide a novel family of CF_2_H β-lactams. Accordingly, reaction of 3,3-dimethyl- and 3-carboxylate-4-difluoromethylene-β-lactam **2a** and **2o** was conducted under a hydrogen atmosphere in the presence of a palladium catalyst on activated carbon.^16^ Successfully, the reduced products, 4-difluoromethyl-β-lactams **8** and **9**, were obtained in excellent yields, 95% and 99%, respectively (Scheme 8). The reaction progressed in either ethanol or ethyl acetate. Nevertheless, the reaction in ethyl acetate was slower.

**Scheme 8:**
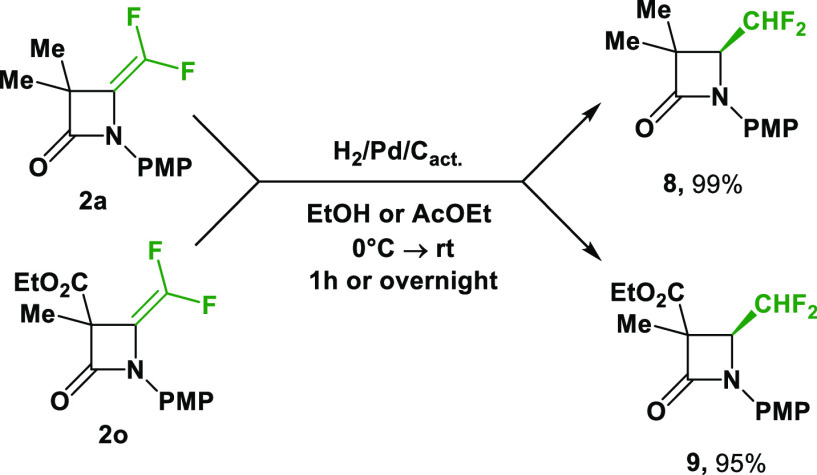
Hydrogenation of *gem-*Difluoroalkenes **2a** and **2o**

Having prepared new families of *gem*-difluoroalkene and difluoromethyl β-lactams, a screening was performed for their antibacterial activity against *Gram*-positive bacteria: *Staphylococcus aureus* (*S. aureus*, ATCC 25923), methicillin–resistant *Staphylococcus aureus* (MRSA, ATCC 43300), *Bacillus subtilis* and *Gram*-negative bacterial strain: *Escherichia coli* (*E. coli*, ATCC 25922). As preliminary results, disc diffusion assays evaluated the antibacterial activity. The zone of growth inhibition with clear boundaries was noticed for all tested bacteria: *E. coli*, *S. aureus*, *MRSA*, and *Bacillus sub*. Compounds **1a**, **2a**, **8**, and **9** showed the highest activity against *gram*-negative *E. coli* and *Bacillus subtilis* (the size of the inhibition zone was 4–5 mm) and compounds **2a**, **2l**, and **2o** against *gram*-positive *MRSA* and *S. aureus*. The study showed that not only *gem*-difluoroalkene β-lactams could inhibit the growth of bacteria. Indeed, we observed the highest activity against *E. coli* for compound **1a** (5 mm), bearing the 4-CF_3_ substituent. Slightly less activity (4 mm) was also detected for compounds **8** and **9**, bearing the 4-CF_2_H substituent. Importantly, nonfluorinated β-lactam **10** did not show any activity, which confirms the effect of fluorine on the appearance of activity. The results obtained from the disc diffusion assay for other compounds are presented in the Supporting Information (Table 1 and Figures 3–10).

Due to the results of the diffusion assay, selected compounds were evaluated as the bacterial β-lactamase inhibitors. The potential inhibitory activity of the compounds was determined by a β-lactamase inhibitor colorimetric assay. The results revealed that compounds **1a**, **2a**, and **2d** exhibited β-lactamase inhibition potential. The highest % of inhibition showed *gem*-difluoroalkene β-lactam **2a** (inhibition of β-lactamase in 46.4%). Similarly, compound **1a** inhibited β-lactamase in 45.2%. Importantly, in the presence of compound **2d** activity of β-lactamase was decreased to 35.7% (SI Figures 11–15). This suggests that the mechanism of inhibition arises from β-lactamase inhibition. These results demonstrated that selected compounds possess the inhibitory activity of β-lactamase and may be developed as new β-lactam antibiotics. All biological data are reported in the Supporting Information.

In conclusion, we have developed an efficient method for the preparation of *gem*-difluoroalkene-β-lactams due to the dehydrofluorination of 4-CF_3_-β-lactams. During our study, an unprecedented migration of an ester group was observed from position 3 to position 4. From *gem*-difluoroalkene lactams, hydrogenation afforded 4-CHF_2_-β-lactams in excellent yields. Then, the antibacterial activity of 4-difluoroalkene-β-lactams and 4-CHF_2_-β-lactams was investigated. Diffusion disc and β-lactamase inhibitor screening assay results demonstrated that some fluorinated β-lactams exhibit antibacterial activity due to β-lactamase inhibition. Other biological targets and biological mechanisms are currently being studied to confirm the beneficial impact of these fluorinated scaffolds.

## Data Availability

The data underlying this study are available in the published article and its Supporting Information.

## Supporting Information Available

Detailed experimental procedures; biological tests, characterization data; and copies of ^1^H, ^13^C, ^19^F NMR and ^1^H–^13^C HSQC, ^1^H–^19^F HOESY spectra (PDF)
