# Supplementary material for: Synthesis, Reactivity, and Antibacterial Activity of gem-Difluoroalkene, Difluoromethyl, and Trifluoromethyl β-Lactams
Source: Org Lett. 2024 Jan 16;26(3):692–6. doi: 10.1021/acs.orglett.3c04094 (PMC10825868; doi:10.1021/acs.orglett.3c04094)

Supporting Information

Synthesis, reactivity and antibacterial activity of *gem*-difluoroalkene, difluoromethyl and trifluoromethyl β-lactams

Monika Skibinska[1], Alicja Warowicka[2], Henryk Koroniak[1], Tomasz Cytlak*[1,3], Benoît Crousse*[4]

(1) Faculty of Chemistry, Adam Mickiewicz University, Uniwersytetu Poznańskiego 8, 61-614 Poznań, Poland,
(2) Faculty of Biology, Adam Mickiewicz University, Uniwersytetu Poznańskiego 6, 61-614 Poznań, Poland,
(3) Centre for Advanced Technologies, Adam Mickiewicz University, Uniwersytetu Poznańskiego 10, 61-614 Poznań, Poland
(4) BioCIS UMR 8076 CNRS, Univ. Paris-Saclay, Building Henri Moissan, 17 rue des sciences, 91400 Orsay, France

Corresponding authors: cytlak@amu.edu.pl and benoit.crousse@universite-paris-saclay.fr

Table of Contents

1. **Figure 1……………………………………………………………………………………….S2**
2. **Figure 2……………………………………………………………………………………….S2**
3. **Biological evaluation – antimicrobial activity…………………………………………...S3**
4. **Materials and methods……………………………………………………………………….S9**
5. **References…………………………………………………………………………………….S22**
6. **^{1}H, ^{13}C, ^{19}F and selected 2D and selective 1D Spectra of β-lactams……………….…………...S23**

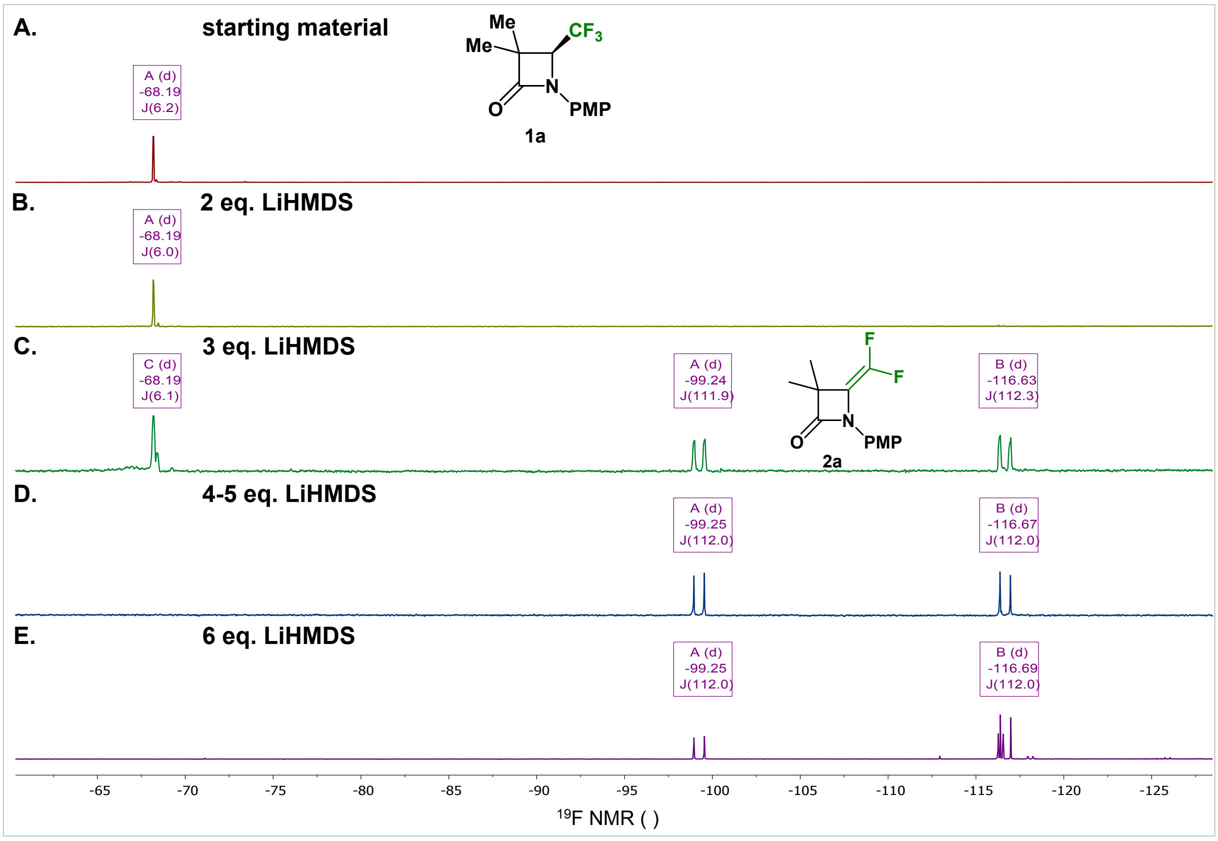

Figure 1. ^{19}F NMR monitoring the dehydrofluorination of **1a** while increasing the equivalents of LiHMDS added to the reaction mixture.

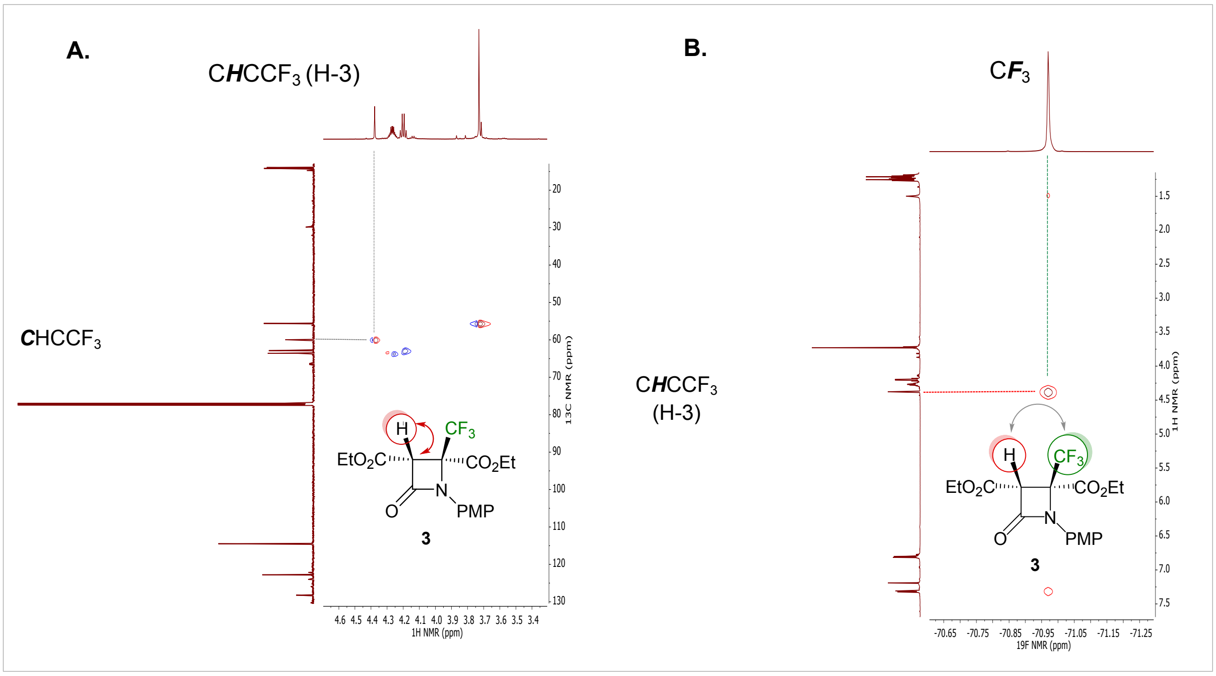

Figure 2. **A** –2D ^{1}H–^{13}C HSQC NMR spectrum and **B** – 2D ^{1}H–^{19}F HOESY NMR spectrum of compound **3**.

Biological evaluation - antimicrobial activity

Gem-difluoroalkenes are considered as a biological important motif that could be bioisostere for carbonyl group.[1] This fact together with specific characteristics of the fluorine atom, e.g. high lipophilicity, good absorbability, and strong electron-withdrawing ability can improve the properties of target bioactive molecules.[2] In case of artemisinin, replacement of the carbonyl group by a *gem*-difluoroalkene can improve its antimalarial activity.[2a,b] There are also examples that the introducing *gem*-difluoroalkene moiety can reverse the regioselectivity of enzyme-catalyzed hydridic reduction.[2b] In general, *gem*-difluoroalkenes were mainly examined as irreversible electrophilic warheads towards various enzymes inhibition.[3] Moreover, the hydrogenation of *gem*-difluoroalkene moiety can get access to difluoromethyl group (CHF_2) which has been shown to be lipophilic hydrogen bond donor because of the high polarization of the C–H bond, allowing it to act as a „liphophilic bioisostere" of alcohols and thiols.[4] Despite the potential of *gem*-difluoromethyl groups to mimic biologically important structure motifs, a survey of fluorine containing pharmaceuticals reveals mainly trifluoromethyl and monofluoromethyl compounds and only few examples of difluoromethyl moieties.[5]

Due to these facts, newly synthesized 4-difluoromethylene-*β*-lactams **2** were screened for their antibacterial activity against Gram positive bacteria: *Staphylococcus aureus* (*S. aureus*, ATCC 25923), methicillin – resistant *Staphylococcus aureus* (MRSA*,* ATCC 43300), *Bacillus subtilis* and Gram negative bacterial strain: *Escherichia coli* (*E. coli*, ATCC 25922). The antibacterial activity was evaluated by the disc diffusion assay and next selected compounds were tested towards the *β*-lactamase inhibition activity.

The antibacterial activity of 4-difluoromethylene-*β*-lactams **2** was first evaluated by the disc diffusion assay together with simultaneous comparison to activity of 4-trifluoromethyl-*β*-lactam **1a** and 4-difluoromethyl-*β*-lactams **8, 9**. The zone of growth inhibition with clear boundaries was noticed for all tested bacteria: *E. coli, S. aureus, MRSA, Baccilus sub*. The results from the agar disc diffusion assays showed that tested compounds have different effect against various bacteria strains. Interestingly, the smaller zone of growth inhibition was detected for *S. aureus* and *MRSA*, where the highest activity was detected for compounds **2a**, **2l**, **2o**, **8** and **9** (the size of the inhibition zone was 3 mm). In the case of *Baccilus sub.* for almost all tested compounds (except **2i**) the size of the inhibition zone was found in a very similar range (4-5 mm). Against gram-negative *E. coli*, compounds **1a**, **2a**, **8** and **9** showed the highest activity (the size of the inhibition zone was 4-5 mm). The results obtained from the disc diffusion assay are presented in Table 1. Our study suggests that not only *gem*-difluoroalkene *β*-lactams could inhibit the growth of bacteria. We observed the highest activity against *E. coli* for compound **1a** (5 mm), bearing 4-CF_3 substituent, and slightly less activity (4 mm)

was also detected for compounds **8** and **9**, bearing 4-CF_2H substituent. Similarly, for *S. aureus*, *MRSA*, except the activity of certain *gem*-difluoroalkenes, we noticed that compounds **8** and **9** (4-CF_2H) were active on the same level of growth inhibition. Additionally, we synthesized and tested one example of non-fluorinated reference compound, 4-propyl-*β*-lactam **10**, which did not show any activity (Table 1 and Fig. 3-9). The reference antibiotics (trimethoprim, rifampicin, sulphamethoxonazole/trimethoprin) were used as positive control for diffusion assay (Fig. 10).

Table 1. Inhibition zone diameters (mm)

Compound	Antibacterial activity (zone diameters in mm)			
	S. aureus (ATCC 25923)	*MRSA* (ATCC 43300)	*E. coli* (ATCC 25922)	*Baccilus sub.*
**1a**	2	2	**5**	4
**2a**	**3**	**3**	**4**	5
**2b**	2	1	2	5
**2c**	2	1	3	5
**2d**	2	1	3	4
**2e**	2	1	3	5
**2f**	1	2	3	4
**2g**	1	2	2	3
**2h**	2	1	3	4
**2i**	2	2	2	2
**2j**	1	1	3	4
**2k**	1	2	3	4
**2l**	**3**	**3**	3	5
**2m**	2	1	3	5
**2o**	**3**	**3**	3	5
**8**	**3**	2	**4**	5
**9**	2	**3**	**4**	5
**10**	0	0	0	0

The non-fluorinated reference, 4-propyl-*β*-lactam **10** was synthesized from corresponding imine, that was prepared according to literature method [6], from butyrate aldehyde. Then we obtained *β*-lactam **10**, using the same procedure as for the synthesis of 4-CF_3-*β*-lactams [7].

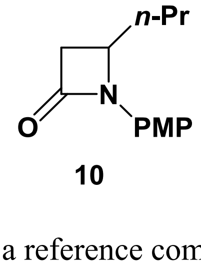

10

Figure 3. Non-fluorinated *β*-lactam **10** as a reference compound for diffusion discs assay antibacterial evaluation.

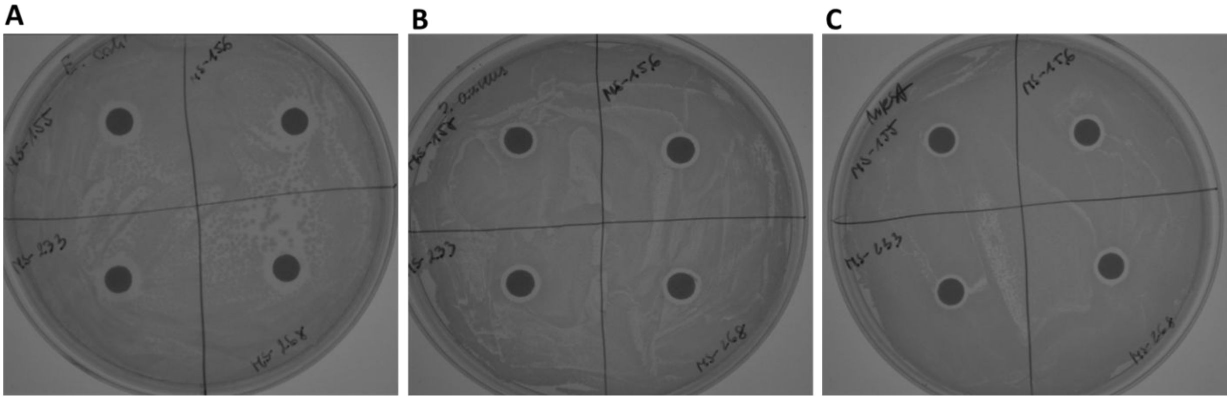

Figure 4. Antimicrobial activities of studied compounds (**1a**, **2a**, **8**, **9**) evaluated by the disk diffusion assay: A. Inhibition zones against *E. coli* (ATCC 25922), B. Inhibition zones against *S. aureus* (ATCC 25923), C. Inhibition zones against MRSA (ATCC 43300).

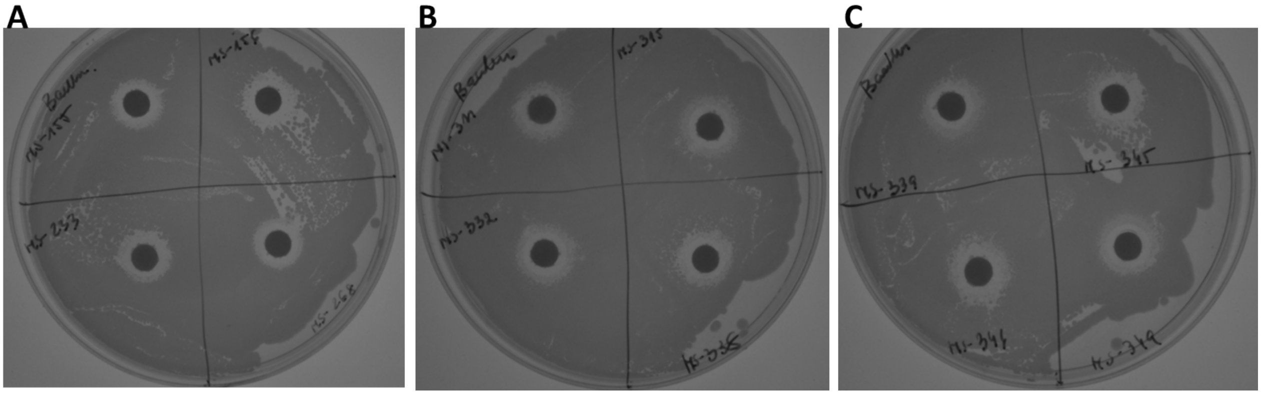

Figure 5. Antimicrobial activity of selected compounds (**1a**, **2a**, **8**, **9**) against *Bacillus sub.*

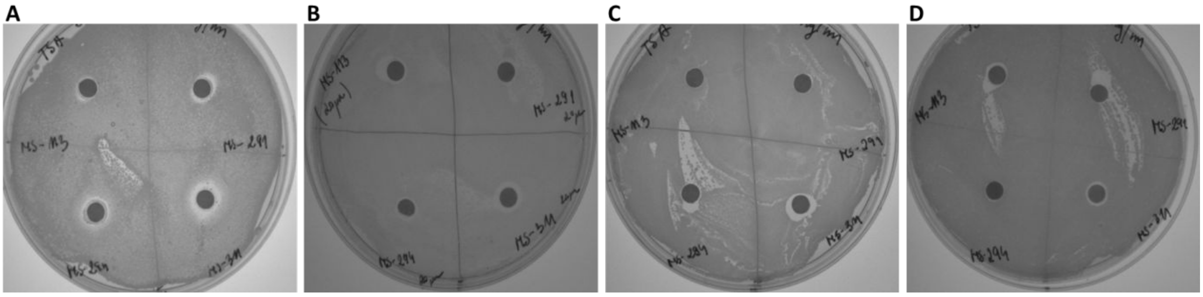

Figure 6. Antimicrobial activities of selected compounds (**2g**, **2h**, **2i**, **2o**) tested by the disc diffusion assay. Inhibition zones against: A. *Bacillus sub*.; B. *E. coli* (ATCC 25922); C. *S. aureus* (ATCC 25923); D. MRSA (ATCC 43300)

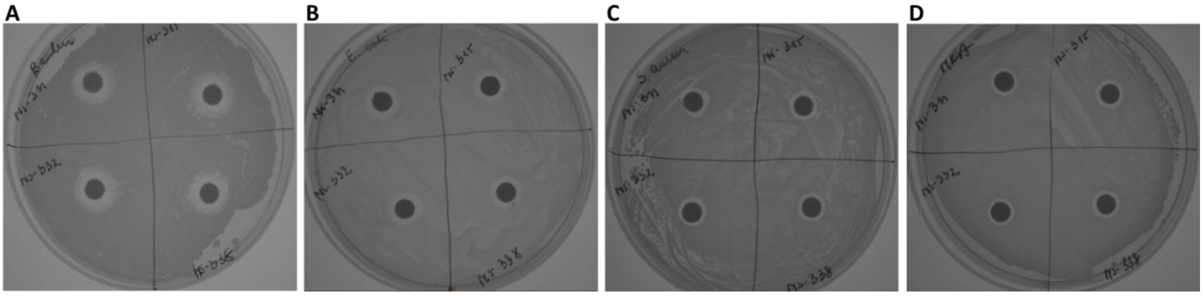

Figure 7. Antimicrobial activities of selected compounds (**2i**, **2j**, **2k**, **2m**) tested by the disc diffusion assay. Inhibition zones against: A. *Bacillus sub*.; B. *E. coli* (ATCC 25922); C. *S. aureus* (ATCC 25923); D. MRSA (ATCC 43300)

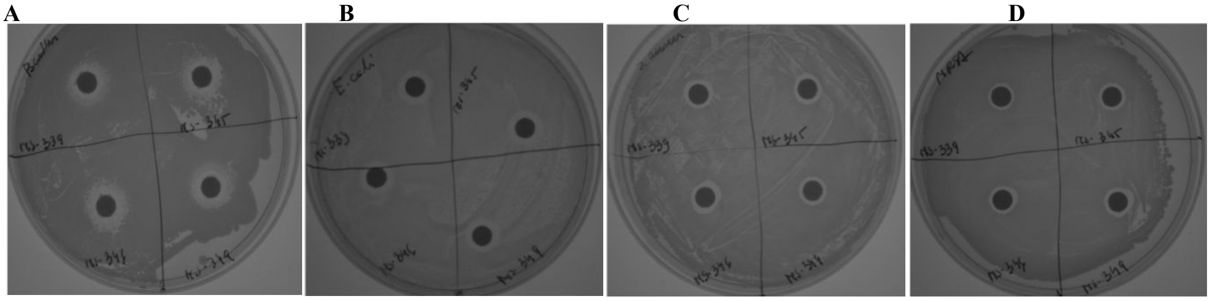

Figure 8. Antimicrobial activities of selected compounds (**2c**, **2d**, **2e**, **2f**) tested by the disc diffusion assay. Inhibition zones against: A. *Bacillus sub*.; B. *E. coli* (ATCC 25922); C. *S. aureus* (ATCC 25923); D. MRSA (ATCC 43300)

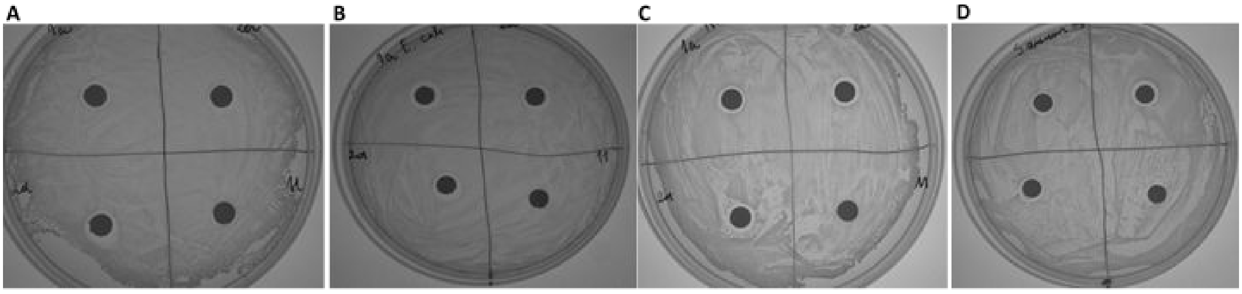

Figure 9. Antimicrobial activities of selected compounds (**1a, 2a, 2d, 10**) tested by the disc diffusion assay. Inhibition zone against: A. *Bacillus sub*.; B. *E. coli* (ATCC 25922); C. *S. aureus* (ATCC 25923); D. *MRSA* (ATCC 43300). Compound **10** (bottom-right) did not show any activity

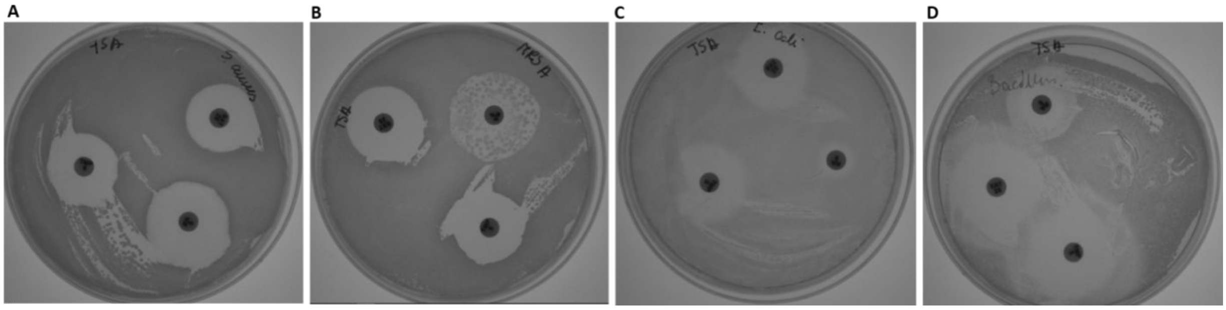

Figure 10. Antimicrobial activity of antibiotics: TMP (Trimethoprin), RA (Rifampicin), SXT (Sulphamethoxonazole/Trimethoprin) against A. *S. aureus* (ATCC 25923), B. MRSA (ATCC 43300), C. *E. coli* (ATCC 25922), D. *Bacillus sub.*

In order to evaluate if our selected compounds **1a, 2a, 2d** can inhibit bacterial β-lactamases, colorimetric β-lactamase Inhibitor Screening Kit assay was applied. In this convenient assay the activity of β-lactamase was measured spectrophotometrically. The potential inhibitory activity of compounds was determined by colorimetric assay. The amount of produced color is directly proportional to the beta-lactamase (β-lactamase, βLac) enzyme activity (Fig. 11). For inhibition efficiency evaluation the % of relative inhibition was calculated. The absorbance (A_{490}) was plotted versus time for each sample and the slope of the plot (A_{490}/min.) was expressed (Fig. 12-15). The % of relative inhibition was determined as follows:

% Relative inhibition = $(slope_{EC} - slope_{S})/slope_{EC} \times 100\%$

where $slope_{EC}$ was the slope of enzyme control (without inhibitor), $slope_{S}$ was the slope of studied compound (potential inhibitor). Slope = ΔABS/minute.

The results revealed that compounds **1a, 2a, 2d** exhibited β-lactamase inhibition potential. The higher % of inhibition showed *gem*-difluoroalkene β-lactam **2a** (inhibition of β-lactamase in 46.4 %). Similarly, compound **1a** inhibited β-lactamase in 45.2 %. Importantly, in the presence of compound **2d** activity of β-lactamase was decreased to 35.7 %. These results demonstrated that selected compounds possess inhibitory activity of β-lactamase and may be developed as the new β-lactam antibiotics.

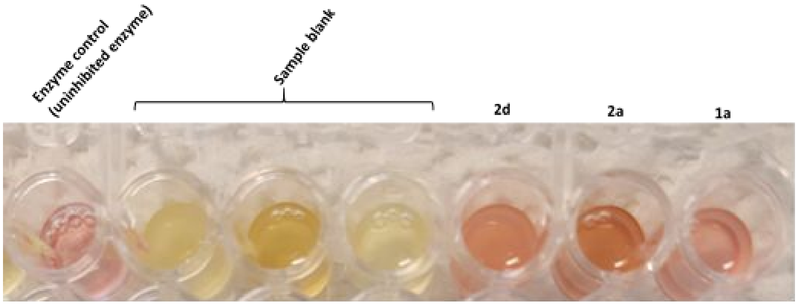

Figure 11. The visual effect of β-lactamase activity (hydrolysis a chromogen nitrocefin, producing a coloured product). Pink colour in well indicates hydrolysis of nitrocefin, a substrat for β-lactamase. Due to degradation (hydrolysis) nitrocefin changes colour from yellow to light pink.

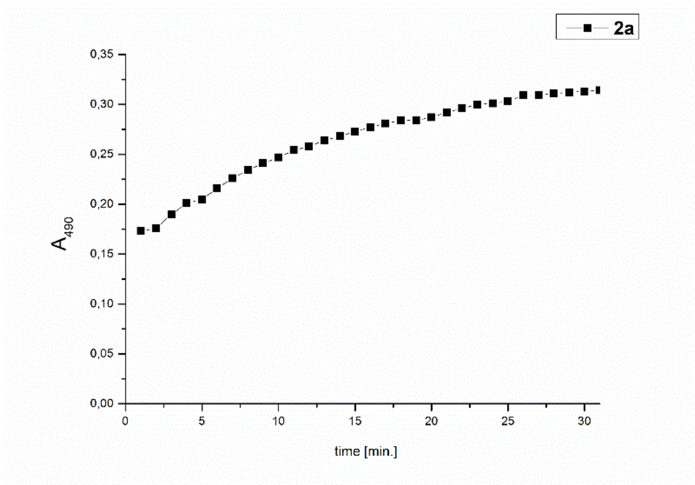

Figure 12. β-lactamase activity in the presence of compound **2a.** Absorbance at 490 nm (A_{490}) was measured every minute, for 30 minutes using a microplate spectofotometer.

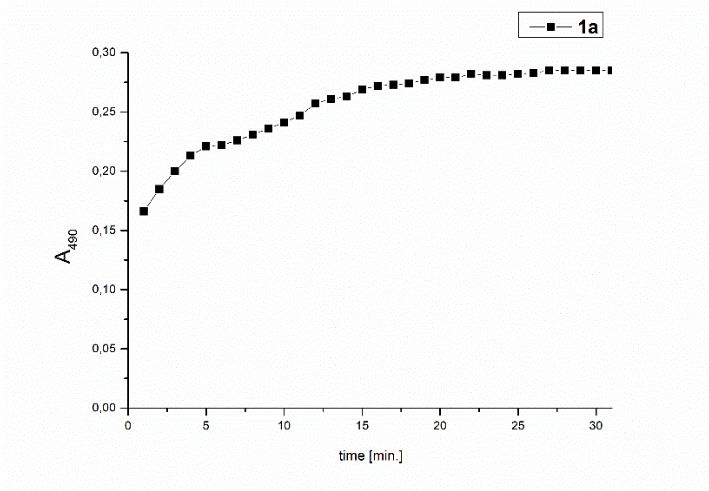

Figure 13. β-lactamase activity in the presence of compound **1a.** Absorbance at 490 nm (A_{490}) was measured every minute, for 30 minutes using a microplate spectofotometer.

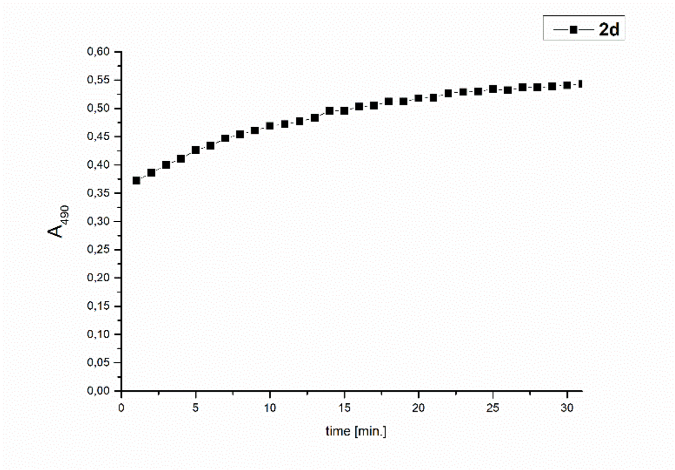

Figure 14. β-lactamase activity in the presence of compound **2d.** Absorbance at 490 nm (A_{490}) was measured every minute, for 30 minutes using a microplate spectofotometer.

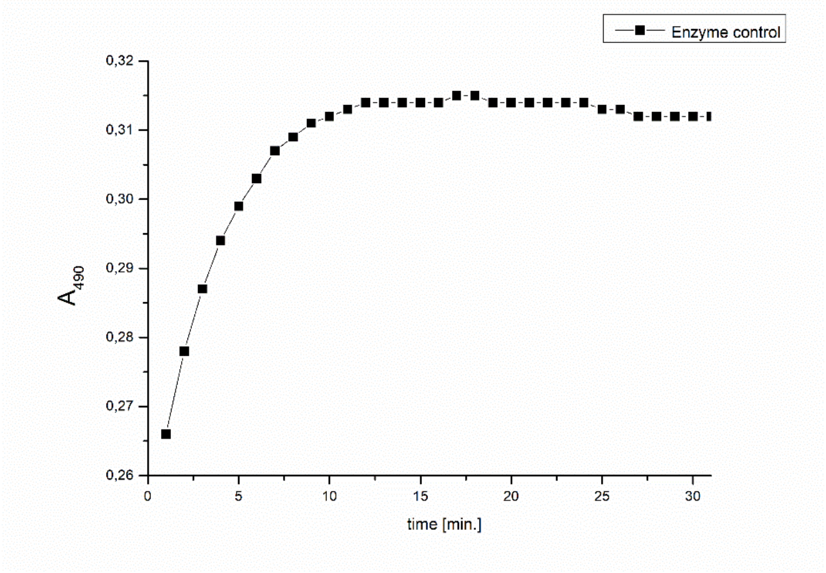

Figure 15. β-lactamase activity in the absence of potential inhibitor (enzyme control)**.** Absorbance at 490 nm (A_{490}) was measured every minute, for 30 minutes using a microplate spectofotometer.

Materials and Methods

Experimental section

Antibacterial studies

To determine the antibacterial properties of synthetized compounds, bacterial strains *Staphylococcus aureus* (*S. aureus*, ATCC 25923), methicillin – resistant *Staphylococcus aureus* (MRSA*,* ATCC 43300), *Escherichia coli* (*E. coli*, ATCC 25922), Bacillus sub. were subjected to biological tests. All bacteria species bacterial strains were purchased from the Polish Collection of Microorganisms

(Wrocław, Poland). Evaluation of bacteriostatic activities of compounds were carried out by the disc diffusion method and by the *β*-lactamase inhibition screening assay.

Disc diffusion assay

To evaluate the antibacterial activity of compounds, the disc diffusion assay was used. Compounds were dissolved in chloroform. Sterile paper discs (BioMaxima) were impregnated with compound, and the loaded filter paper discs were placed at precise locations on the prepared agar plates (TSA). The TSA plates containing filter paper discs loaded with various compounds were incubated at 37 °C for 24 h. After incubation, the inhibition zones were measured in millimeters (mean ± SD, n = 2). The results are presented in Table 2. The diameters of the zones were compared with those of standard antibiotics. Antibiotic discs (BioMaxima) used as a positive controls: TMP (Trimethoprin), RA (Rifampicin) and SXT (Sulphamethoxonazole/Trimethoprin)

Evaluation of β-lactamase inhibition

In order to evaluate if our designed compounds **1a, 2a, 2d** can inhibit bacterial β-lactamases, colorimetric *β*-lactamase Inhibitor Screening Kit (Merck, cat. no. MAK222) assay was applied following described protocol. In this convenient assay the activity of β-lactamase was measured spectrophotometrically. Briefly, equal amounts of each studied compound (125 µg in 20 µl of DMSO) were added separately to the wells of flat-bottom clear microplate for colorimetric assay (Greiner Bio-One). Then, 50 µl of Inhibition Reaction Mix (48 µl of β-lactamase assay buffer and 2 µl of β-lactamase) was added to each well. After incubation time (10 min., 25°C, in dark) 30 µl of Enzymatic Reaction Mix (1 µl of nitrocefin, a chromogenic substrate for β-lactamase enzyme and 29 µl of β-lactamase assay buffer) was added and mixed well. The final reaction volume in each well was 100 µl. Hydrolysis of nitrocefin by β-lactamase produces coloured product (light pink). Immediately after the addition of nitrocefin, the absorbance value was determined/recorded at 490 nm using a microplate reading spectrophotometer in kinetic mode (Synergy H1 Hybrid Reader, BioTek Instruments, Inc.). The measurement was recorded every minute for 30 minutes at room temperature. The amount of produced color is directly proportional to the beta-lactamase (β-lactamase, βLac) enzyme activity. The reaction plate was photographed. For the inhibition assay, samples blank (omitting of β-lactamase in Inhibition Reaction Mix), enzyme control (uninhibited β-lactamase, β-lactamase assay buffer in place of sample inhibitor) and inhibitor control (clavulanic acid, an inhibitor of β-lactamase, in β-lactamase assay buffer) were added. All reactions were performed in triplicate.

General Methods: ^{1}H NMR, ^{13}C NMR, ^{19}F NMR and ^{31}P NMR spectra were performed on Bruker ASCEND 600 (600 MHz) Bruker ASCEND 400 (400 MHz) Bruker ASCEND 300 (300 MHz)

spectrometers. Structural assignments were made with additional information from gCOSY, gHSQC, and gHMBC experiments. All 2D and 1D selective NMR spectra were recorded on Bruker ASCEND 600 (600 MHz) spectrometer. Chemical shifts of ^{1}H NMR were expressed in parts per million downfield from tetramethylsilane (TMS) as an internal standard (δ = 0) in $CDCl_3$. Chemical shifts of ^{13}C NMR were expressed in parts per million downfield and upfield from $CDCl_3$ as an internal standard (δ = 77.0). Chemical shifts of ^{19}F NMR were expressed in parts per million upfield from $CFCl_3$ as an internal standard (δ = 0) in $CDCl_3$. All d.r. were evaluated on the basis of ^{19}F NMR reaction mixture. High-resolution mass spectra were recorded by electron spray (MS-ESI) techniques using QToF Impact HD Bruker spectrometer. Reagent grade chemicals were used. THF was dried by refluxing with sodium metal-benzophenone (THF) and distilled under argon atmosphere. All moisture sensitive reactions were carried out under argon atmosphere using ovendried glassware. Reaction temperatures below 0°C were performed using a cooling bath (liquid N_2/*n*-hexane or liquid N_2/*i*-PrOH). TLC was performed on Merck Kieselgel 60-F254 with EtOAc/*n*-hexane and MeOH/$CHCl_3$ as developing systems, and products were detected by inspection under UV light (254 nm) and with a solution of potassium permanganate. Merck Kieselgel 60 (0.063-0.200 μm), Merck Kieselgel 60 (0.040-0.063 μm), Merck Kieselgel 60 (0.015-0.004 μm), were used for column chromatography.

4-CF_3 *β*-lactams **1a-c**, **1e-f**, **1j-l**, **1n-p, 6, 7**, which we used as a starting materials in dehydrofluorination reaction, were prepared previously in our laboratory [7]. For the synthesis of other starting 4-CF_3 *β*-lactams **1d**, **g**, **h**, **i**, **m** we applied the same procedure.

General procedure for the preparation of 4-CF_3 *β*-lactams 1d, g, h, i, m

In a round-bottom flask, dry THF (2 mL) was cooled to −25°C, and then LiHMDS (1.5 or 2.5 or 3 eq., 1 M in THF) was added dropwise under an argon atmosphere. Next, the solution of the corresponding β-lactam (1 eq.) in dry THF (1 mL) was added. The suspension was stirred at the same temperature for 30 min. and then the electrophile (1.2 or 2 eq.) was added dropwise. Then, the reaction mixture was stirred at the same temperature for 2 h and left overnight at room temperature. Afterwards, the reaction mixture was cooled to 0°C and carefully quenched by dropwise addition of saturated NH_4Cl aq. (1 mL), and extracted with Et_2O (2×5 mL). The organic layers were washed with brine, dried over anhydrous $MgSO_4$ or Na_2SO_4, filtrated, and concentrated under reduced pressure. The crude product(s) was/were purified using column chromatography (*n*-hexane/ethyl acetate or cyclohexane/ethyl acetate).

Racemic mixture of (S)-3,3-dibenzyl-1-(4-methoxyphenyl)-4-(trifluoromethyl) azetidin-2-one (1d)

The racemic mixture of **1d** was obtained as a pale yellow oil (159 mg, 62%). To the reaction *N*-PMP 4-CF_3-*β*-lactam (148 mg, 0.60 mmol, 1 eq.), benzyl bromide (0.13 mL, 1.14 mmol, 1.9 eq.) and LiHMDS (1.25 mL, 1.25 mmol, 2.5 eq.) were used, following the general procedure. The crude product was purified *via* column chromatography, using mixture of *n*-hexane/ethyl acetate 90:10, v/v.

^{1}H NMR (400 MHz, $CDCl_3$): δ = 7.42 (d, *J* = 7.3 Hz, 2H, Ph), 7.34 – 7.02 (m, 8H, Ph) 6.78 (d, *J* = 8.9 Hz, 2H, Ar), 6.66 (d, *J* = 9.0 Hz, 2H, Ar), 4.08 (q, *J* = 7.0 Hz, 1H, C*H*CF_3), 3.67 (s, 3H, OC*H*$_3$), 3.56 (d, *J* = 14.1 Hz, 1H, C*H*HPh), 3.02 – 2.88 (m, 2H, CH*H*Ph, C*H*HPh), 2.62 (d, *J* = 14.2 Hz, 1H, CH*H*Ph). ^{13}C NMR (101 MHz, $CDCl_3$): δ = 168.5 (s, N*C*=O), 157.6, 135.6, 135.4, 130.9, 130.2, 129.9, 128.8, 128.5 127.5, 127.2 (10 × s, Ar, Ph), 124.8 (q, *J* = 281.1 Hz, *C*F_3), 121.7, 114.3 (2 × s, Ar, Ph), 61.7 (s, *C*CHCF_3), 58.6 (q, *J* = 32.9 Hz, *C*HCF_3), 55.5 (s, O*C*H_3), 39.2 (s, *C*H_2Ph), 37.1 (s, *C*H_2Ph). ^{19}F NMR (376 MHz, $CDCl_3$): δ = −66.87 (d, *J* = 7.0 Hz). HRMS (ESI) *m/z:* $[M + Na]^+$ Calcd for $C_{25}H_{22}F_3NO_2Na^+$ 448.1500; Found 448.1496.

Racemic mixture of (3R,4S)-3-allyl-3-ethyl-1-(4-methoxyphenyl)-4-(trifluoromethyl)azetidin-2-one (1g)

The racemic mixture of **1g** was obtained as a pale yellow oil (96 mg, 55%). To the reaction the mixture of *cis/trans* *N*-PMP 3-Et-4-CF_3-*β*-lactam (152 mg, 0.56 mmol, 1 eq.), allyl bromide (58 µL, 0.66 mmol, 1.2 eq.) and LiHMDS (0.84 mL, 0.84 mmol, 1.5 eq.) were used, following the general procedure. The crude product was purified *via* column chromatography, using mixture of *n*-hexane/ethyl acetate 85:15, v/v.

^{1}H NMR (400 MHz, $CDCl_3$): δ = 7.35 – 7.29 (m, 2H, Ar), 6.91 – 6.85 (m, 2H, Ar), 5.76 (ddt, *J* = 17.1, 10.1, 7.3 Hz, 1H, *H*C=CH_2), 5.31 – 5.18 (m, 2H, HC=C*H*$_2$), 4.25 (q, *J* = 6.7 Hz, 1H, C*H*CF_3), 3.79 (s, 3H, OCH_3), 2.56 (d, *J* = 7.3 Hz, 2H, C*H*$_2$CH=CH_2), 2.10 (dq, *J* = 14.9, 7.5 Hz, 1H, C*H*HCH_3), 1.82 (dq, *J* = 14.7, 7.4 Hz, 1H, CH*H*CH_3), 1.16 (t, *J* = 7.5 Hz, 3H, CH_2C*H*$_3$). ^{13}C NMR (101 MHz, $CDCl_3$): δ = 169.0 (s, N*C*=O), 157.2 (s, Ar), 132.0 (s, H*C*=CH_2), 129.5 (s, Ar), 124.5 (q, *J* = 281.0 Hz,CF_3), 120.4 (s, Ar), 120.3 (s, HC=*C*H_2), 114.5 (s, Ar), 61.1 (s, *C*CHCF_3), 60.1 (q, *J* = 33.0 Hz, *C*HCF_3), 55.6 (s, O*C*H_3), 36.5 (s, *C*H=CH_2), 21.9 (s, CH_2CH_3), 8.9 (s, CH_2CH_3). ^{19}F NMR (376 MHz, $CDCl_3$): δ = –66.83 (d, *J* = 6.7 Hz). HRMS (ESI) *m/z:* $[M + H]^+$ Calcd for $C_{16}H_{18}F_3NO_2H^+$ 314.1368; Found 314.1373.

Racemic mixture of (3R,4S)-3-benzyl-1-(4-methoxyphenyl)-3-methyl-4-(trifluoromethyl)azetidin-2-one (1h)

The racemic mixture **1h** was obtained as a pale yellow oil (131 mg, 64%). To the reaction the mixture of *cis/trans* *N*-PMP 3-Me-4-CF_3-*β*-lactam (152 mg, 0.59 mmol, 1 eq.), benzyl bromide (63 µL, 0.53 mmol, 0.9 eq.) and LiHMDS (0.88 mL, 0.88 mmol, 1.5 eq.) were used, following the general procedure. The crude product was purified *via* column chromatography, using mixture of cyclohexane/ethyl acetate 90:10, v/v.

^{1}H NMR (300 MHz, $CDCl_3$): δ = 7.25 – 7.12 (m, 5H, Ph), 6.94 (d, *J* = 8.9 Hz, 2H, Ar), 6.72 (d, *J* = 8.8 Hz, 2H, Ar), 4.08 (q, *J* = 6.7 Hz, 1H, C*H*CF_3) 3.69 (s, 3H, OC*H*$_3$), 3.14 (d, *J* = 14.0 Hz, 1H,

C*H*HPh), 2.83 (d, *J* = 14.0 Hz, 1H, CH*H*Ph), 1.46 – 1.43 (m, 3H, CH_3). ^{13}C NMR (75 MHz, $CDCl_3$): δ = 169.5 (s, N*C*=O), 157.5, 135.4, 130.1, 129.0, 128.8, 127.6 (6 × s, Ar, Ph), 125.8 (dd, *J* = 280.6, CF_3), 121.1, (d, *J* = 2.9 Hz, Ar), 114.4 (s, Ar), 59.3 (q, *J* = 32.8 Hz, *C*HCF_3), 58.4 (br s, *C*CH_3), 55.6 (s, OCH_3), 42.3 (s, *C*H_2Ph), 16.3 (d, *J* = 3.0 Hz, CCH_3). ^{19}F NMR (188 MHz, $CDCl_3$): δ = –67.21 (d, *J* = 6.4 Hz). HRMS (ESI) *m/z:* $[M + H]^+$ Calcd $C_{19}H_{18}F_3NO_2H^+$ 350.1368; Found 350.1371.

Racemic mixture of (3R,4S)-3-benzyl-3-ethyl-1-(4-methoxyphenyl)-4-(trifluoromethyl)azetidin-2-one (1i)

The racemic mixture of **1i** was obtained as a pale yellow oil (119 mg, 59%). To the reaction the mixture of *cis/trans N*-PMP 3-Et-4-CF_3-*β*-lactam (152 mg, 0.56 mmol, 1 eq.), benzyl bromide (59 µL, 0.50 mmol, 0.9 eq.) and LiHMDS (0.84 mL, 0.84 mmol, 1.5 eq.) were used, following the general procedure. The crude product was purified *via* column chromatography, using mixture of *n*-hexane/ethyl acetate 85:15, v/v.

^1H NMR (400 MHz, $CDCl_3$): δ = 7.28 – 7.12 (m, 5H, Ph), 6.87 – 6.83 (m, 2H, Ar), 6.71 – 6.66 (m, 2H, Ar), 4.06 (q, *J* = 6.9 Hz, 1H, C*H*CF_3), 3.67 (s, 3H, OCH_3), 3.25 (d, *J* = 14.1 Hz, 1H, C*H*HPh), 2.81 (d, *J* = 14.1 Hz, 1H, CH*H*Ph), 2.13 (dq, *J* = 14.5, 7.4 Hz, 1H, C*H*HCH_3), 1.80 (dq, *J* =14.6, 7.4 Hz, 1H, CH*H*CH_3), 1.20 (t, *J* = 7.5 Hz, 3H, CH_2CH_3). ^{13}C NMR (101 MHz, $CDCl_3$): δ = 169.3 (s, N*C*=O), 157.4, 135.5, 130.2, 128.9, 128.7, 127.5 (6 × s, Ar, Ph), 124.7 (q, *J* = 281.0 Hz, *C*F_3), 121.4 (s, Ar), 114.3 (s, Ar), 62.4 (s, *C*CHCF_3), 59.2 (q, *J* = 32.8 Hz, *C*HCF_3), 55.5 (s, OCH_3), 38.1 (s, *C*H_2Ph), 23.3 (s, *C*H_2CH_3), 9.2 (s, CH_2CH_3). ^{19}F NMR (376 MHz, $CDCl_3$): δ = −66.87 (d, *J* = 6.9 Hz). HRMS (ESI) *m/z:* $[M + H]^+$ Calcd for $C_{20}H_{20}F_3NO_2H^+$ 364.1524; Found 364.1523.

Racemic mixture of (3S,4S)-3-benzyl-1-(4-methoxyphenyl)-3-phenyl-4-(trifluoromethyl)azetidin-2-one (1m)

The racemic mixture of **1m** was obtained as a pale yellow oil (128 mg, 52%). To the reaction, the mixture of *cis/trans N*-PMP 3-Ph-4-CF_3-*β*-lactam (192 mg, 0.6 mmol, 1 eq.), benzyl bromide (64 µL, 0.54 mmol, 0.9 eq.) and LiHMDS (0.75 mL, 0.75 mmol, 1.5 eq.) were used, following the general procedure. The crude product was purified *via* column chromatography, using mixture of *n*-hexane/ethyl acetate 90:10, v/v.

^1H NMR (400 MHz, $CDCl_3$): δ = 7.54 (d, *J* = 7.5 Hz, 2H, Ph), 7.37 – 7.16 (m, 8H, Ph), 6.77 – 6.71 (m, 2H, Ar), 6.70 – 6.64 (m, 2H, Ar), 4.33 (q, *J* = 6.3 Hz, 1H, C*H*CF_3), 3.68 (s, 3H, OCH_3), 3.42 (d, *J* = 14.0 Hz, 1H, C*H*HPh), 3.34 (d, *J* = 14.0 Hz, 1H, CH*H*Ph). ^{13}C NMR (101 MHz, $CDCl_3$): δ = 167.4 (s, N*C*=O), 157.8, 135.2, 134.7, 130.7, 128.8, 128.6, 128.4, 127.9, 127.8, 127.8 (10 × s, Ar, Ph), 124.2 (q, *J* = 281.5 Hz, CF_3), 122.1 (s, Ar), 114.3 (s, Ar), 66.1 (s, *C*CHCF_3), 60.6 (q, *J* = 31.9 Hz, *C*HCF_3), 55.5 (s, OCH_3), 43.5 (s, *C*H_2Ph). ^{19}F NMR (376 MHz, $CDCl_3$): δ = −68.15 (d, *J* = 6.2 Hz). HRMS (ESI) *m/z:* $[M + Na]^+$ Calcd for $C_{24}H_{20}F_3NO_2Na^+$ 434.1344; Found 434.1342.

General procedure of the dehydrofluorination of 4-CF_3-*β*-lactams

The solution of the corresponding 3,3-disubstituted 4-CF_3-*β*-lactam **1** (1 eq.) in anhydrous THF (2-8 mL) was performed and cooled to –10°C. Then, the solution of LiHMDS (1M in THF, 4-5 eq.) was added, using syringe to the performed solution of *β*-lactam. The reaction mixture was stirred for 45 min to 1.5h (reaction monitored by TLC). When the spot of the substrate was no longer identified on TLC, the reaction was quenched with saturated $NaHCO_3$ aq (2-6 mL). Then, the quenched reaction mixture was extracted with Et_2O (3 × 5-10 mL). After that, the organic layers were combined and dried, using anhydrous $MgSO_4$. Afterwards, the organic phase was filtered and solvent was evaporated under reduced pressure. The crude product was purified *via* column chromatography, using mixture of *n*-hexane/ethyl acetate or cyclohexane/ethyl acetate.

4-(Difluoromethylene)-1-(4-methoxyphenyl)-3,3-dimethylazetidin-2-one (2a)

The solution of the *N*-PMP 3,3-dimethyl-4-CF_3-*β*-lactam **1a** (1.52 g, 5.56 mmol, 1 eq.) in anhydrous THF (8 mL) was performed and cooled to –10°C. Then, the solution of LiHMDS (1M in THF, 22.27 mL, 22.27 mmol, 4 eq) was added, using syringe to the performed solution of *β*-lactam. The reaction mixture was stirred for 45 min to 1.5h (reaction monitored by TLC). When the spot of the substrate was no longer identified on TLC, the reaction was quenched with saturated $NaHCO_3$ aq (6 mL). Then, the quenched reaction mixture was extracted with Et_2O (3 × 10 mL). After that, the organic layers were combined and dried, using anhydrous $MgSO_4$. Afterwards, the organic phase was filtered and solvent was evaporated under reduced pressure. The crude product was purified *via* column chromatography, using mixture of cyclohexane/ethyl acetate 85:15, v/v. The product **2a** was obtained as a pale yellow oil (930 mg, 66%).

^{1}H NMR (300 MHz, $CDCl_3$): δ = 7.32 (d, *J* = 10.4 Hz, 2H, Ar), 6.89 (d, *J* = 8.9 Hz, 2H, Ar), 3.80 (s, 3H, OCH_3), 1.50 (s, 6H, 2 × CH_3). ^{13}C NMR (75 MHz, $CDCl_3$): δ = 169.7 (br d, N*C*=O), 157.9 (s, Ar), 145.2 (dd, *J* = 282.7, 266.4 Hz, CF_2), 127.8, 122.4, 114.5 (3 × s, Ar), 106.8 (dd, *J* = 52.5, 9.5 Hz, *C*=CF_2), 55.7 – 55.6 (m, OCH_3, *C*CH_3), 19.7 (s, CCH_3). ^{19}F NMR (188 MHz, $CDCl_3$): δ = –98.80 (d, *J* = 112.0 Hz, 1F), –116.22 (d, *J* = 112.0 Hz, 1F). HRMS (ESI) *m/z:* $[M + H]^+$ Calcd $C_{13}H_{13}F_2NO_2H^+$ 254.0993; Found 254.1000.

4-(Difluoromethylene)-3,3-diethyl-1-(4-methoxyphenyl)azetidin-2-one (2b)

The product **2b** was obtained as a pale yellow oil (30 mg, 53%) from *N*-PMP 3,3-diethyl-4-CF_3-*β*-lactam **1b** (60 mg, 0.20 mmol, 1 eq.) and LiHMDS (0.80 mL, 0.80 mmol, 4 eq.), following the general procedure. The crude product was purified *via* column chromatography, using mixture of *n*-hexane/ethyl acetate 90:10, v/v.

^{1}H NMR (400 MHz, $CDCl_3$): δ = 7.34 – 7.27 (m, 2H, Ar), 6.93 – 6.88 (m, 2H, Ar), 3.80 (s, 3H, OCH_3), 1.92 – 1.76 (m, 4H, 2 × CH_2CH_3), 1.05 (t, *J* = 7.5 Hz, 6H, 2 × CH_2CH_3). ^{13}C NMR (101 MHz, $CDCl_3$): δ = 168.7 (s, N*C*=O), 158.0 (s, Ar), 145.6 (dd, *J* = 283.0, 266.3 Hz, CF_2), 127.3 (s, Ar), 122.9 (s, Ar), 114.5 (s, Ar), 102.9 (dd, J = 52.8, 9.3 Hz, *C*=CF_2), 65.9 (dd, *J* = 3.0, 2.0 Hz, *C*C=CF_2), 55.6 (s, OCH_3), 25.4 (s, CH_2CH_3), 9.2 (s, CH_2CH_3).^{19}F NMR (376 MHz, $CDCl_3$): δ = −98.59 (d, *J* = 111.5 Hz, 1F), −115.28 (d, *J* = 111.6 Hz, 1F). HRMS (ESI) *m/z:* $[M + H]^+$ Calcd for $C_{15}H_{17}F_2NO_2H^+$ 282.1306; Found 282.1304.

3,3-Diallyl-4-(difluoromethylene)-1-(4-methoxyphenyl)azetidin-2-one (2c)

The product **1c** was obtained as a pale yellow oil (20 mg, 38%) from *N*-PMP 3,3-diallyl-4-CF_3-*β*-lactam **1c** (57 mg, 0.17 mmol, 1 eq.) and LiHMDS (0.71 mL, 0.71 mmol, 4 eq.), following the general procedure. The crude product was purified *via* column chromatography, using mixture of cyclohexane/ethyl acetate 90:10, v/v.

^{1}H NMR (600 MHz, $CDCl_3$): δ = 7.18 (d, *J* = 7.3 Hz, 2H, Ar), 6.81 (d, *J* = 8.8 Hz, 2H, Ar), 5.80 – 5.73 (m, 2H, 2 × C*H*=CH_2), 5.12 (d, *J* = 17.0 Hz, 2H, 2 × CH=C*H*H), 5.09 (d, *J* = 10.0 Hz, 2H, 2 × CH=CH*H*), 3.72 (s, 3H, OCH_3), 2.51 (dd, *J* = 14.2, 7.1 Hz, 2H, 2 × C*H*HCH=CH_2), 2.43 (dd, *J* = 14.1, 7.5 Hz, 2H, 2 × CH*H*CH=CH_2). ^{13}C NMR (151 MHz, $CDCl_3$): δ = 167.5 (s, N*C*=O), 158.1 (s, Ar), 146.1 (dd, *J* = 283.3, 266.7 Hz, *C*F_3), 131.7 (s, 2 × *C*H=CH_2), 127.0 (s, Ar), 123.0 (d, *J* = 3.5 Hz), 119.8 (s, 2 × CH=*C*H_2), 114.4 (s, Ar), 102.7 (dd, *J* = 52.4, 10.7. Hz, *C*=CF_2), 63.5 (br t, *J* = 2.8 Hz, *C*C=CF_2), 55.6 (s, O*C*H_3), 36.6 (s, 2 × *C*H_2CH=CH_2). ^{19}F NMR (565 MHz, $CDCl_3$): δ = –98.68 (d, *J* = 109.7 Hz, 1F), –114.7 (d, *J* = 109.7 Hz, 1F). HRMS (ESI) *m/z:* $[M + Na]^+$ Calcd $C_{17}H_{17}F_2NO_2Na^+$ 328.1125; Found: 328.1132.

3,3-Dibenzyl-4-(difluoromethylene)-1-(4-methoxyphenyl)azetidin-2-one (2d)

The product **2d** was obtained as a pale yellow oil (31 mg, 51%) from *N*-PMP 3,3-dibenzyl-4-CF_3-*β*-lactam **1d** (64 mg, 0.15 mmol, 1 eq.) and LiHMDS (0.75 mL, 0.75 mmol, 5 eq.), following the general procedure. The crude product was purified *via* column chromatography, using mixture of *n*-hexane/ethyl acetate 85:15, v/v.

^{1}H NMR (400 MHz, $CDCl_3$): δ = 7.32 – 7.26 (m, 10H, 2 × Ph), 6.65 – 6.61 (m, 2H, Ar), 6.43 – 6.37 (m, 2H, Ar), 3.69 (s, 3H, OCH_3), 3.29 (dd, *J* = 13.8, 1.6 Hz, 2H, 2 × C*H*HPh), 3.03 (d, *J* = 13.8, 2H, 2 × CH*H*Ph). ^{13}C NMR (101 MHz, $CDCl_3$): δ = 167.2 (s, N*C*=O), 158.4 (s, Ar), 146.4 (dd, *J* = 284.0, 265.2 Hz, *C*F_2), 135.6 (s, 2 × Ph), 129.9 (s, 2 × Ph), 128.4 (s, Ar), 127.3 (s, 2 × Ph), 125.9 (s, 2 × Ph), 124.1 (d, *J* = 2.3 Hz, Ar), 114.1 (s, Ar), 102.2 (dd, *J* = 51.0, 10.8 Hz, *C*=CF_2), 67.4 (dd, *J* = 3.4, 2.2 Hz, *C*C=CF_2), 55.5 (s, O*C*H_3), 39.1 (s, 2 × *C*H_2Ph). ^{19}F NMR (565 MHz, $CDCl_3$): δ = −100.79 (d, *J* = 109.5 Hz, 1F), −113.75 (d, *J* = 109.6 Hz, 1F). HRMS (ESI) *m/z:* $[M + H]^+$ Calcd for $C_{25}H_{21}F_2NO_2H^+$ 406.1619; Found 406.1614.

Racemic mixture of (R)-4-(difluoromethylene)-3-ethyl-1-(4-methoxyphenyl)-3-methylazetidin-2-one (rac 2e)

The racemic mixture of **2e** was obtained as a pale yellow oil (31 mg, 55%) from *N*-PMP 3-Et-3-Me-4-CF_3-*β*-lactam **1e** (60 mg, 0.21 mmol, 1 eq.) and LiHMDS (0.84 mL, 0.84 mmol, 4 eq.), following the general procedure. The crude product was purified *via* column chromatography, using mixture of cyclohexane/ethyl acetate 90:10, v/v.

^{1}H NMR (300 MHz, $CDCl_3$): δ = 7.31 (d, *J* = 8.8 Hz, 2H, Ar), 6.89 (d, *J* = 8.9 Hz, 2H, Ar), 3.80 (s, 3H, OCH_3), 1.83 (dp, *J* = 14.2, 6.8 Hz, 2H, CH_2CH$_3$), 1.50 (s, 3H, CCH_3), 1.04 (t, *J* = 7.4 Hz, 3H, CH$_2$CH_3). ^{13}C NMR (75 MHz, $CDCl_3$): δ = 169.2 (br d, *J* = 2.0 Hz, N*C*=O), 158.0, 127.6, 122.7, 114.5 (4 × s, Ar), 145.4 (dd, *J* = 282.8, 266.4 Hz, *C*F_2), 105.0 (dd, *J* = 52.8, 9.4 Hz, *C*=CF_2), 60.5 – 60.4 (m, *C*C=CF_2), 55.6 (s, O*C*H_3), 26.8 (s, *C*H_2CH$_3$), 18.2 (s, C*C*H_3), 9.3 (s, CH$_2$*C*H_3). ^{19}F NMR

(188 MHz, $CDCl_3$) δ = –98.47 (d, *J* = 111.8 Hz, 1F), –115.47 (d, *J* = 111.8 Hz, 1F). HRMS (ESI) *m/z:* $[M + H]^+$ Calcd $C_{14}H_{15}F_2NO_2H^+$ 268.1149; Found 268.1146.

Racemic mixture of (R)-3-allyl-4-(difluoromethylene)-1-(4-methoxyphenyl)-3-methylazetidin-2-one (rac 2f)

The racemic mixture of **2f** was obtained as a colorless oil (20 mg, 33%) from *N*-PMP 3-allyl-3-Me-4-CF_3-*β*-lactam **1f** (64 mg, 0.21 mmol, 1 eq.) and LiHMDS (0.84 mL, 0.84 mmol, 4 eq.), following the general procedure. The crude product was purified *via* column chromatography, using mixture of cyclohexane/ethyl acetate 90:10, v/v.

^1H NMR (400 MHz, $CDCl_3$): δ = 7.29 (d, *J* = 7.9 Hz, 2H, Ar), 6.89 (d, *J* = 8.9 Hz, 2H, Ar), 5.83 (dq, *J* = 71.1, 7.5 Hz, 1H, C*H*=CH_2), 5.27 – 5.09 (m, 2H, CH=CH_2), 3.80 (s, 3H, OCH_3), 2.52 (h, *J* = 7.3 Hz, 2H, CH_2CH=CH_2), 1.50 (s, 3H, CH_3). ^{13}C NMR (101 MHz, $CDCl_3$): δ = 168.6 (s, N*C*=O), 158.0, 127.5, 122.7, 114.5 (4 × s, Ar), 145.7 (dd, *J* = 282.0, 266.6 Hz, *C*F_2), 132.1 (m, 2H, CH=*C*H_2), 119.7 (m, 2H, *C*H=CH_2), 104.9 (dd, *J* = 52.6, 10.0 Hz, *C*=CF_2), 59.5 (br t, *J* = 2.6 Hz, *C*C=CF_2), 55.7 (s, O*C*H_3), 38.1 (s, *C*H_2CH=CH_2), 18.2 (s, C*C*H_3). ^{19}F NMR (188 MHz, $CDCl_3$): δ = –98.49 (d, *J* = 110.0 Hz, 1F), –115.12 (d, *J* = 110.9 Hz, 1F). HRMS (ESI) *m/z:* $[M + H]^+$ Calcd $C_{15}H_{15}F_2NO_2H^+$ 280.1149; Found 280.1148.

Racemic mixture of (R)-3-allyl-4-(difluoromethylene)-3-ethyl-1-(4-methoxyphenyl)azetidin-2-one (rac 2g)

The racemic mixture of **2g** was obtained as a pale yellow oil (21 mg, 37%) from *N*-PMP 3-allyl-3-Et-4-CF_3-*β*-lactam **1g** (61 mg, 0.19 mmol, 1 eq.) and LiHMDS (0.76 mL, 0.76 mmol, 4 eq.), following the general procedure. The crude product was purified *via* column chromatography, using mixture of *n*-hexane/ethyl acetate 90:10, v/v.

^1H NMR (400 MHz, $CDCl_3$): δ = 7.30 – 7.23 (m, 2H, Ar), 6.92 – 6.87 (m, 2H, Ar), 5.84 (ddt, *J* = 17.2, 9.7, 7.2 Hz, 1H, C*H*=CH_2), 5.20 (dq, *J* = 16.9, 1.4 Hz, 1H, CH=C*H*H), 5.17 – 5.13 (m, 1H, CH=CH*H*), 3.80 (s, 3H, OCH_3), 2.64 – 2.43 (m, 2H, CH_2CH=CH_2), 1.92 – 1.75 (m, 2H, C$H_2$$CH_3$), 1.06 (t, *J* = 7.4 Hz, 3H, CH_2CH_3). ^{13}C NMR (101 MHz, $CDCl_3$): δ = 168.1 (d, *J* = 2.1 Hz, N*C*=O), 158.1 (s, Ar), 145.8 (dd, *J* = 282.2, 266.5 Hz, *C*F_2), 132.0 (s, 2H, CH=*C*H_2), 127.2 (s, Ar), 123.0 (d, *J* = 3.5 Hz, Ar), 119.6 (s, 2H, *C*H=CH_2), 114.5 (s, Ar), 102.8 (dd, *J* =52.6, 9.9 Hz, *C*=CF_2), 64.8 (dd, *J* = 3.3, 2.1 Hz, *C*C=CF_2), 55.7 (s, O*C*H_3), 36.8 (s, *C*H_2CH=CH_2), 25.3 (s, *C*$H_2$$CH_3$), 9.1 (s, CH_2*C*H_3). ^{19}F NMR (376 MHz, $CDCl_3$): δ = –98.64 (d, *J* = 110.2 Hz, 1F), –114.94 (d, *J* = 110.2 Hz, 1F). HRMS (ESI) *m/z:* $[M + H]^+$ Calcd $C_{16}H_{17}F_2NO_2H^+$ 294.1306; Found 294.1313.

Racemic mixture of (R)-3-benzyl-4-(difluoromethylene)-1-(4-methoxyphenyl)-3-methylazetidin-2-one (rac 2h)

The racemic mixture of **2h** was obtained as a colorless oil (26 mg, 49%) from *N*-PMP 3-Bn-3-Me-4-CF_3-*β*-lactam **1h** (57 mg, 0.16 mmol, 1 eq.) and LiHMDS (0.82 mL, 0.82 mmol, 5 eq.), following the general procedure. The crude product was purified *via* column chromatography, using mixture of cyclohexane/ethyl acetate 85:15, v/v.

^1H NMR (300 MHz, $CDCl_3$): δ = 7.23 – 7.11 (m, 5H, Ph), 6.81 – 6.76 (m, 2H, Ar), 6.68 (d, *J* = 8.9 Hz, 2H, Ar), 3.67 (s, 3H, OCH_3), 3.06 (dd, *J* = 13.8 and 1.0 Hz, 1H, CH*H*Ph), 2.86 (d, *J* = 13.8 Hz,

1H, C*H*HPh), 1.53 (s, 3H, CH_3). ^{13}C NMR (75 MHz, $CDCl_3$): δ = 168.5 (s, N*C*=O), 158.2 (s, Ar), 145.9 (dd, *J* = 282.5 and 266.0 Hz, CF_2), 135.9, 129.8, 128.4 (3 x s, Ph), 127.2 (s, Ar), 126.7 (s, Ph), 123.3 (d, *J* = 2.8 Hz, Ar), 114.3 (s, Ar), 104.6 (dd, *J* =51.8 and 10.1 Hz, *C*=CF_2), 61.3 (br s, *C*C=CF_2), 55.6 (s, OCH_3), 39.9 (s, CH_2Ph), 18.8 (s, CCH_3). ^{19}F NMR (188 MHz, $CDCl_3$): δ = –99.55 (d, *J* = 110.6 Hz, 1F), –114.75 (d, *J* = 110.7 Hz, 1F). HRMS (ESI) *m/z:* $[M + Na]^+$ Calcd $C_{19}H_{17}F_2NO_2Na^+$ 352.1125; Found 352.1119.

Racemic mixture of ethyl (R)-3-benzyl-4-(difluoromethylene)-3-ethyl-1-(4-methoxyphenyl)azetidin-2-one (rac 2i)

The racemic mixture of **2i** was obtained as a pale yellow oil (31 mg, 50%) from *N*-PMP 3-Bn-3-Et-4-CF_3-*β*-lactam **1i** (66 mg, 0.18 mmol, 1 eq.) and LiHMDS (0.90 mL, 0.90 mmol, 5 eq.), following the general procedure. The crude product was purified *via* column chromatography, using mixture of *n*-hexane/ethyl acetate 90:10, v/v.

^{1}H NMR (600 MHz, $CDCl_3$): δ = 7.26 – 7.09 (m, 5H, Ph), 6.75 (d, *J* = 8.1 Hz, 2H, Ar), 6.68 (d, *J* = 8.9 Hz, 2H, Ar), 3.67 (s, 3H, OCH_3), 3.09 (d, *J* = 13.7 Hz, 1H, C*H*HPh), 2.85 (d, *J* = 13.8 Hz, 1H, CH*H*Ph), 1.92 (dq, *J* = 14.7, 7.3 Hz, 1H, C*H*HCH_3), 1.83 (dq, *J* = 14.7, 7.4 Hz, 1H, CH*H*CH_3), 1.05 (t, *J* = 7.4 Hz, 3H, CH_2CH_3). ^{13}C NMR (151 MHz, $CDCl_3$): δ = 168.0 (d, *J* = 1.8 Hz, N*C*=O), 158.2 (s, Ar), 146.0 (dd, *J* = 283.5 and 265.7 Hz, *C*F_2), 135.8, 129.8, 128.4, 127.2, 126.5 (6 × s, Ph, Ar), 123.6 (d, *J* = 2.7 Hz, Ar), 114.3 (s, Ar), 102.5 (dd, *J* =51.6, 10.0 Hz, *C*=CF_2), 66.8 (br t, *J* = 2.5 Hz, *C*C=CF_2), 55.6 (s, OCH_3), 38.7 (s, *C*H_2Ph), 26.0 (s, *C*H_2CH_3), 9.4 (s, CH_2*C*H_3). ^{19}F NMR (565 MHz, $CDCl_3$): δ = –99.77 (d, *J* = 110.5 Hz, 1F), –114.49 (d, *J* = 110.6 Hz, 1F). HRMS (ESI) *m/z:* $[M + H]^+$ Calcd $C_{20}H_{19}F_2NO_2H^+$ 344.1462; Found 344.1466.

Racemic mixture of (S)-4-(difluoromethylene)-1-(4-methoxyphenyl)-3-methyl-3-phenylazetidin-2-one (rac 2j)

The racemic mixture of **2j** was obtained as a pale yiellow oil (29 mg, 47%) from *N*-PMP 3-Me-3-Ph-4-CF_3-*β*-lactam **1j** (65 mg, 0.19 mmol, 1 eq.) and LiHMDS (0.78 mL, 0.78 mmol, 5 eq.), following the general procedure. The crude product was purified *via* column chromatography, using mixture of *n*-hexane/ethyl acetate 90:10, v/v.

^{1}H NMR (400 MHz, $CDCl_3$): δ = 7.51 (d, *J* = 7.6 Hz, 2H, Ar), 7.43 – 7.31 (m, 5H, Ph) 6.95 – 6.88 (m, 2H, Ar), 3.81 (s, 3H, OCH_3), 1.88 (s, 3H, CH_3). ^{13}C NMR (101 MHz, $CDCl_3$): δ = 167.7 (s, N*C*=O), 158.2, (s, Ar) 146.0 (dd, *J* = 283.4, 268.0 Hz, *C*F_2), 137.3 (s, Ph), 129.0 (s, Ar) 128.1, 127.3 (2 × s, Ph) 126.0 (br d, *J* = 1.5 Hz, Ar), 123.0 (d, *J* = 3.7 Hz, Ar), 114.5 (s, Ar), 106.4 (dd, *J* = 51.1, 11.0 Hz, *C*=CF_2), 63.1 (br t, *J* = 2.5 Hz, *C*C=CF_2), 55.6 (s, OCH_3), 21.0 (s, CCH_3). ^{19}F NMR (376 MHz, $CDCl_3$): δ = –99.15 (d, *J* = 107.8 Hz, 1F), –112.73 (d, *J* = 107.9 Hz, 1F). HRMS (ESI) *m/z:* $[M + Na]^+$ Calcd $C_{18}H_{15}F_2NO_2Na^+$ 338.0963; Found 338.0976.

Racemic mixture of (S)-4-(difluoromethylene)-3-ethyl-1-(4-methoxyphenyl)-3-phenylazetidin-2-one (rac 2k)

The racemic mixture of **2k** was obtained as a pale yellow oil (25 mg, 42%) from *N*-PMP 3-Et-3-Ph-4-CF_3-*β*-lactam **1k** (63 mg, 0.18 mmol, 1 eq.) and LiHMDS (0.72 mL, 0.72 mmol, 4 eq.), following

the general procedure. The crude product was purified *via* column chromatography, using mixture of *n*-hexane/ethyl acetate 95:5, v/v.

^{1}H NMR (400 MHz, $CDCl_3$): δ = 7.55 – 7.50 (m, 2H, Ar), 7.43 – 7.29 (m, 5H, Ph), 6.95 – 6.88 (m, 2H, Ar), 3.81 (s, 3H, OCH_3), 2.31 – 2.11 (m, 2H, C$H_2$$CH_3$). 1.10 (t, *J* = 7.4 Hz, 3H, CH_2CH_3). ^{13}C NMR (101 MHz, $CDCl_3$): δ = 167.1 (s, N*C*=O), 158.3, (s, Ar) 146.3 (dd, *J* = 283.4, 267.3 Hz, *C*F_2), 143.6, 137.0 (2 × s, Ph), 128.9 (s, Ar) 128.0 (s, Ph), 126.4 (d, *J* = 2.1 Hz, Ph) 123.5 (d, *J* = 3.5 Hz, Ar), 114.4 (s, Ar), 104.3 (dd, *J* =50.8, 11.2 Hz, *C*=CF_2), 68.5 (br t, *J* = 2.5 Hz, *C*C=CF_2), 55.6 (s, OCH_3), 28.6 (s, *C*$H_2$$CH_3$), 9.7 (s, CH_2*C*H_3). ^{19}F NMR (376 MHz, $CDCl_3$): δ = –98.99 (d, *J* = 107.8 Hz, 1F), –111.40 (d, *J* = 107.9 Hz, 1F). HRMS (ESI) *m/z:* $[M + H]^+$ Calcd $C_{19}H_{17}F_2NO_2H^+$ 330.1300; Found 330.1313.

Racemic mixture of (S)-3-Allyl-4-(difluoromethylene)-1-(4-methoxyphenyl)-3-phenylazetidin-2-one (rac 2l)

The racemic mixture of **2l** was obtained as a pale yellow oil (22 mg, 36%) from *N*-PMP 3-allyl-3-Ph-4-CF_3-*β*-lactam **1l** (64 mg, 0.18 mmol, 1 eq.) and LiHMDS (0.71 mL, 0.71 mmol, 4 eq.), following the general procedure. The crude product was purified *via* column chromatography, using mixture of *n*-hexane/ethyl acetate 90:10, v/v.

^{1}H NMR (400 MHz, $CDCl_3$): δ = 7.57 – 7.50 (m, 2H, Ar), 7.45 – 7.27 (m, 5H, Ph) 6.94 – 6.87 (m, 2H, Ar), 5.88 (ddt, *J* = 17.0, 1.4 Hz, 1H, C*H*=CH_2), 5.27 (dd, *J* = 17.2, 10.1, 7.3 Hz, 1H, CH=C*H*H), 5.23 – 5.18 (m, 1H, CH=CH*H*), 3.80 (s, 3H, OCH_3), 2.94 (ddq, 1H, *J* = 14.2, 7.0, 1.4 Hz, CH*H*CH=CH_2), 2.84 (dd, 1H, *J* = 14.2, 7.6 Hz, C*H*HCH=CH_2). ^{13}C NMR (101 MHz, $CDCl_3$): δ = 166.5 (s, N*C*=O), 158.3, (s, Ar) 146.6 (dd, *J* = 283.6, 267.5 Hz, *C*F_2), 136.5 (s, Ph), 131.7 (s, *C*H=CH_2), 129.0 (s, Ar) 128.2, 126.9 (2 × s, Ph), 126.4 (d, *J* = 2.0 Hz, Ph) 123.5 (d, *J* = 3.4 Hz, Ar), 120.3 (s, CH=*C*H_2), 114.4 (s, Ar), 103.9 (dd, *J* =50.8, 11.8 Hz, *C*=CF_2), 67.4 (br t, *J* = 2.6 Hz, *C*C=CF_2), 55.6 (s, O*C*H_3), 39.5 (s, *C*H_2CH=CH_2). ^{19}F NMR (376 MHz, $CDCl_3$): δ = –99.04 (d, *J* = 108.6 Hz, 1F), –111.21 (d, *J* = 107.3 Hz, 1F). HRMS (ESI) *m/z:* $[M + Na]^+$ Calcd $C_{20}H_{17}F_2NO_2Na^+$ 364.1120; Found 364.1128.

Racemic mixture of (S)-3-benzyl-4-(difluoromethylene)-1-(4-methoxyphenyl)-3-phenylazetidin-2-one (rac 2m)

The racemic mixture of **2m** was obtained as a yellow oil (32 mg, 51%) from *N*-PMP 3-Bn-3-Ph-4-CF_3-*β*-lactam **1m** (66 mg, 0.16 mmol, 1 eq.) and LiHMDS (0.80 mL, 0.80 mmol, 5 eq.), following the general procedure. The crude product was purified *via* column chromatography, using mixture of *n*-hexane/ethyl acetate 85:15, v/v.

Pale yellow oil (32 mg, 51%): ^{1}H NMR (600 MHz, $CDCl_3$): δ = 7.62 (d, *J* = 7.6 Hz, 2H, Ph), 7.44 (t, *J* = 7.6 Hz, 2H, Ph), 7.38 – 7.27 (m, 6H, Ph, Ar), 6.78 – 6.71 (m, 4H, Ph, Ar), 3.74 (s, 3H, OCH_3), 3.51 (dd, *J* = 13.9, 1.5 Hz, 1H, C*H*HPh), 3.24 (d, *J* = 13.9 Hz, 1H, CH*H*Ph). ^{13}C NMR (151 MHz, $CDCl_3$): δ = 166.4 (d, *J* = 1.0 Hz, N*C* = O), 158.4, (s, Ar) 146.7 (dd, *J* = 282.4, 267.4 Hz, *C*F_2), 136.8, 135.3, 130.0, 129.0, 128.4, 128.1, 127.4, 126.4, 126.0, 124.1, 114.3 (11 × s, Ph, Ar) 103.7 (dd, *J* = 49.6, 9.5 Hz, *C*=CF_2), 69.0 (br s, *C*C=CF_2), 55.4 (s, OCH_3), 41.6 (s, *C*H_2Ph). ^{19}F NMR (565 MHz, $CDCl_3$): δ = –100.37 (dd, *J* = 107.5, 1.3 Hz, 1F), –110.45 (d, *J* = 107.4 Hz, 1F). HRMS (ESI) *m/z:* $[M + Na]^+$ Calcd $C_{24}H_{19}F_2NO_2Na^+$ 414.1282; Found 414.1277.

Racemic mixture of ethyl (R)-2-(difluoromethylene)-1-(4-methoxyphenyl)-3-methyl-4-oxoazetidine-3-carboxylate (rac 2o)

The racemic mixture of **2o** was obtained as a colorless oil (28 mg, 47%) from *N*-PMP 3-carboxylate-3-Me-4-CF_3-*β*-lactam **1o** (63 mg, 0.19 mmol, 1 eq.) and LiHMDS (0.76 mL, 0.76 mmol, 4 eq.), following the general procedure. The crude product was purified *via* column chromatography, using mixture of *n*-hexane/ethyl acetate 90:10, v/v.

^{1}H NMR (400 MHz, $CDCl_3$): δ = 7.36 – 7.30 (m, 2H, Ar), 6.94 – 6.90 (m, 2H, Ar), 4.27 (q, *J* = 7.2 Hz, 2H, OCH_2CH_3), 3.81 (s, 3H, OCH_3), 1.75 (s, 3H, CCH_3), 1.30 (t, *J* = 7.1 Hz, 3H, OCH_2CH_3).^{13}C NMR (101 MHz, $CDCl_3$): δ = 167.1 (s, N*C*=O), 162.4 (d, *J* = 1.1, O*C*=O) 158.4 (s, Ar), 145.8 (dd, *J* = 283.4, 270.6 Hz, CF_2), 127.2, 114.5 (2 × s, Ar), 122.8 (d, *J* = 3.5 Hz, Ar), 103.0 (dd, *J* = 51.3, 15.0 Hz, *C*=CF_2), 63.1 (br t, *J* = 2.6 Hz, *C*C=CF_2), 62.5 (s, OCH_2CH_3), 55.7 (s, OCH_3), 15.3 (s, OCH_2CH_3), 14.1 (s, CCH_3). ^{19}F NMR (376 MHz, $CDCl_3$) δ = –98.52 (d, *J* = 103.6 Hz, 1F), –113.25 (d, *J* = 103.5 Hz, 1F). HRMS (ESI) *m/z:* $[M + Na]^+$ Calcd $C_{15}H_{15}F_2NO_4Na^+$ 334.0867; Found 334.0868.

General procedure of the rearrangement reaction of the formylate group

The solution of the corresponding 3,3-disubstituted 4-CF_3-*β*-lactam **1n, p** (1 eq.) in anhydrous THF (4 mL) was performed and cooled to –10°C. Then, the solution of LiHMDS (1M in THF, 4 eq.) was added, using syringe to the performed solution of *β*-lactam. The reaction mixture was stirred for 45 min to 1.5h (reaction monitored by TLC). When the spot of the substrate was no longer identified on TLC, the reaction was quenched with saturated $NaHCO_3$ aq (3 mL). Then, the quenched reaction mixture was extracted with Et_2O (3 × 7 mL). After that, the organic layers were combined and dried, using anhydrous $MgSO_4$. Afterwards, the organic phase was filtered and solvent was evaporated under reduce pressure. The crude product was purified *via* column chromatography, using mixture of *n*-hexane/ethyl acetate.

Racemic mixture of diethyl (2R,3R)-1-(4-methoxyphenyl)-4-oxo-2-(trifluoromethyl)-azetidine-2,3-dicarboxylate (rac 3)

The racemic mixture of **3** was obtained as a pale yellow oil (38 mg, 57%) from *N*-PMP diethyl 3,3-dicarboxylate-4-CF_3-*β*-lactam **2n** (67 mg, 0.17 mmol, 1 eq.) and LiHMDS (0.67 mL, 0.67 mmol, 4 eq.), following the general procedure. The crude product was purified *via* column chromatography, using mixture of *n*-hexane/ethyl acetate 65:35, v/v.

^{1}H NMR (600 MHz, $CDCl_3$): δ = 7.38 (d, *J* = 8.9 Hz, 2H, Ar), 6.88 (d, *J* = 8.9 Hz, 2H, Ar), 4.45 (s, 1H, C*H*CCF_3), 4.37 – 4.30 (m, 2H, OCH_2CH_3), 4.27 (dq, *J* = 7.1, 0.9 Hz, 2H, OCH_2CH_3), 3.80 (s, 3H, OCH_3), 1.32 (t, *J* = 7.2 Hz, 3H, OCH_2CH_3), 1.28 (t, *J* = 7.1 Hz, 3H, OCH_2CH_3). ^{13}C NMR (151 MHz, $CDCl_3$): δ = 163.2 (s, N*C*=O), 163.1 (s, O*C*=O), 158.2 (s, O*C*=O), 158.0, 128.1 (2 x s, Ar), 122.9 (q, *J* = 284.1 Hz, CF_3), 122.6, 114.3 (2 x s, Ar), 66.3 (q, *J* = 31.8 Hz, *C*CF_3), 63.5 (s, OCH_2CH_3), 62.7 (s, OCH_2CH_3), 59.8 (br s, *C*CCF_3), 55.5 (s, OCH_3), 14.0 (s, OCH_2CH_3), 13.8 (s, OCH_2CH_3).^{19}F NMR (565 MHz, $CDCl_3$): δ = –70.97 (s, CF_3). HRMS (ESI) *m/z:* $[M + Na]^+$ Calcd $C_{17}H_{18}F_3NO_6Na^+$ 412.0984; Found 412.0977.

Racemic mixture of ethyl (2R,3R)-1-(4-methoxyphenyl)-4-oxo-3-phenyl-2-(trifluoromethyl)azetidine-2-carboxylate (rac 5)

The racemic mixture of **5** was obtained as pale yiellow oil (28 mg, 46%) from *N*-PMP 3-carboxylate-3-Ph-4-CF_3-*β*-lactam **1m** (61 mg, 0.15 mmol, 1 eq.) and LiHMDS (0.60 mL, 0.60 mmol, 4 eq.), following the general procedure. The crude product was purified *via* column chromatography, using mixture of *n*-hexane/ethyl acetate 80:20, v/v.

^{1}H NMR (600 MHz, $CDCl_3$): δ = 7.47 – 7.42 (m, 2H, Ar), 7.40 – 7.31 (m, 5H, Ph), 6.93 – 6.86 (m, 2H, Ar), 4.95 (s, 1H, C*H*CCF$_3$), 3.87 – 3.76 (m, 5H, OC*H*$_2$CH$_3$, OC*H*$_3$), 0.82 (t, *J* = 7.1 Hz, 3H, OCH$_2$C*H*$_3$). ^{13}C NMR (151 MHz, $CDCl_3$): δ = 163.6 (s, O*C*=O), 163.2 (s, N*C*=O), 157.8, 129.5, 129.2, 129.1, 128.9, 128.8 (6 × s, Ar, Ph), 123.6 (q, *J* = 283.5, CF$_3$), 121.8, 114.5 (2 × s, Ar), 70.3 (q, *J* = 30.9 Hz, *C*CF$_3$), 62.9 (s, O*C*H$_2$CH$_3$), 61.9 (br s, *C*CCF$_3$), 55.6 (s, O*C*H$_3$), 13.4 (s, OCH$_2$*C*H$_3$).^{19}F NMR (565 MHz, $CDCl_3$): δ = –69.65 (s, CF$_3$). HRMS (ESI) *m/z:* $[M + H]^+$ Calcd $C_{20}H_{18}F_3NO_4H^+$ 394.1204; Found 394.1212.

General procedure of the hydrogenolysis of the *gem*-difluoroalkene group

In the round-bottom flask with a wide neck, 4-difluoromethylene-*β*-lactam **2a,o** (1 eq.) was dissolved in EtOH (2 mL), and a catalytic amount of palladium hydroxide (5-10%) on charcoal was added. Then, the flask was connected by three-way valve to a vacuum pump and a gasbag filled with gaseous hydrogen. Next, hydrogen was introduced inside the flask at 0°C and the mixture was intensively stirred. This cycle was repeated 15 times and the reaction mixture was stirred for 1 h. The catalyst was then filtered out and crude product was isolated using column chromatography (*n*-hexane/ethyl acetate).

Racemic mixture of the 4-(difluoromethyl)-1-(4-methoxyphenyl)-3,3-dimethylazetidin-2-one (rac 8)

The racemic mixture of **8** was obtained as a pale yellow oil (67 mg, 99%) from **2a** (67 mg, 0.25 mmol, 1 eq.) and catalytic amount of palladium hydroxide (9 mg, 0.05 eq.), following the general procedure. The crude product was purified *via* column chromatography, using mixture of *n*-hexane/ethyl acetate 70:30, v/v.

^{1}H NMR (600 MHz, $CDCl_3$): δ = 7.40 (d, *J* = 8.9 Hz, 2H, Ar), 6.88 (d, *J* = 8.9 Hz, 2H, Ar), 5.95 (td, *J* = 54.9, 5.9 Hz, 1H, C*H*F$_2$), 3.95 (dt, *J* = 10.0, 6.1 Hz, 1H, C*H*CHF$_2$), 3.79 (s, 3H, OC*H*$_3$), 1.45 (s, 3H, C*H*$_3$), 1.39 (s, 3H, C*H*$_3$). ^{13}C NMR (151 MHz, $CDCl_3$): δ = 170.4 (s, N*C*=O), 156.7, 130.8 (2 × s, Ar), 119.3 (br d, *J* = 2.5 Hz, Ar), 115.7 (dd, *J* =244.1, 2.5 Hz, *C*HF$_2$), 114.3 (s, Ar), 62.5 (dd, *J* = 30.9, 20.5 Hz, *C*HCHF$_2$), 55.6 (m, O*C*H$_3$), 52.7 (d, *J* = 5.3 Hz, *C*CHCHF$_2$), 23.0 (s, C*C*H$_3$), 17.2 (br d, *J* = 1.9 Hz, *C*CH$_3$). ^{19}F NMR (565 MHz, $CDCl_3$): δ = –118.44 (ddd, *J* = 302.8, 55.5, 9.8 Hz, 1F), –120.34 (ddd, *J* = 302.8, 53.9, 6.0 Hz 1F). HRMS (ESI) *m/z:* $[M + H]^+$ Calcd $C_{13}H_{15}F_2NO_2H^+$ 256.1149; Found 256.1155.

Racemic mixture of the ethyl (2S,3R)-2-(difluoromethyl)-1-(4-methoxyphenyl)-3-methyl-4-oxoazetidine-3-carboxylate (rac 9)

The racemic mixture of **9** was obtained as a pale yellow oil (19 mg, 95%) from **2o** (20 mg, 0.06 mmol) and catalytic amount of palladium hydroxide (8 mg, 0.1 eq.), following the general procedure. The crude product was purified *via* column chromatography, using mixture of *n*-hexane/ethyl acetate 70:30, v/v.

^{1}H NMR (600 MHz, $CDCl_3$): δ = 7.41 (d, *J* = 9.0 Hz, 2H, Ar), 6.90 (d, *J* = 9.1 Hz, 2H, Ar), 6.25 (ddd, *J* = 56.0, 53.3, 5.4 Hz, 1H, C*H*F_2), 4.36 – 4.22 (m, 2H, OC*H*$_2$$CH_3$), 4.10 (ddd, *J* = 12.7, 5.4, 2.8 Hz, 1H, C*H*CHF_2), 3.79 (s, 3H, OC*H*$_3$), 1.71 (s, 3H, C*H*$_3$), 1.32 (t, *J* = 7.2 Hz, 3H, OCH_2C*H*$_3$). ^{13}C NMR (151 MHz, $CDCl_3$): δ = 167.7 (s, N*C*=O), 163.2 (s, O*C*=O) 157.1, 130.1 (2 × s, Ar), 119.6 (d, *J* = 1.9 Hz, Ar), 115.0 (dd, *J* =244.1, 1.1 Hz, *C*HF_2), 114.4 (s, Ar), 62.7 (dd, *J* = 31.2, 23.2 Hz, *C*$HCHF_2$), 62.4 (s, O*C*H_2CH_3), 60.8 (d, *J* = 4.9 Hz, *C*$CHCHF_2$), 55.5 (s, O*C*H_3), 17.9 (s, C*C*H_3), 14.0 (s, OCH_2*C*H_3). ^{19}F NMR (565 MHz, $CDCl_3$): δ = –119.13 (ddd, *J* = 303.0, 56.1, 12.6 Hz, 1F), –121.42 (ddd, *J* = 303.1, 53.3, 2.6 Hz 1F). HRMS (ESI) *m/z:* $[M + H]^+$ Calcd $C_{15}H_{17}F_2NO_4H^+$ 314.1204; Found 314.1214.

Racemic mixture of 1-(4-methoxyphenyl)-4-propyl-azetidin-2-one (rac 10)

In a two-neck flask activated zinc (35 mg, 0.54 mmol, 3 eq.) and the α-bromoester (36 μL, 0.34 mmol, 2 eq.) have been placed. Then, previously prepared [6] imine (26 mg, 0.18 mmol, 1 eq.), in dry THF (1 mL) was added under argon atmosphere. The suspension was slowly warmed using an oil bath until 50°C then to reflux. After 8 h, the mixture was cooled to room temperature and quenched with saturated NH_4Cl aq (1 mL), then extracted with Et_2O (4 × 1.5 mL). The organic layer was dried using anhydrous $MgSO_4$ and filtered. Then, the solvent was concentrated under vacuum and the crude mixture was purified by column chromatography with silica gel (*n*-hexane/ethyl acetate 70:30, v/v).

Pale yellow oil (30.4 mg, 77%): ^{1}H NMR (600 MHz, $CDCl_3$) δ = 7.31 (d, *J* = 8.7 Hz, 2H, Ar), 6.87 (d, *J* = 8.8 Hz, 2H, Ar), 4.02 (ddt, *J* = 8.3, 5.3, 2.7 Hz, 1H, C*H*-*n*-Pr), 3.78 (s, 3H, OC*H*$_3$), 3.15 (dd, *J* = 14.9, 5.3 Hz, 1H, C*H*HCO), 2.71 (dd, *J* = 14.9, 2.1 Hz, 1H, CH*H*CO), 1.59 – 1.47 (m, 1H, C*H*HEt), 1.40 (h, *J* = 7.5 Hz, 2H, C*H*$_2$$CH_3$), 1.27 (dt, *J* = 14.3, 7.0 Hz, 1H, CH*H*Et), 0.97 (t, *J* = 7.3 Hz, 3H, C*H*$_3$). ^{13}C NMR (151 MHz, $CDCl_3$) δ = 164.1 (s, N*C*=O), 156.0, 131.2, 118.5, 114.4 (4 x s, Ar), 55.5 (O*C*H_3), 51.2 (*C*H-*n*-Pr), 42.0 (s, *C*H_2CO), 34.3 (s, *C*H_2Et), 18.3 (s, *C*H_2CH_3), 14,0 (s, *C*H_3). HRMS (ESI) *m/z:* $[M + Na]^+$ Calcd $C_{13}H_{17}NO_2Na^+$ 242.1157; Found 242.1164.

References

1. B. Motherwell, M. J. Tozer, B. C. Ross, *J. Chem. Soc. Chem. Commun.*, (1989), 1437–1439

2 (a) G. Magueur, B. Crousse, M. Ourévitch, D. Bonnet-Delpon, J.-P. Bégué, *J. Fluor. Chem.*, 127, (2006), 637–642 (b) N. A. Meanwell, *J. Med. Chem.*, 54, (2011), 2529–2591, (c) H. Yanai, T. Taguchi, *Eur J. Org. Chem.*, (2011), 5939–5954, (d) J. Nie, H.-C. Guo, D. Cahard, J.-A. Ma, *Chem.*

Rev., 111, (2011), 455–529, (e) C. Alonso, E. Martínez de Marigorta, G. Rubiales, F. Palacios, *Chem. Rev.*, 115, (2015), 1847–1935

3 (a) J. T. Welch, *Selective Fluorination in Organic and Bioorganic Chemistry*, ACS Symposium Series 456; American Chemical Society: Washington, DC, 1991, (b) W. R. Moore, G. L. Schatzman, E. T. Jarvi, R. S. Gross, J. R. McCarthy, *J. Am. Chem. Soc.*, 114, (1992), 360–361, (c) P. M. Weintraub, A. K. Holland, C. A. Gates, W. R. Moores, R. J. Resvick, P. Beye, N. P. Peet, *Bioorg. Med. Chem.*, 11, (2003), 427–431

4 (a) Y. Zafrani, G. Sod-Moriah, D. Yeffet, A. Berliner, D. Amir, D. Marciano, S. Elias, S. Katalan, N. Ashkenazi, M. Madmon, E. Gershonov, S. Saphier *J. Med. Chem., 62* (11), (2019), 5628-5637, (b) Y. Zafrani, D. Yeffet, G. Sod-Moriah, A. Berliner, D. Amir, D. Marciano, E. Gershonov, S. Saphier, *J. Med. Chem.*, 60, (2017), 797−804, (c) C. D. Sessler, M. Rahm, S. Becker, J. M. Goldberg, F. Wang, S. J. Lippard, *J. Am. Chem. Soc.*, 139, (2017), 9325−9332, (d) M. D. Martínez, L. Luna, A. Y. Tesio, G. E. Feresin, F. J. Durán, G. Burton, *J. Pharm. Pharmacol.*, 68, (2016), 233−244, (e) M. A. Chowdhury, K. R. A. Abdellatif, Y. Dong, D. Das, M. R. Suresh, E. E. Knaus, *J. Med. Chem.*, 52, (2009), 1525−1529.

5 (a) J. Wang, M. Sánchez-Roselló, J. L. Aceña, C. del Pozo, A. E. Sorochinsky, S. Fustero, V. A. Soloshonok, H. Liu, *Chem. Rev.*, 114, (2014), 2432−2506, (b) M. Inoue, Y. Sumii, N. Shibata, *ACS Omega*, 5 (19), (2020), 10633-10640

6 R. Annunziata, M. Cinquini, F. Cozzi, P.G. Cozzi, *J. Org.Chem*. 57(15), (1992), 4155–4162

7 M. Skibińska, M. Kaźmierczak, T. Milcent, T. Cytlak, H. Koroniak, B. Crousse, *Front. Chem.*, 7, (2019), article number 526

1. ^{1}H, ^{13}C, ^{19}F NMR Spectra of CF_3-*β*-lactams

Spectrum ^{1}H NMR (400 MHz, $CDCl_3$)

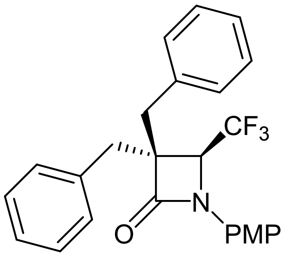

***rac* 1d**

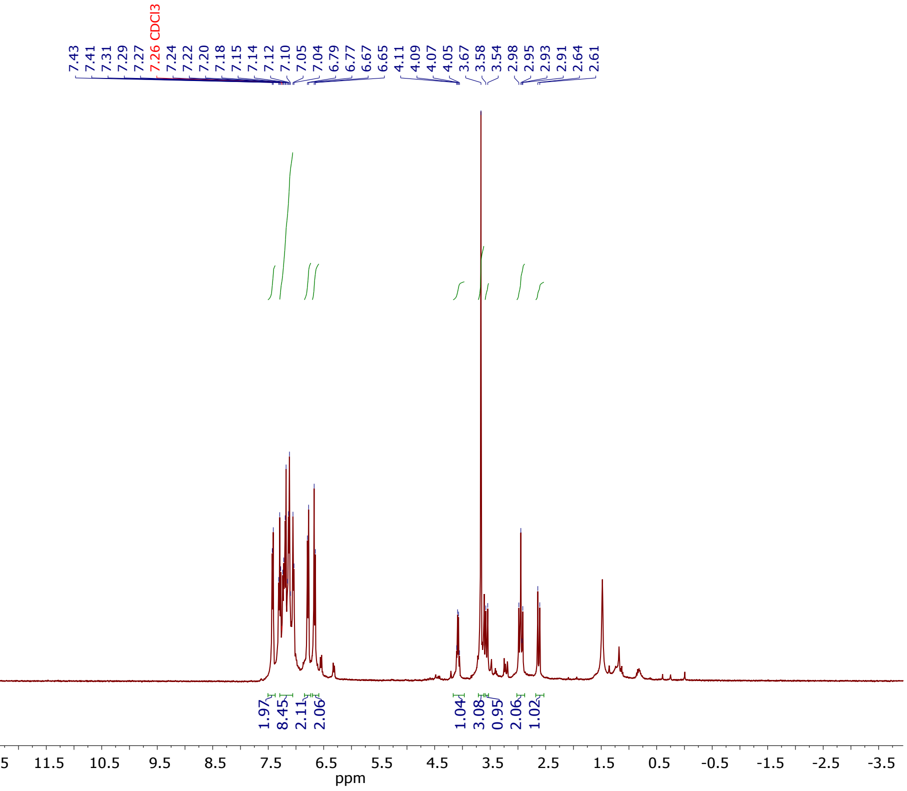

Spectrum ^{13}C NMR (101 MHz, $CDCl_3$)

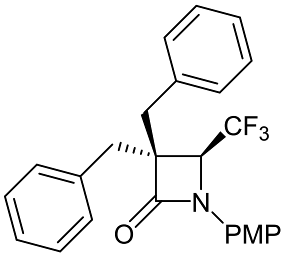

***rac* 1d**

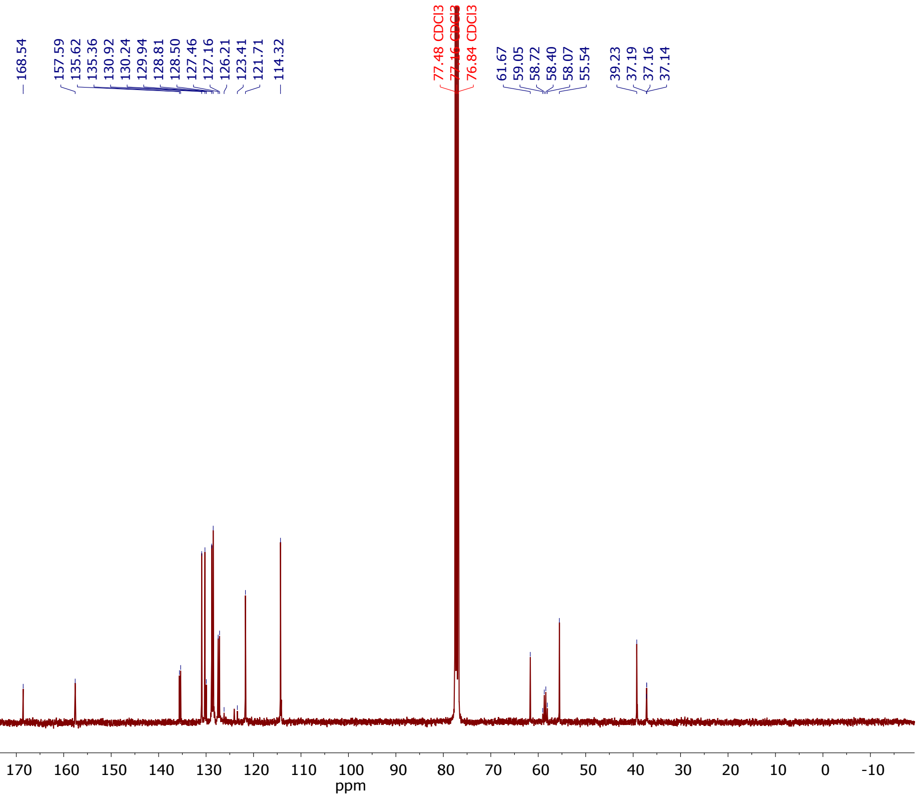

Spectrum ^{19}F NMR (376 MHz, $CDCl_3$)

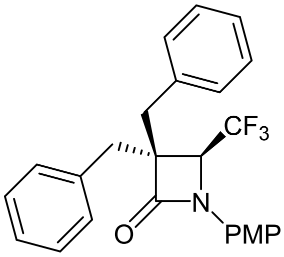

***rac* 1d**

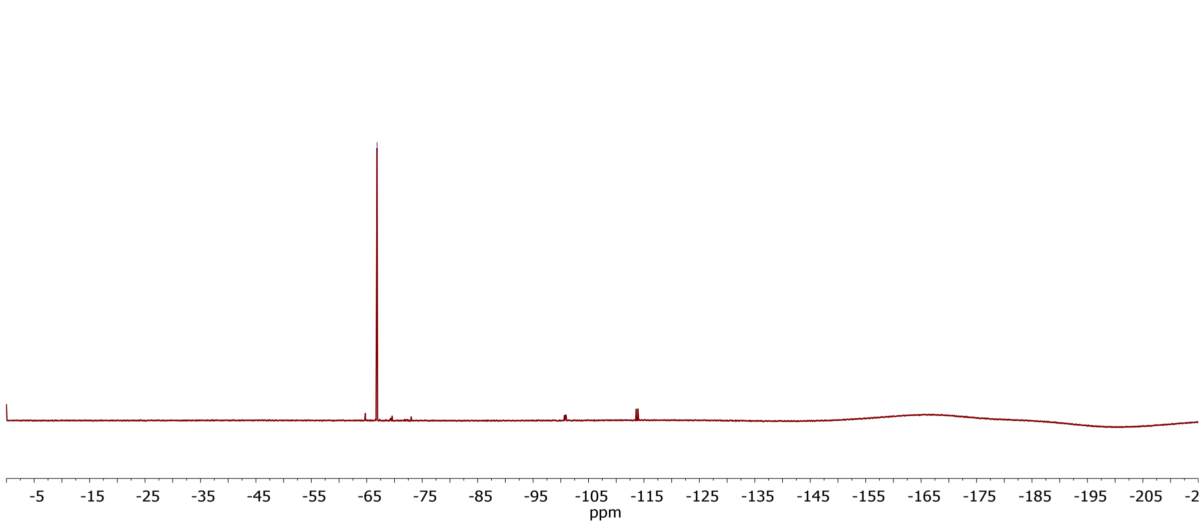

Spectrum ^{1}H NMR (400 MHz, $CDCl_3$)

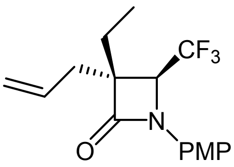

***rac* 1g**

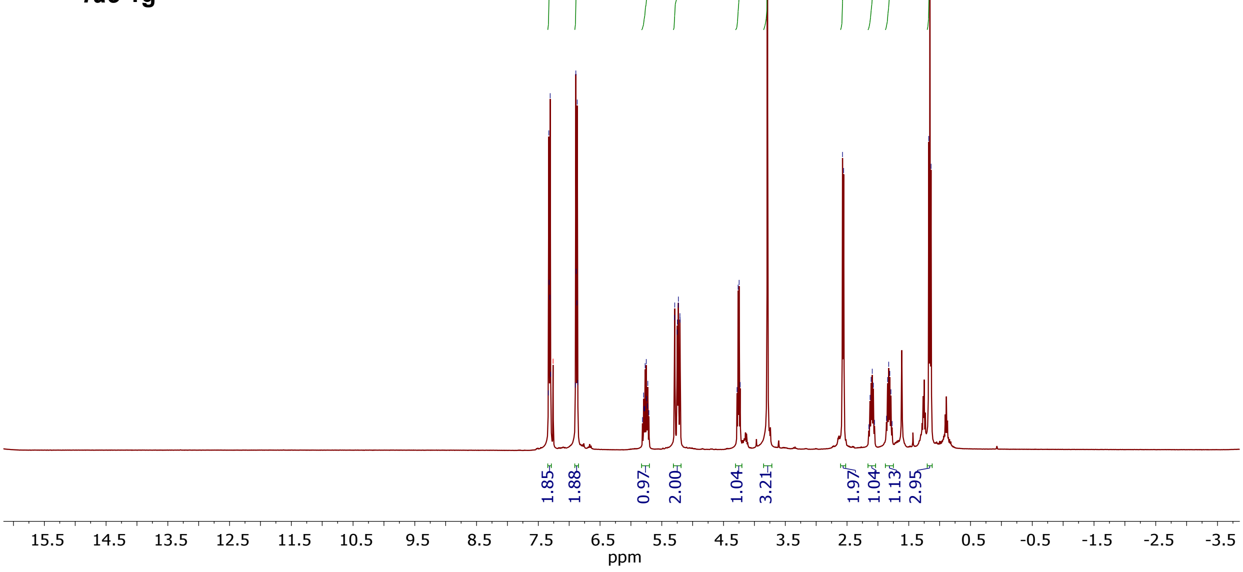

Spectrum ^{13}C NMR (101 MHz, $CDCl_3$)

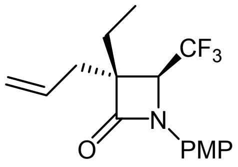

***rac* 1g**

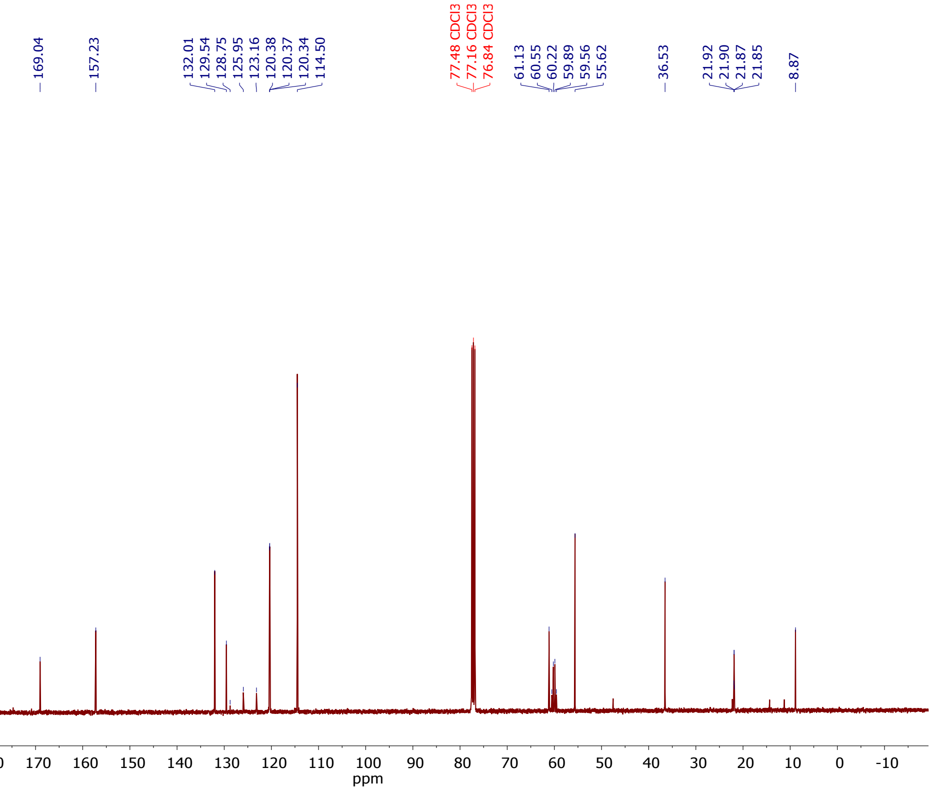

Spectrum ^{19}F NMR (376 MHz, $CDCl_3$)

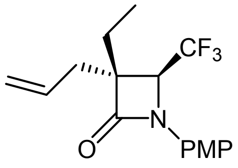

***rac* 1g**

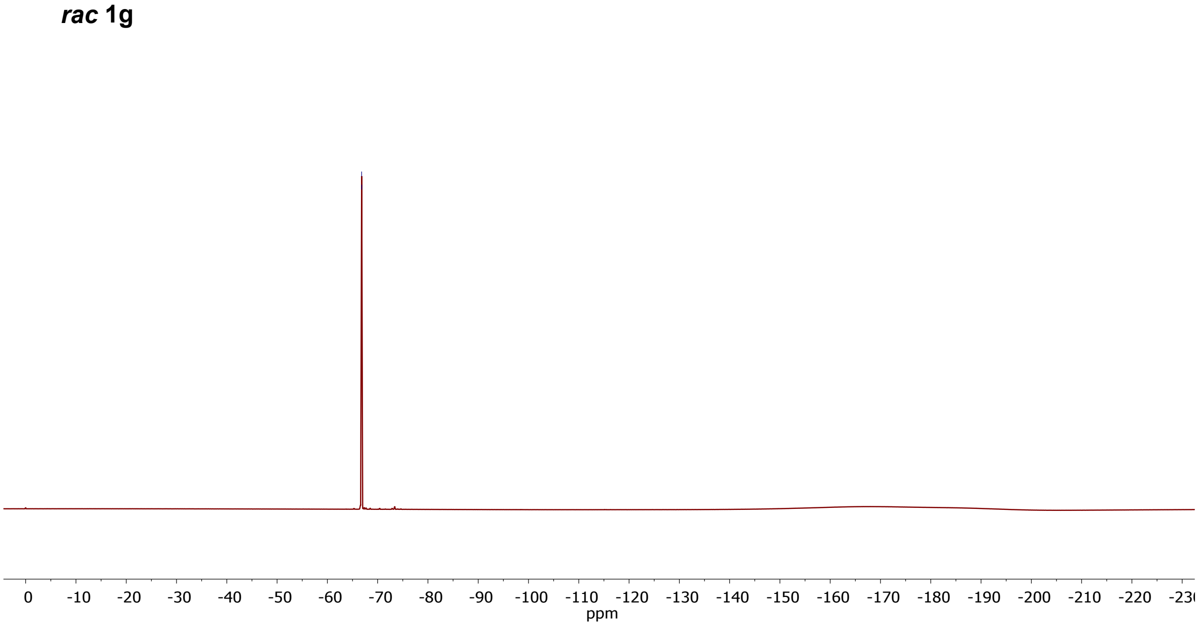

Spectrum ¹H NMR (300 MHz, $CDCl_3$)

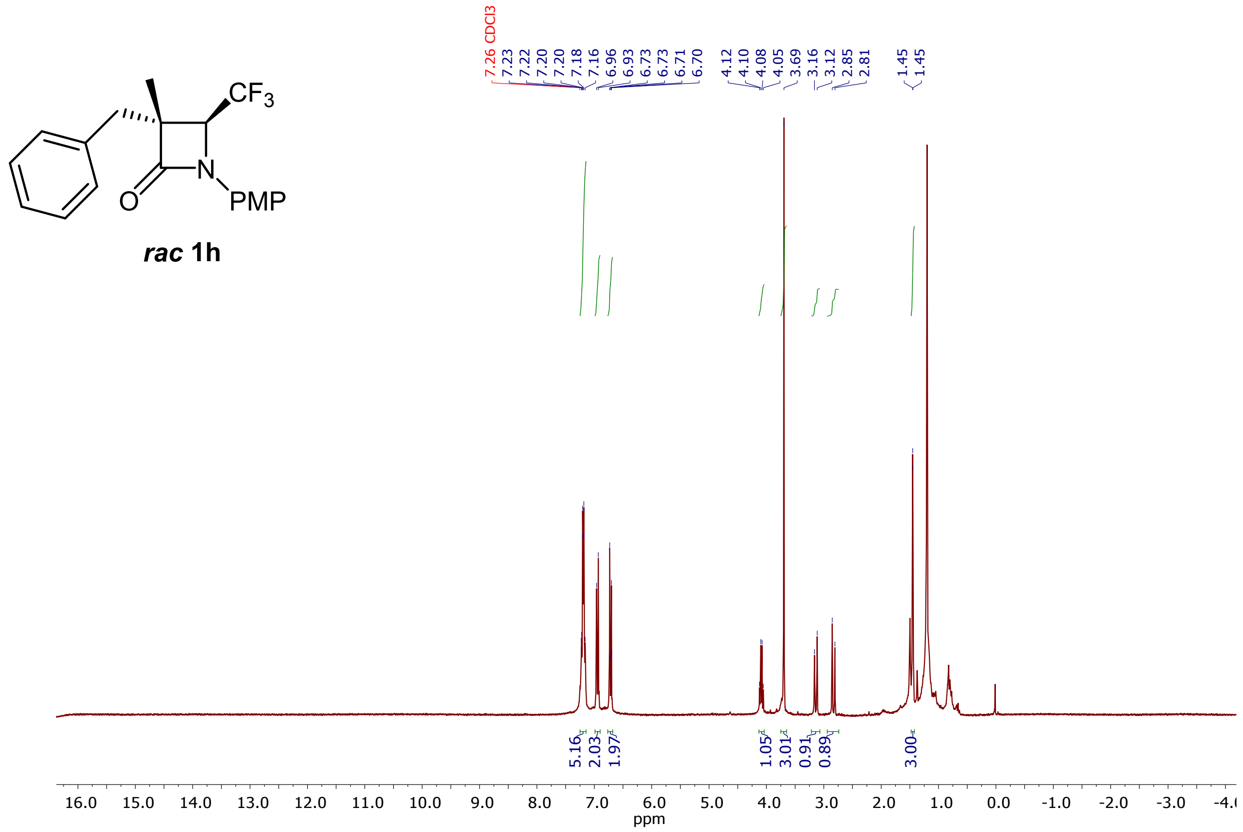

Spectrum ¹³C NMR (75 MHz, $CDCl_3$)

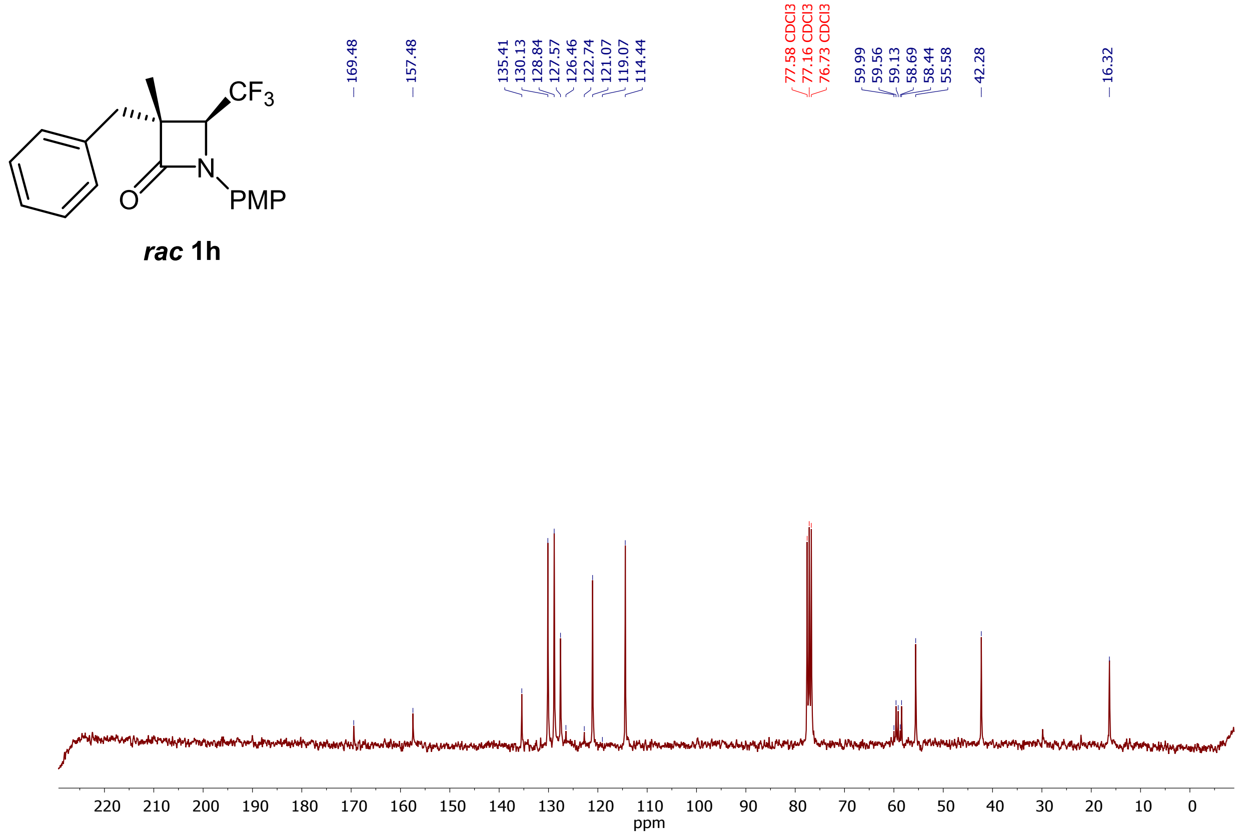

Spectrum ^{19}F NMR (188 MHz, $CDCl_3$)

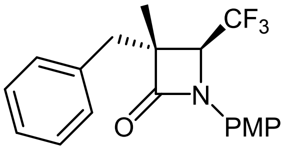

***rac* 1h**

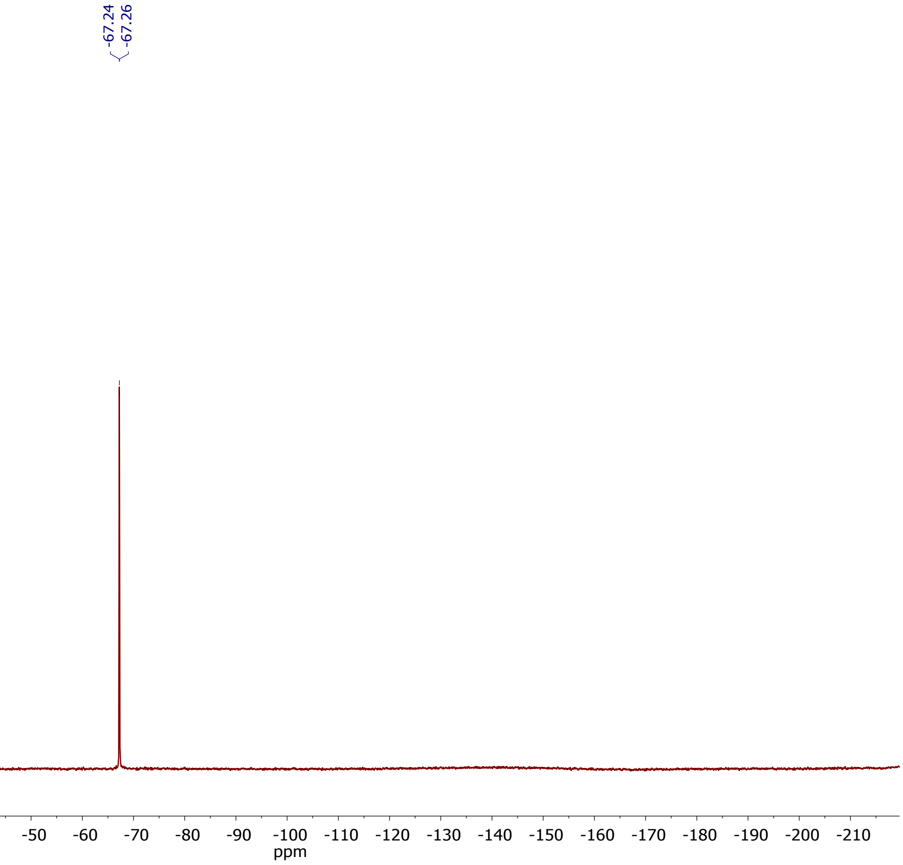

Spectrum ^{1}H NMR (400 MHz, $CDCl_3$)

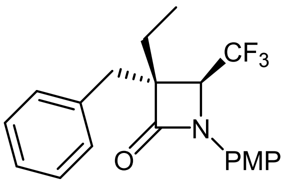

***rac* 1i**

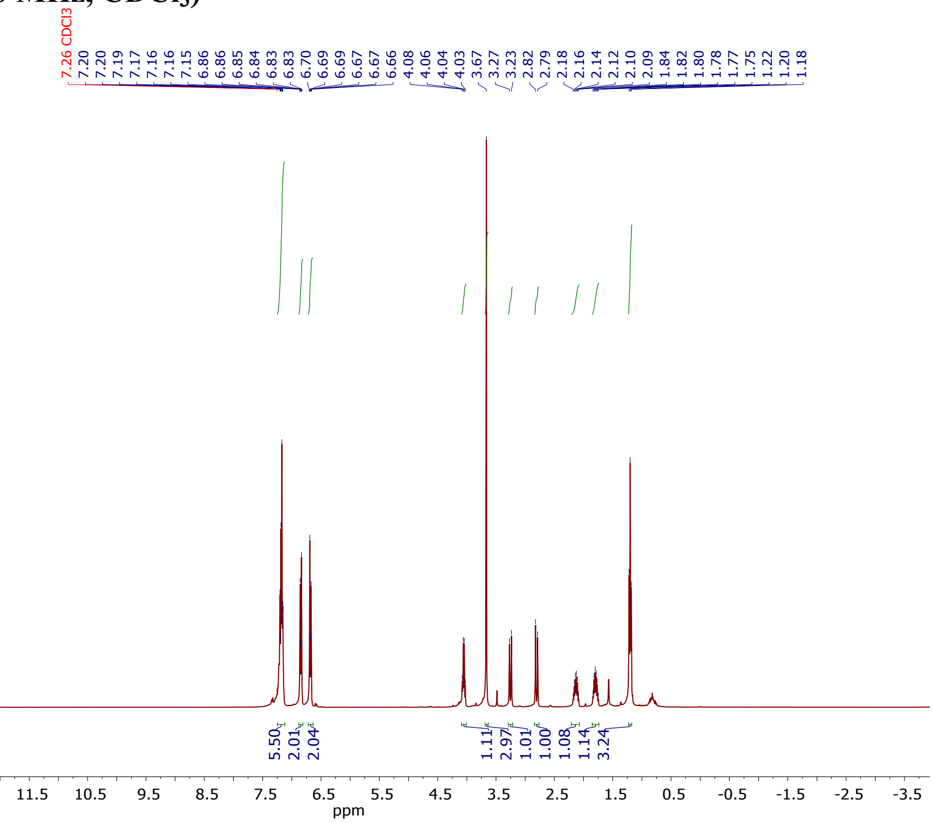

Spectrum ^{13}C NMR (101 MHz, $CDCl_3$)

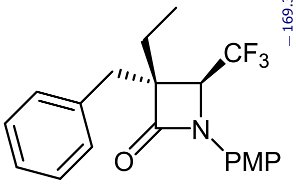

***rac* 1i**

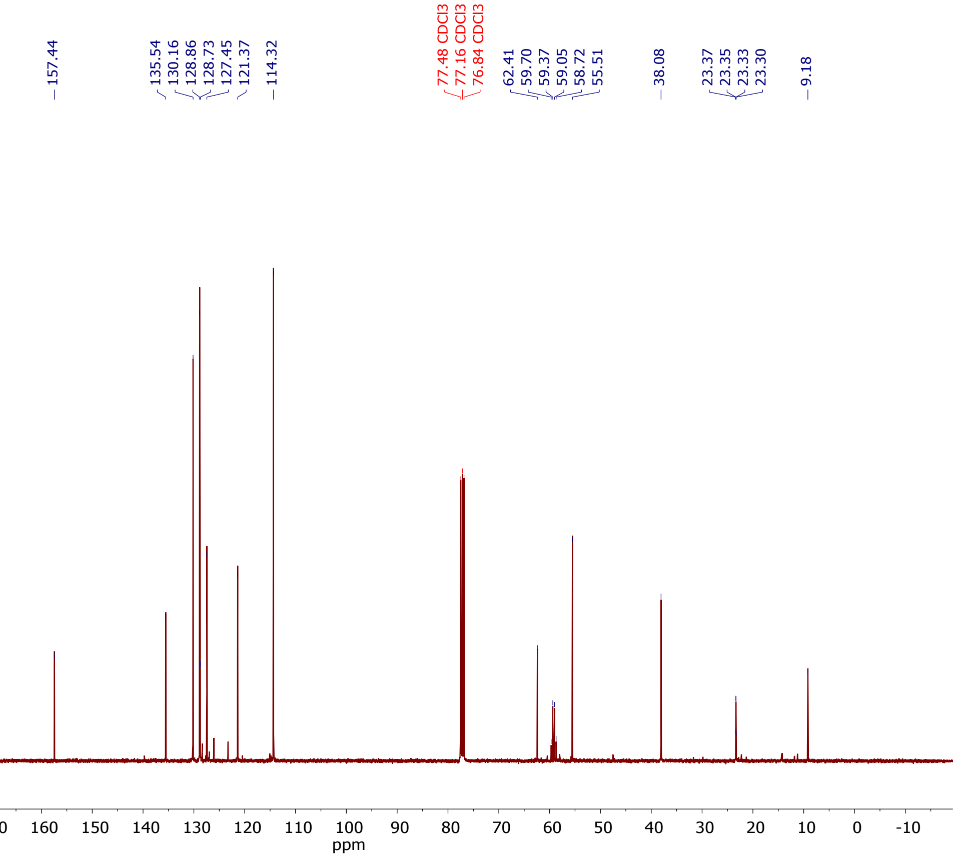

Spectrum ^{19}F NMR (376 MHz, $CDCl_3$)

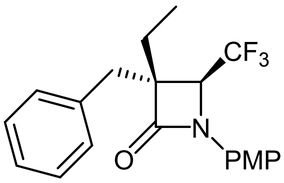

rac **1i**

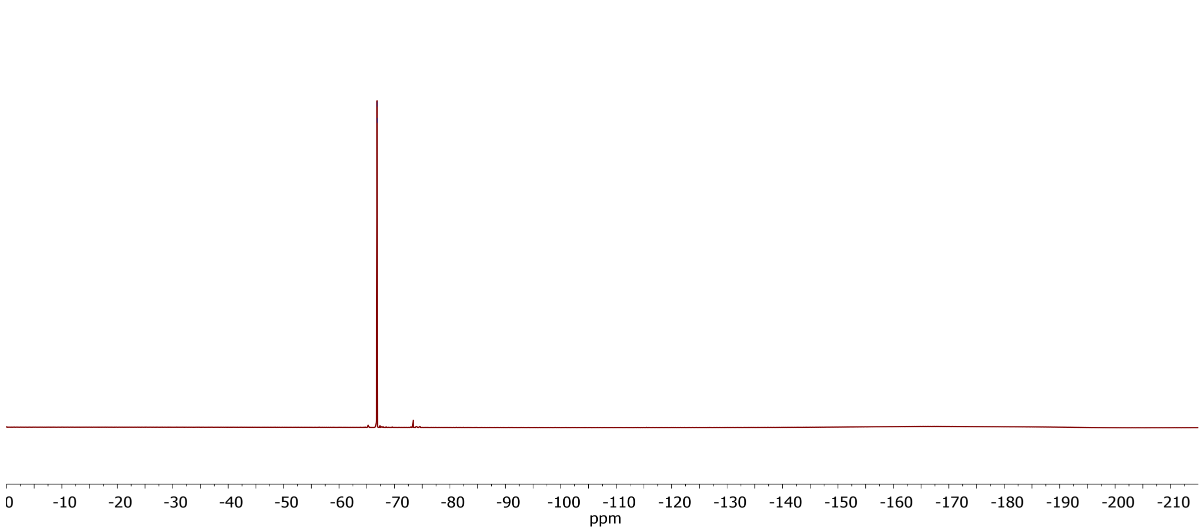

Spectrum ^{1}H NMR (400 MHz, $CDCl_3$)

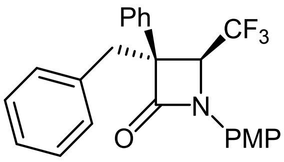

rac **1m**

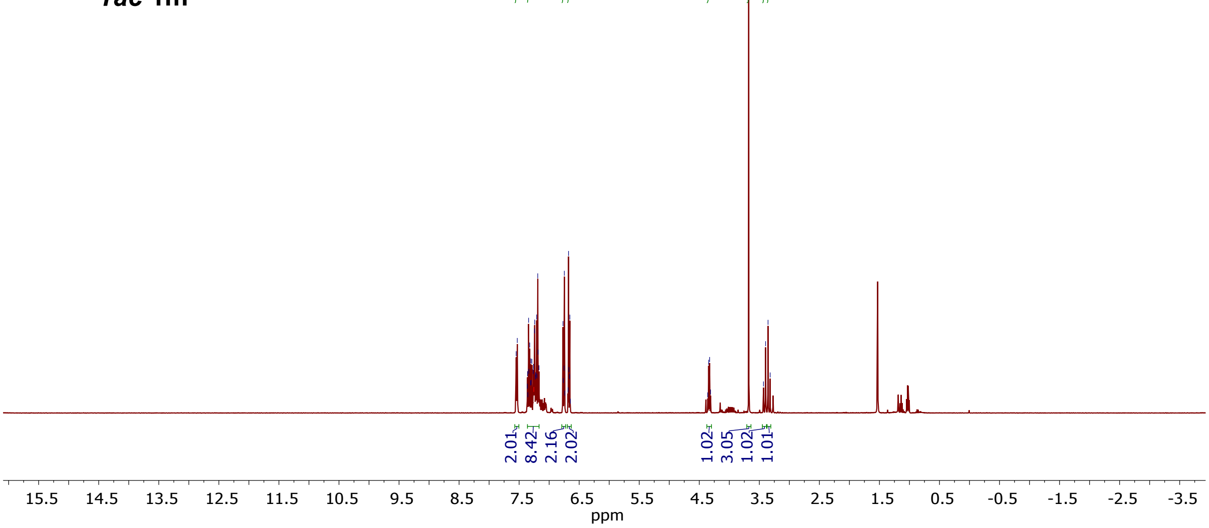

Spectrum ^{13}C NMR (101 MHz, $CDCl_3$)

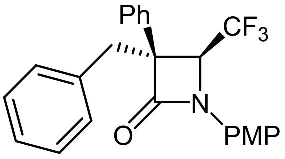

***rac* 1m**

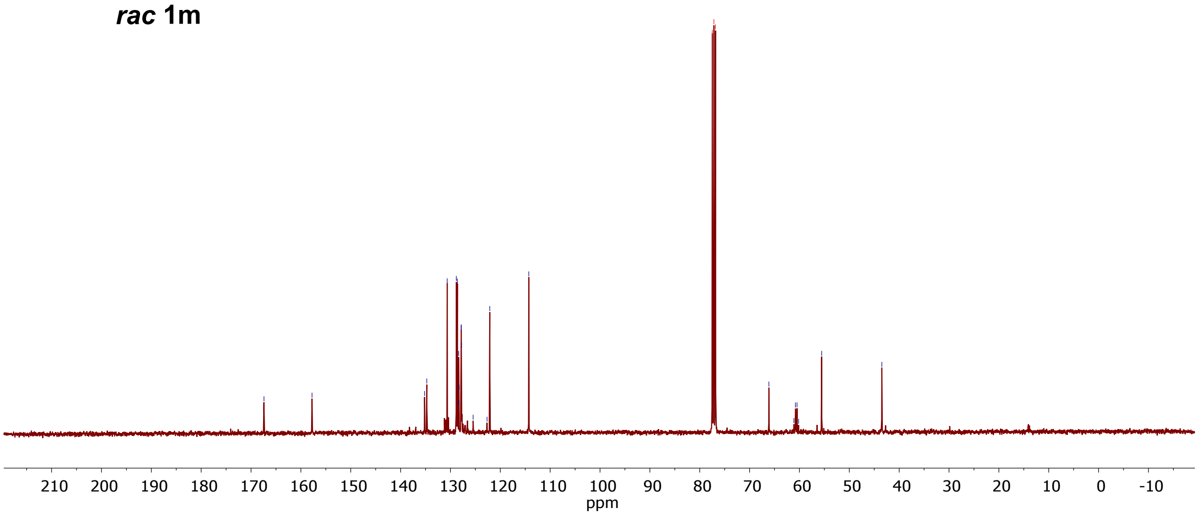

Spectrum ^{19}F NMR (376 MHz, $CDCl_3$)

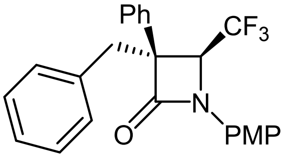

***rac* 1m**

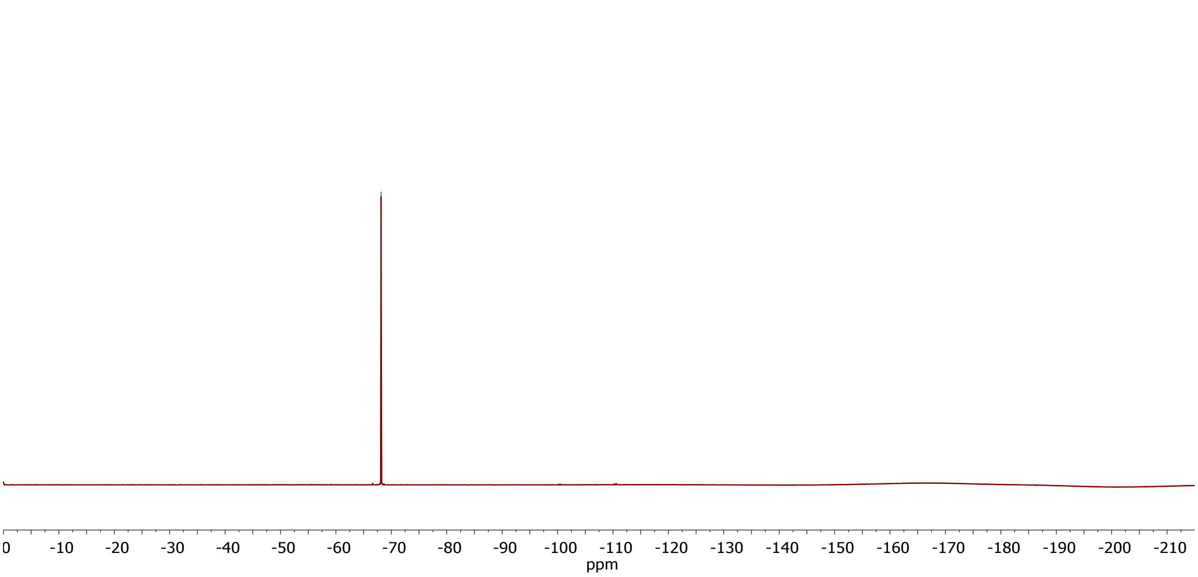

Spectrum ^{1}H NMR (300 MHz, $CDCl_3$)

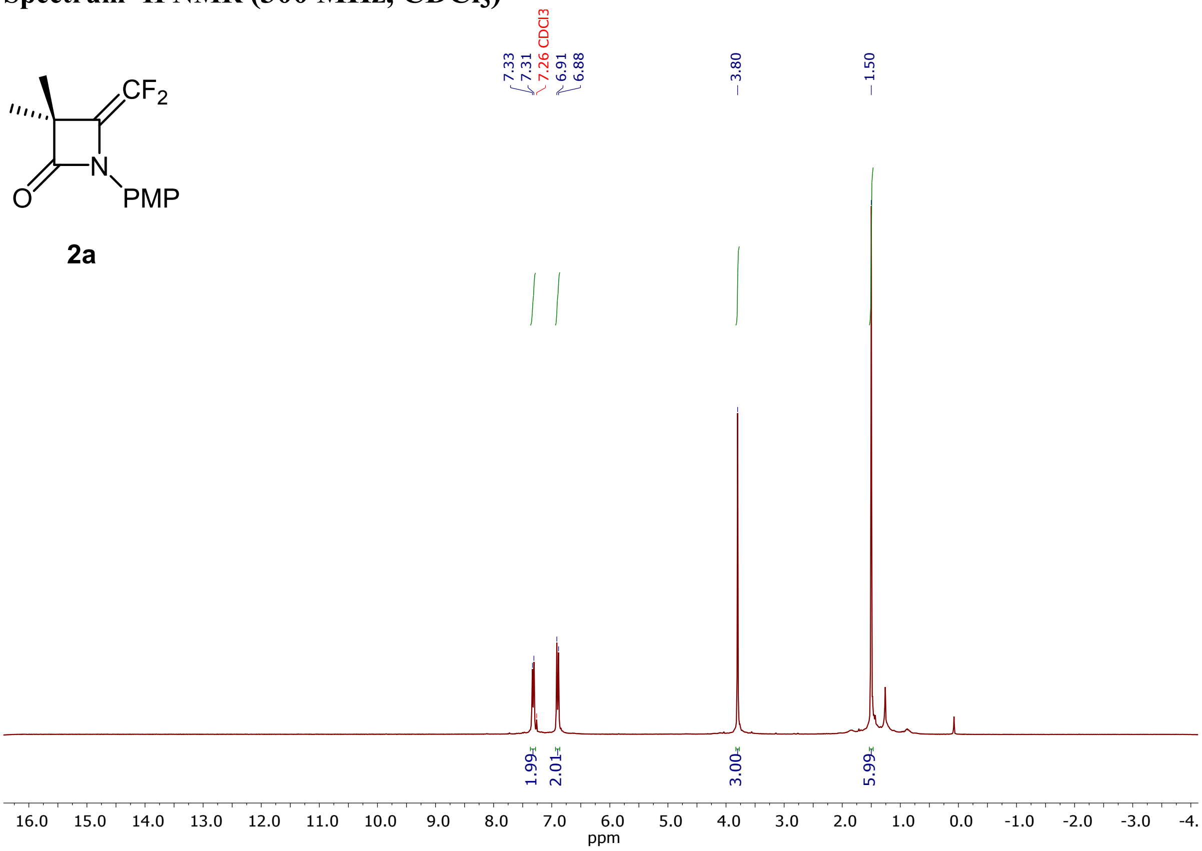

Spectrum ^{13}C NMR (75 MHz, $CDCl_3$)

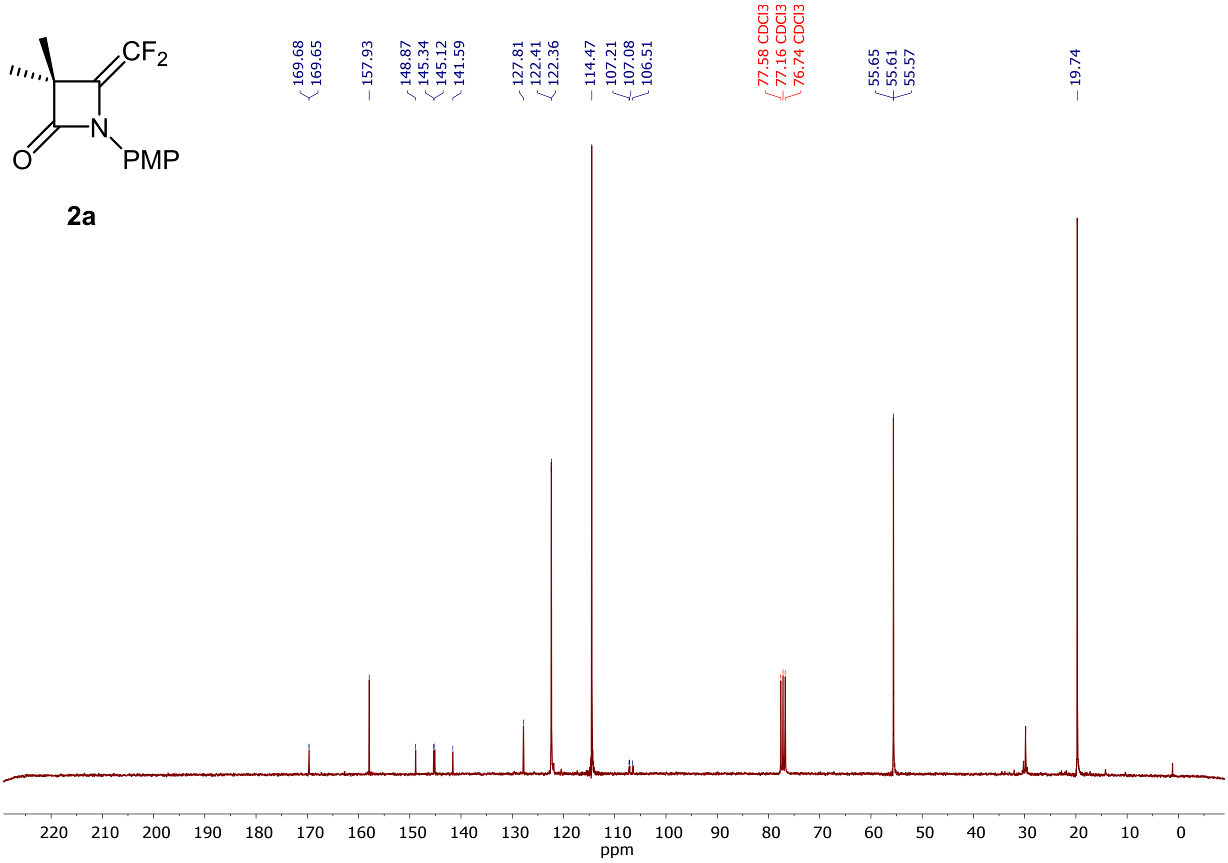

Spectrum ^{19}F NMR (188 MHz, $CDCl_3$)

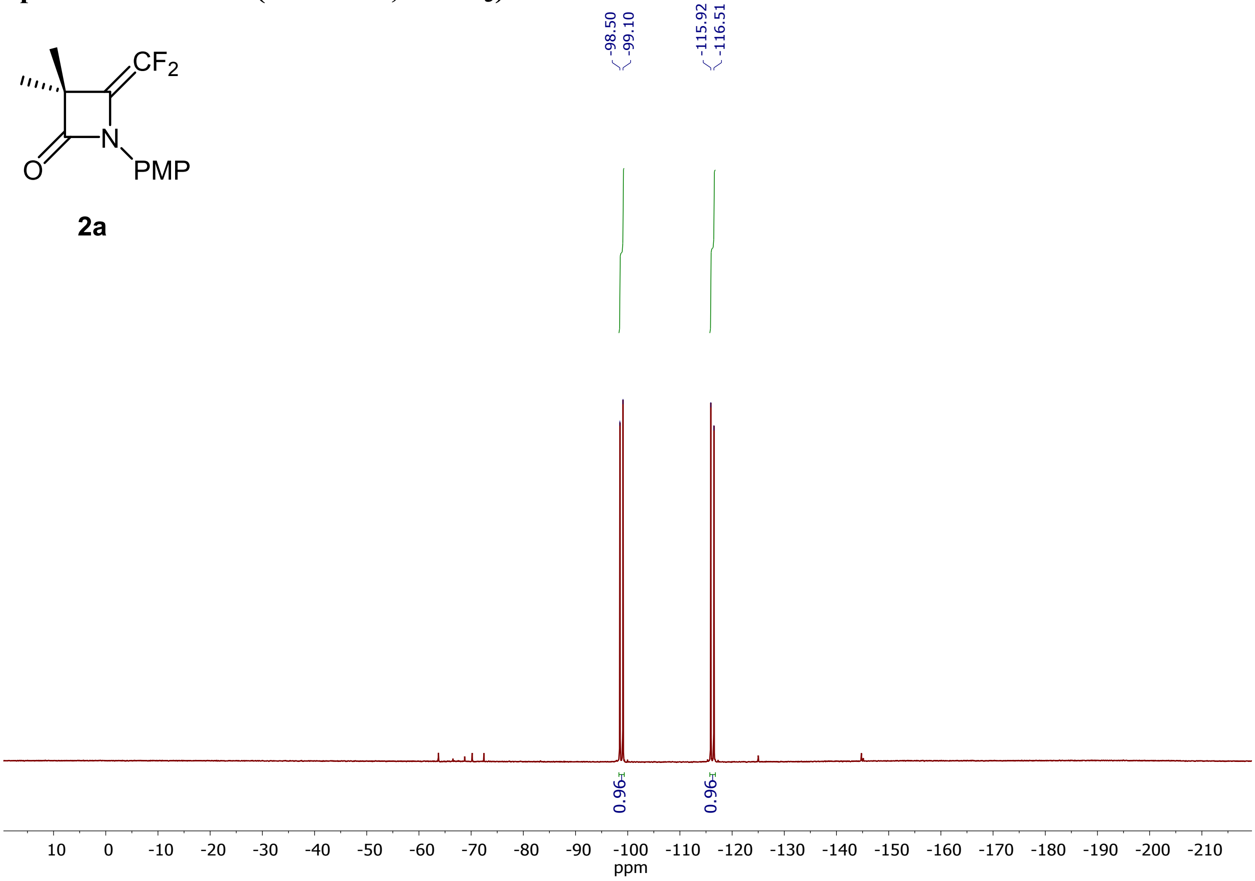

2a

Spectrum ^{1}H NMR (400 MHz, $CDCl_3$)

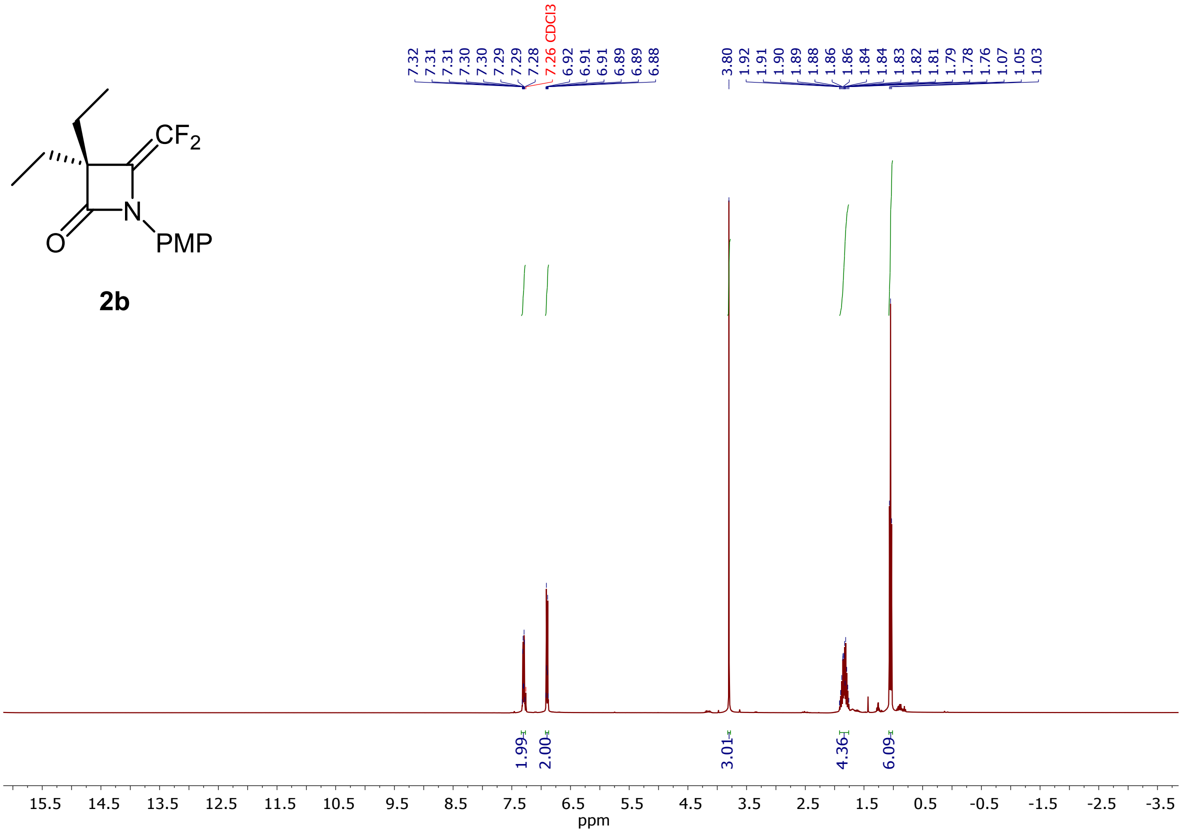

2b

Spectrum ^{13}C NMR (101 MHz, $CDCl_3$)

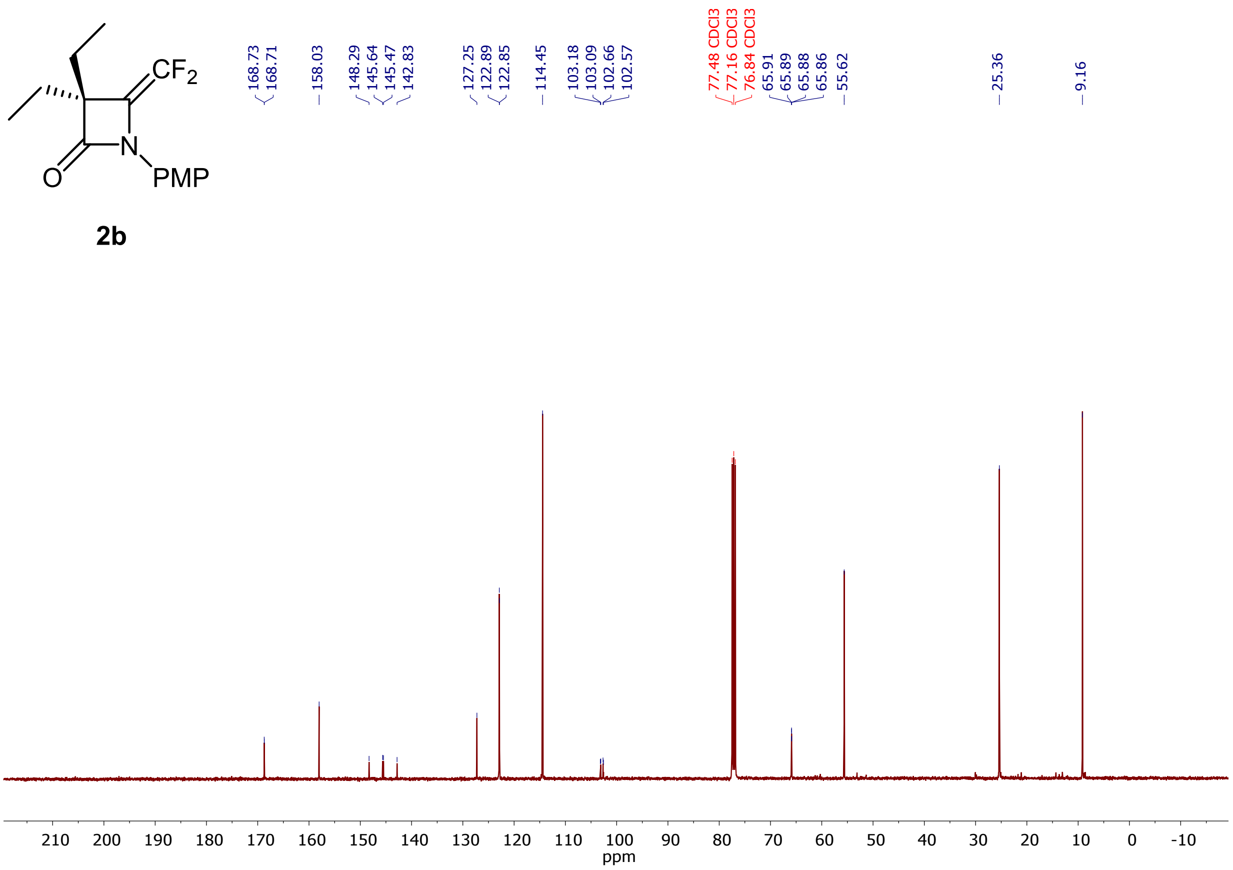

Spectrum ^{19}F NMR (376 MHz, $CDCl_3$)

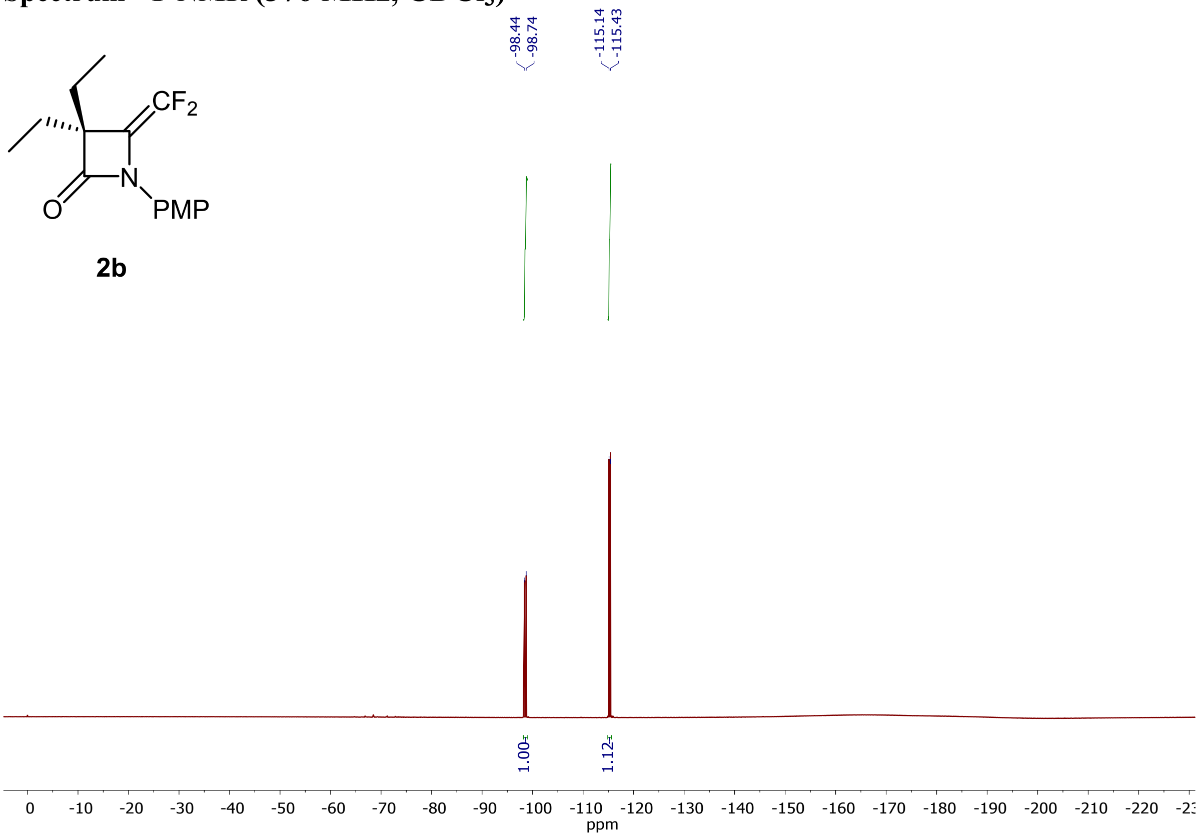

Spectrum ^{1}H NMR (600 MHz, $CDCl_3$)

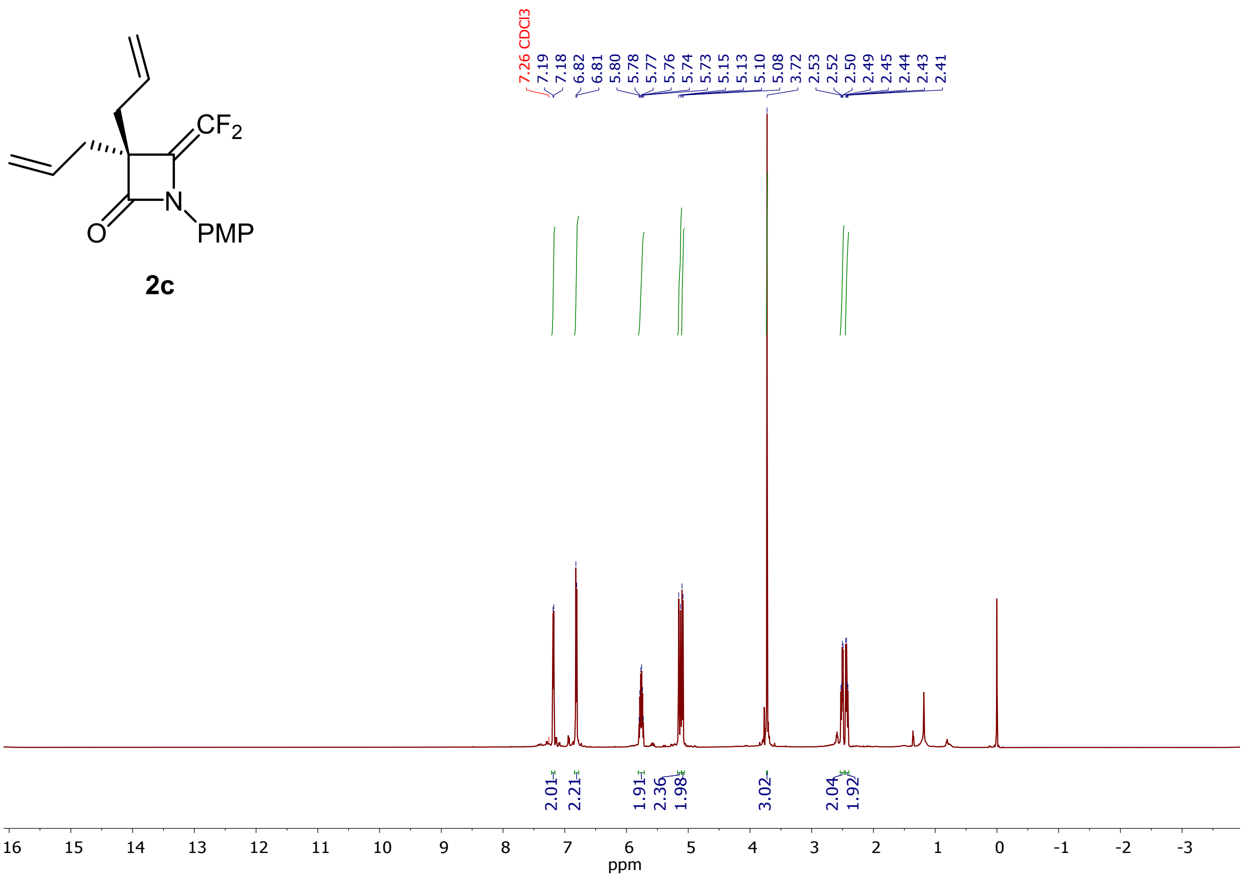

Spectrum ^{13}C NMR (151 MHz, $CDCl_3$)

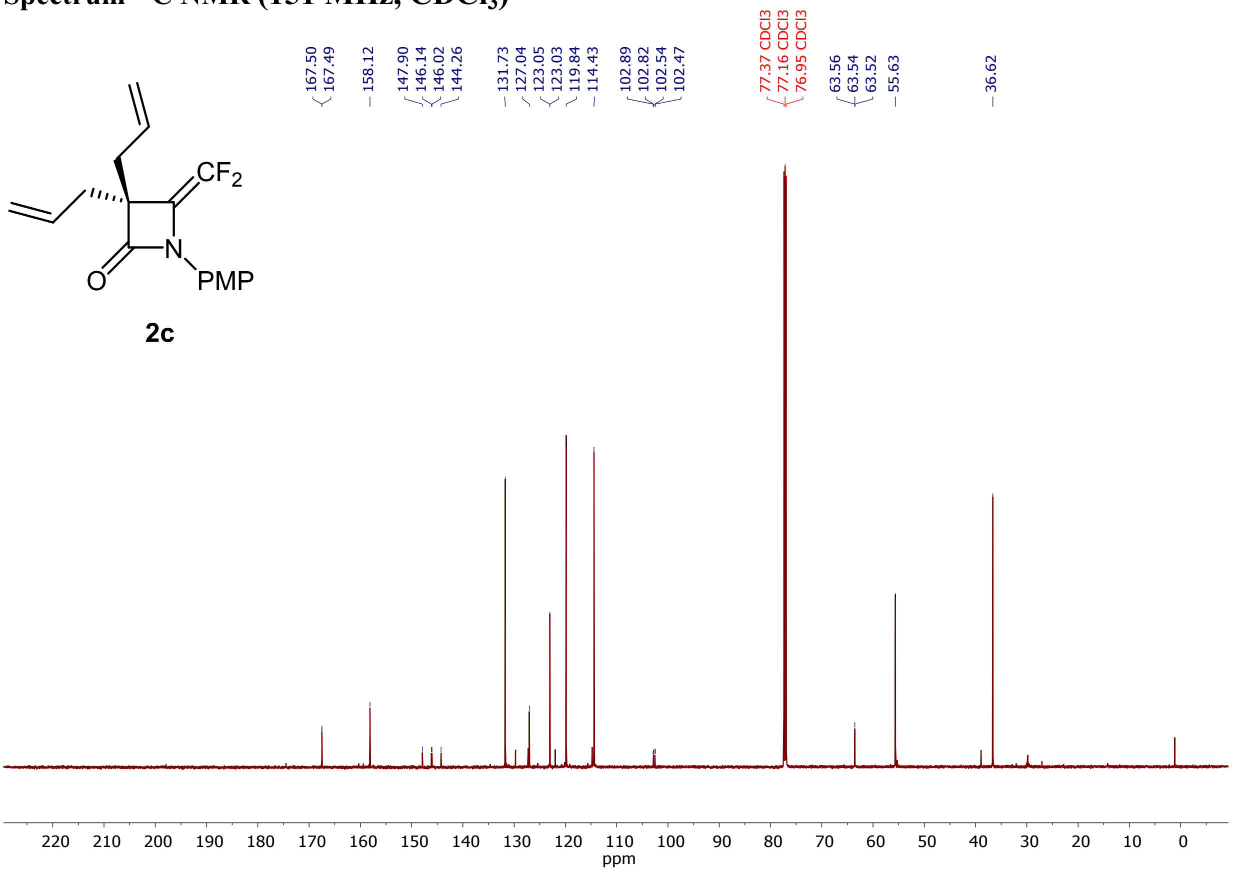

Spectrum ^{19}F NMR (565 MHz, $CDCl_3$)

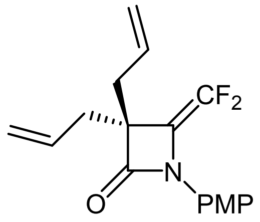

2c

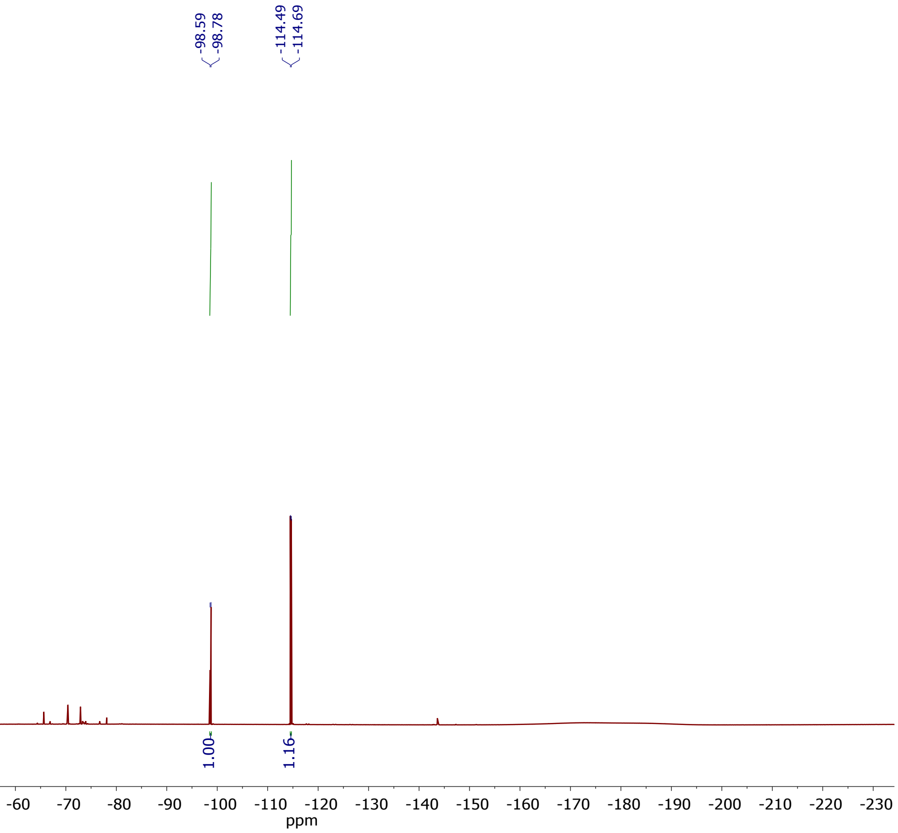

Spectrum ^{1}H NMR (400 MHz, $CDCl_3$)

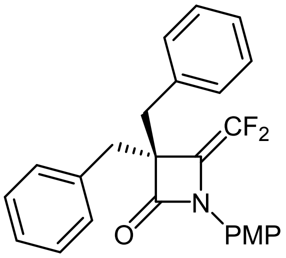

2d

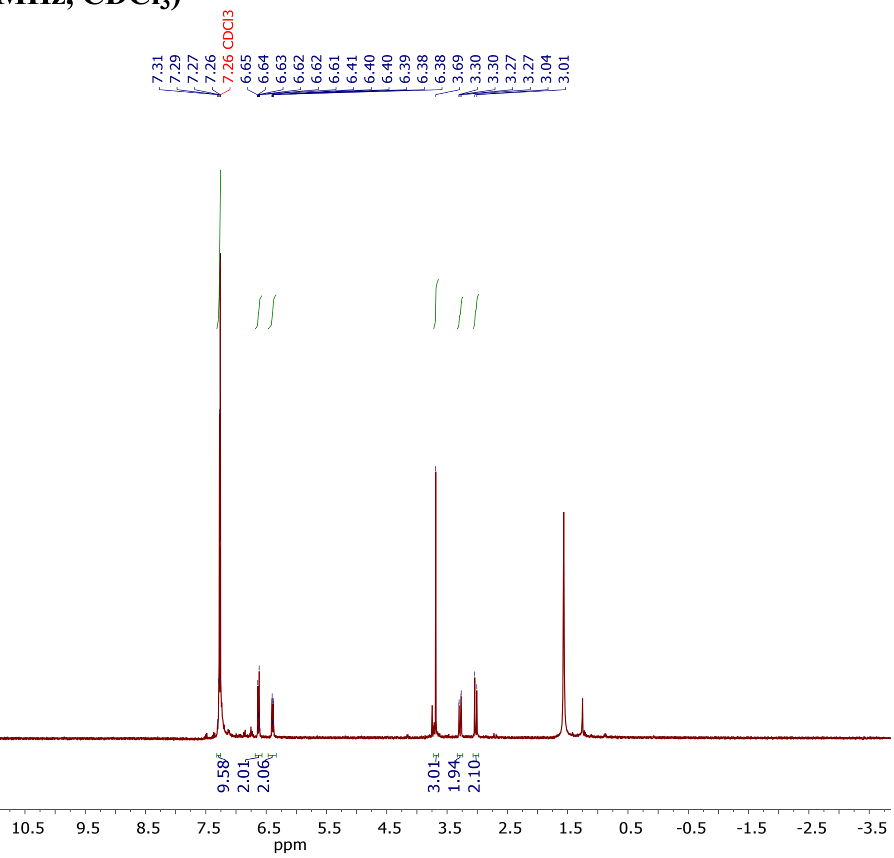

Spectrum ^{13}C NMR (101 MHz, $CDCl_3$)

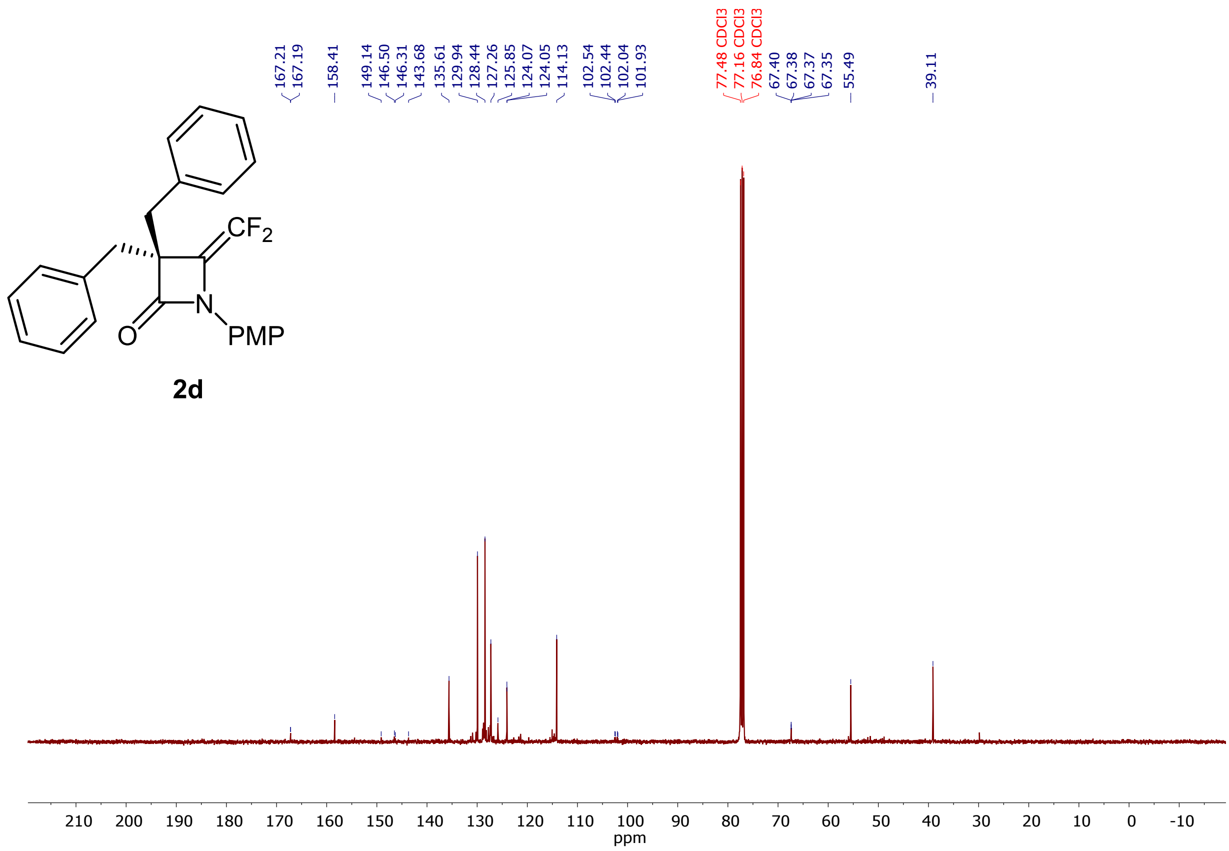

Spectrum ^{19}F NMR (565 MHz, $CDCl_3$)

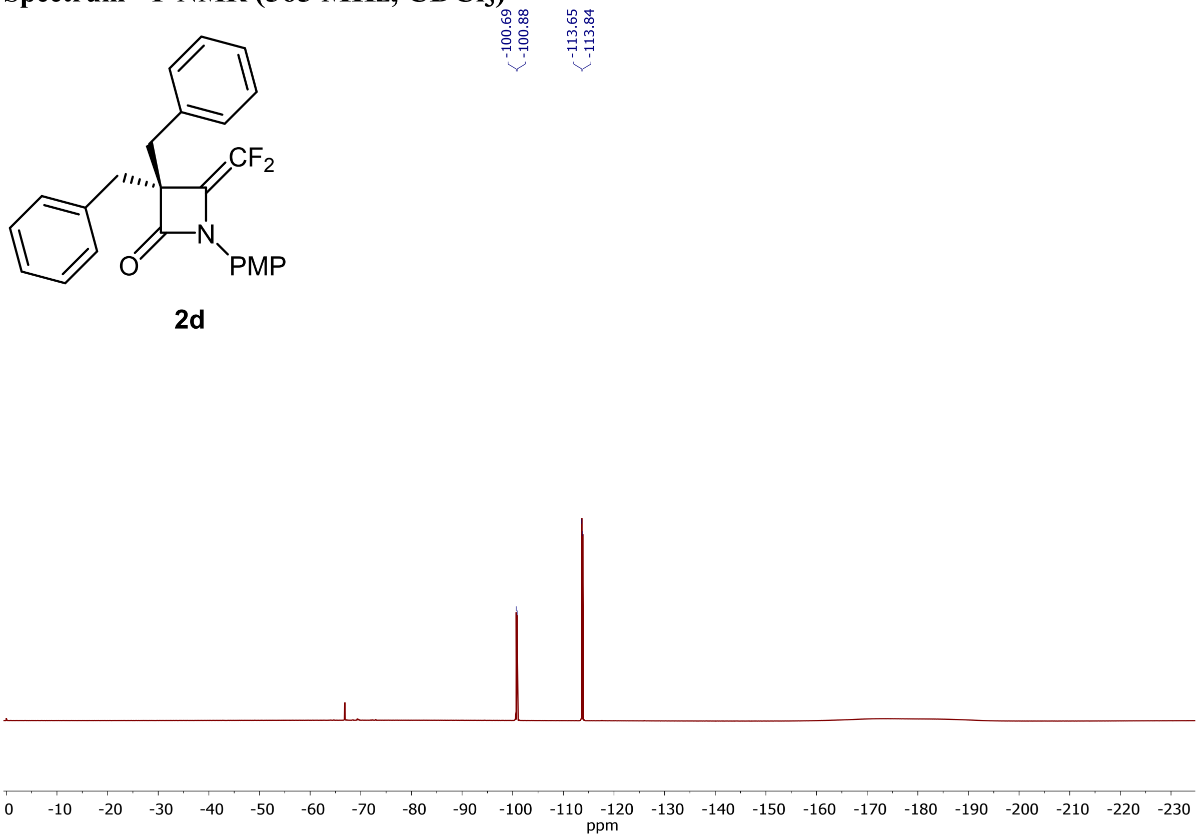

Spectrum ^{1}H NMR (300 MHz, $CDCl_3$)

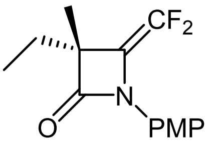

rac **2e**

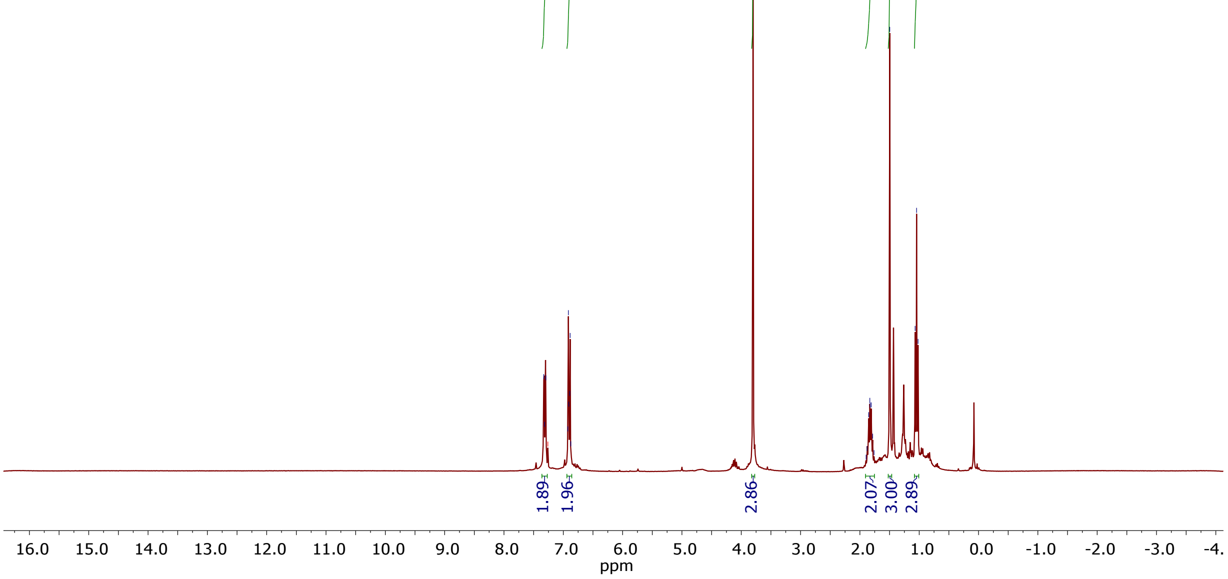

Spectrum ^{13}C NMR (75 MHz, $CDCl_3$)

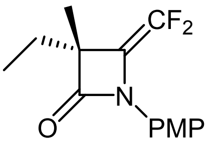

rac **2e**

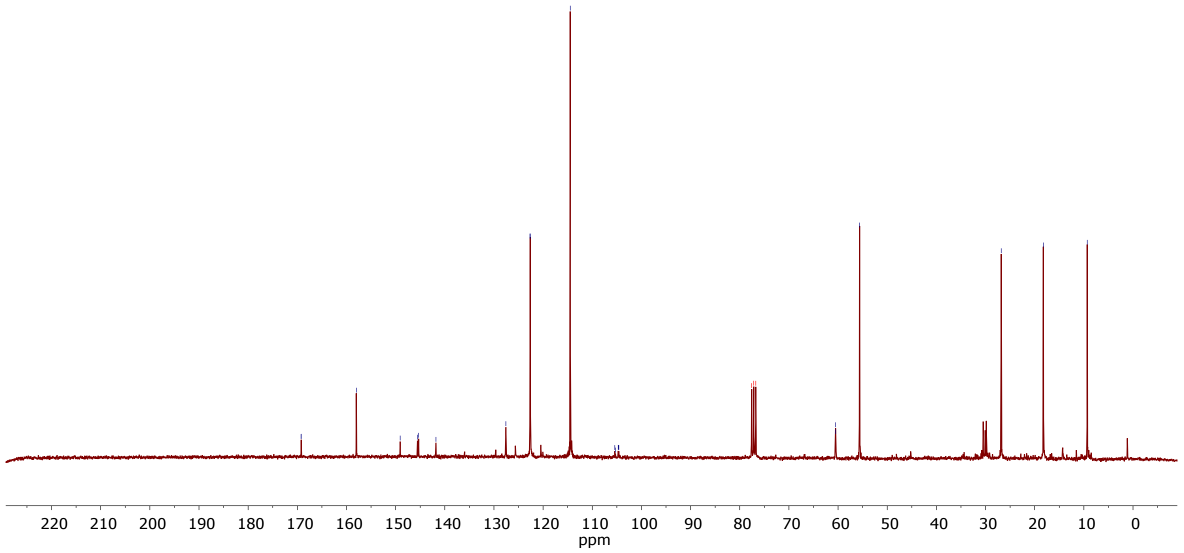

Spectrum ^{19}F NMR (188 MHz, $CDCl_3$)

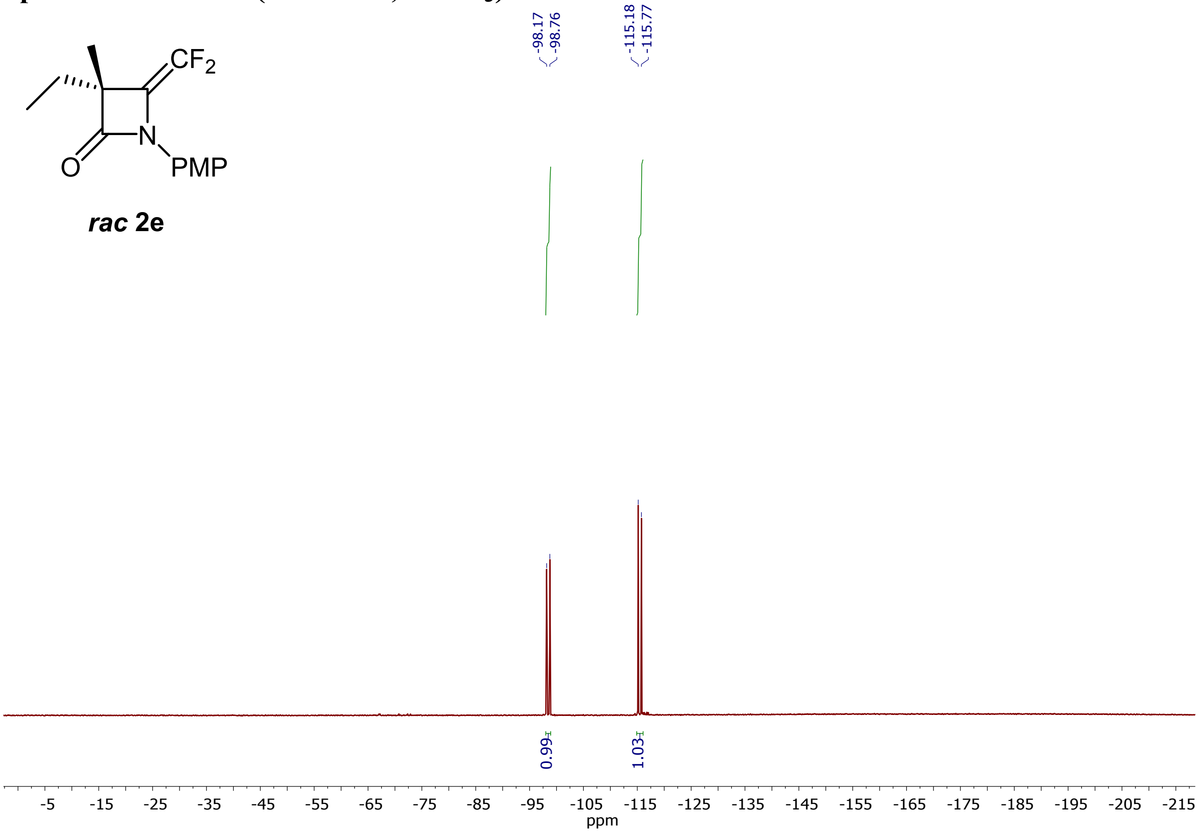

Spectrum ^{1}H NMR (400 MHz, $CDCl_3$)

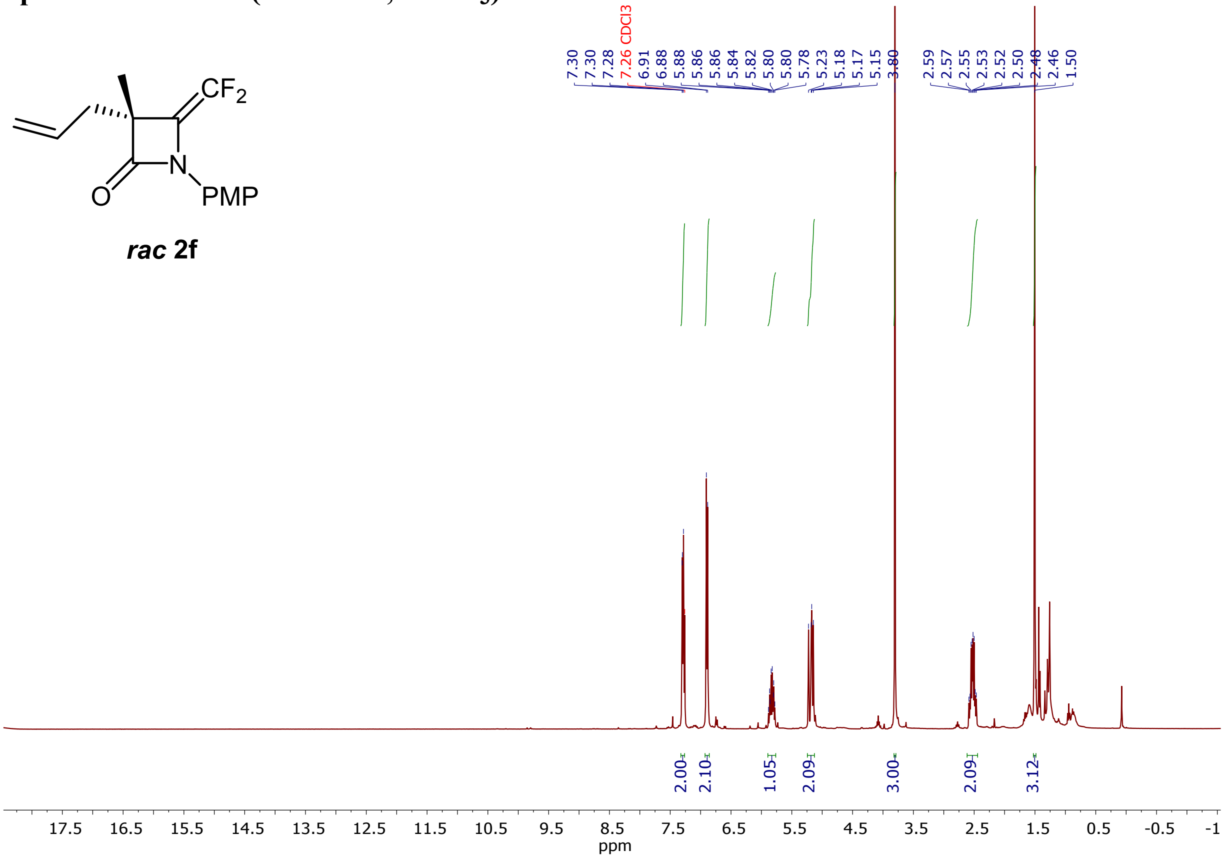

Spectrum ^{13}C NMR (101 MHz, $CDCl_3$)

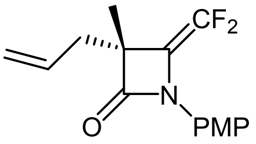

rac **2f**

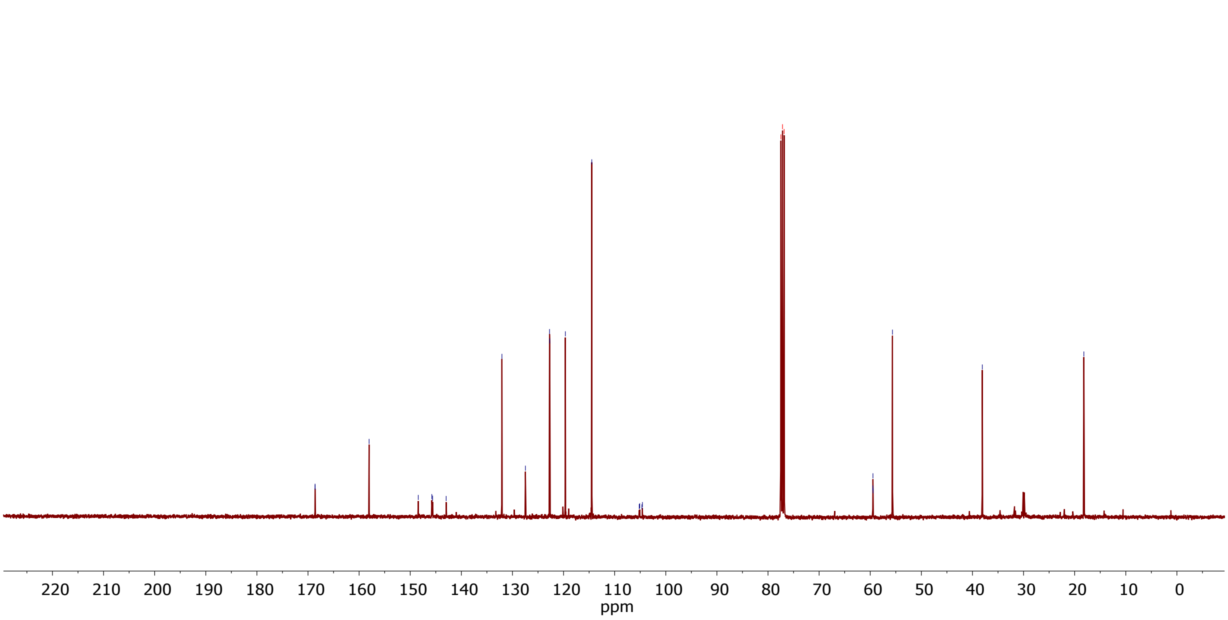

Spectrum ^{19}F NMR (188 MHz, $CDCl_3$)

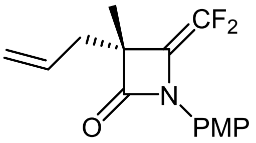

rac **2f**

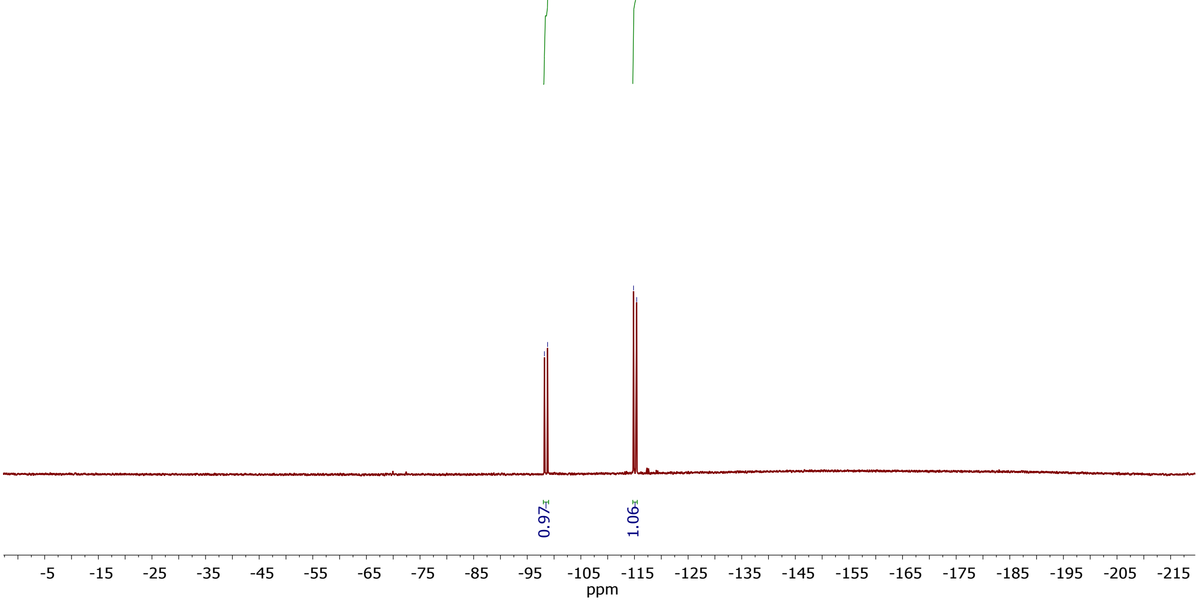

Spectrum ^{1}H NMR (400 MHz, $CDCl_3$)

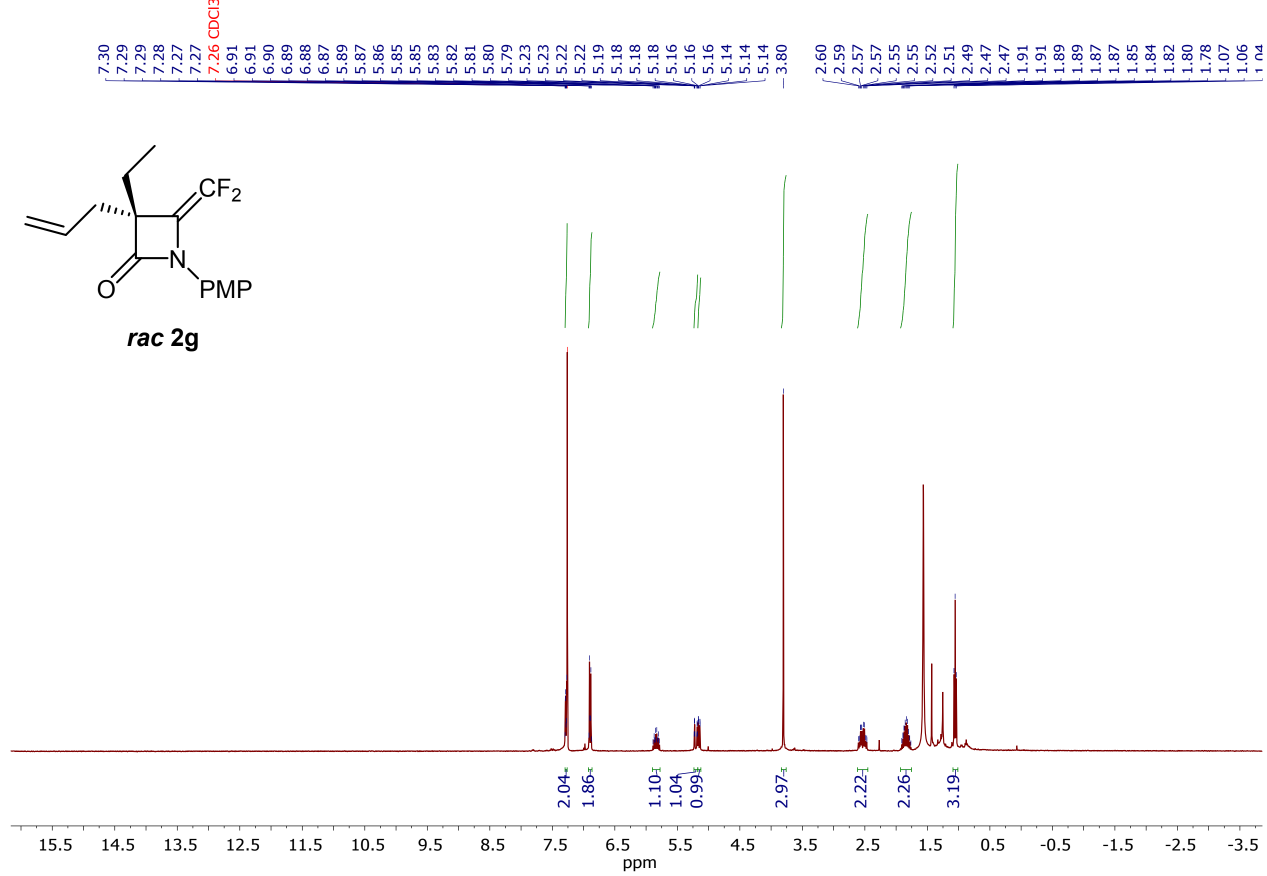

Spectrum ^{13}C NMR (101 MHz, $CDCl_3$)

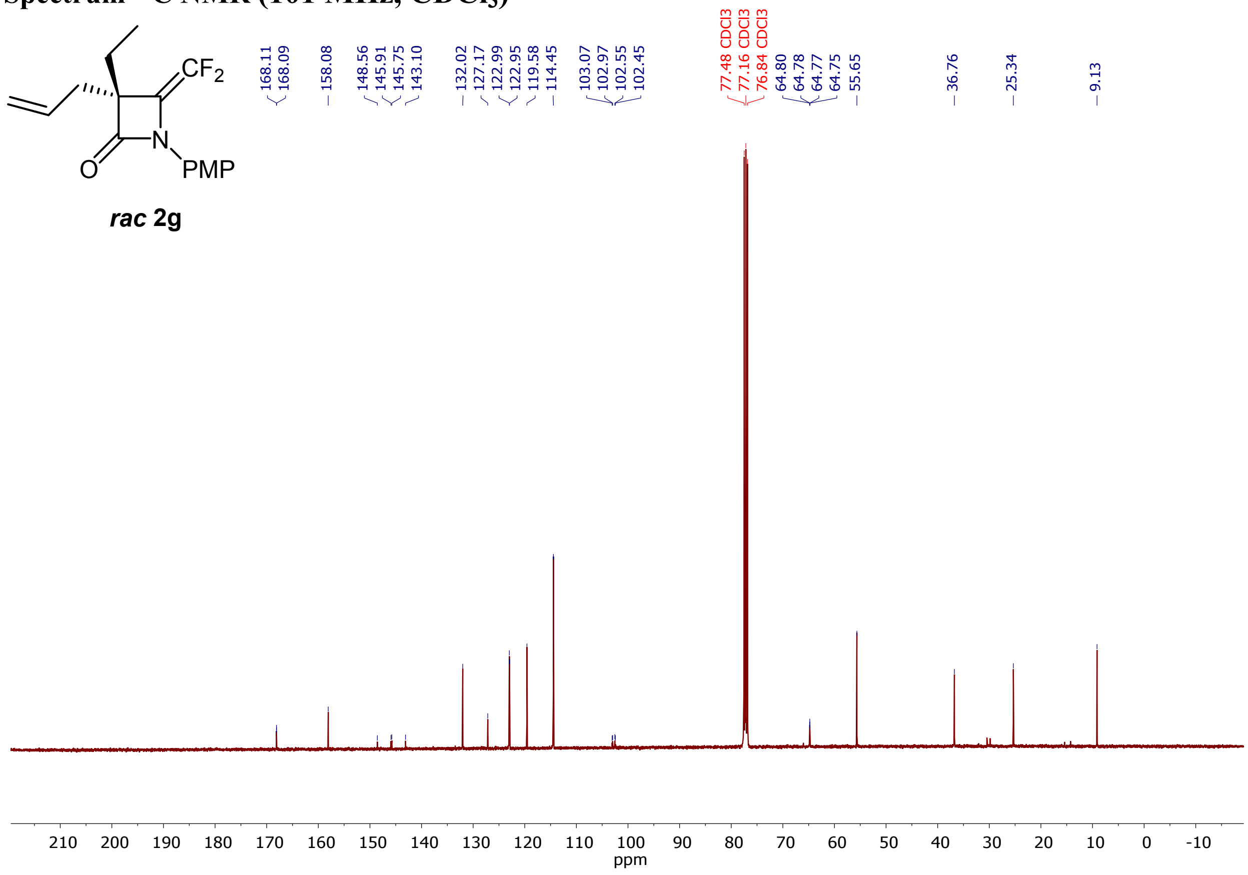

Spectrum ^{19}F NMR (376 MHz, $CDCl_3$)

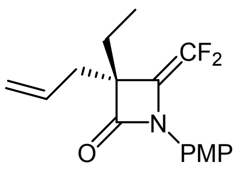

***rac* 2g**

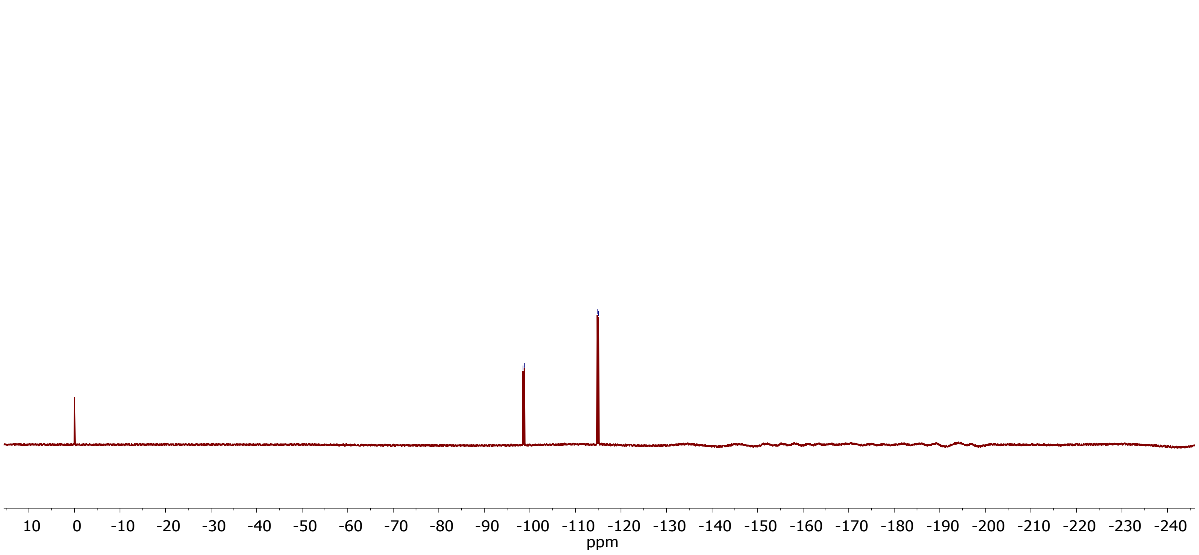

Spectrum ^{1}H NMR (300 MHz, $CDCl_3$)

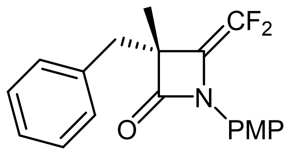

***rac* 2h**

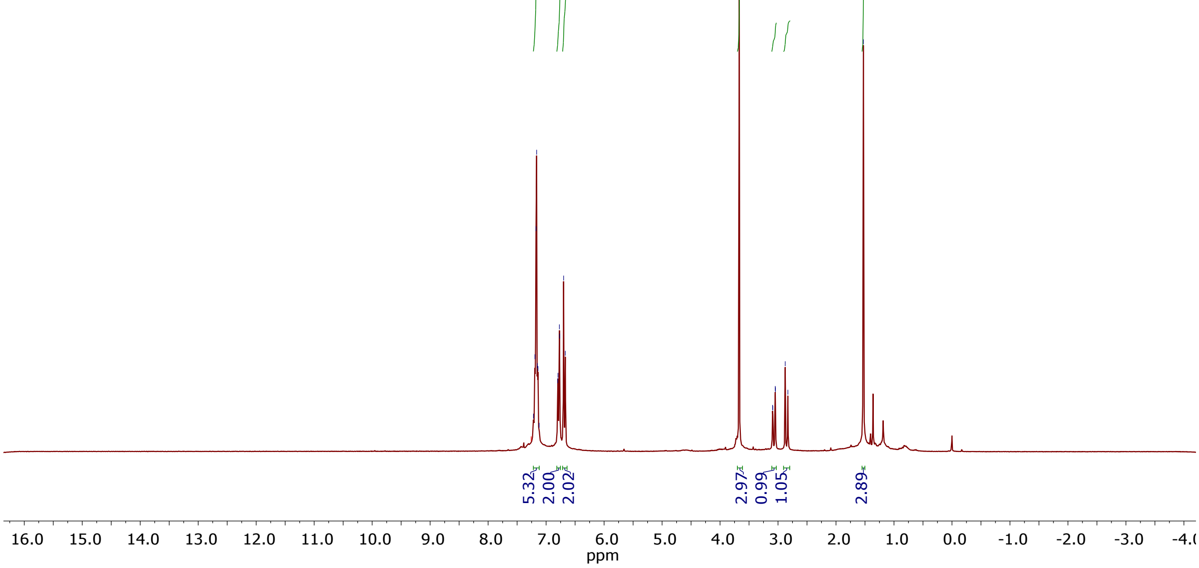

Spectrum ^{13}C NMR (75 MHz, $CDCl_3$)

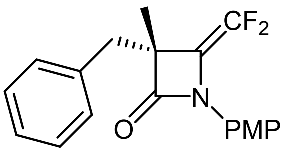

***rac* 2h**

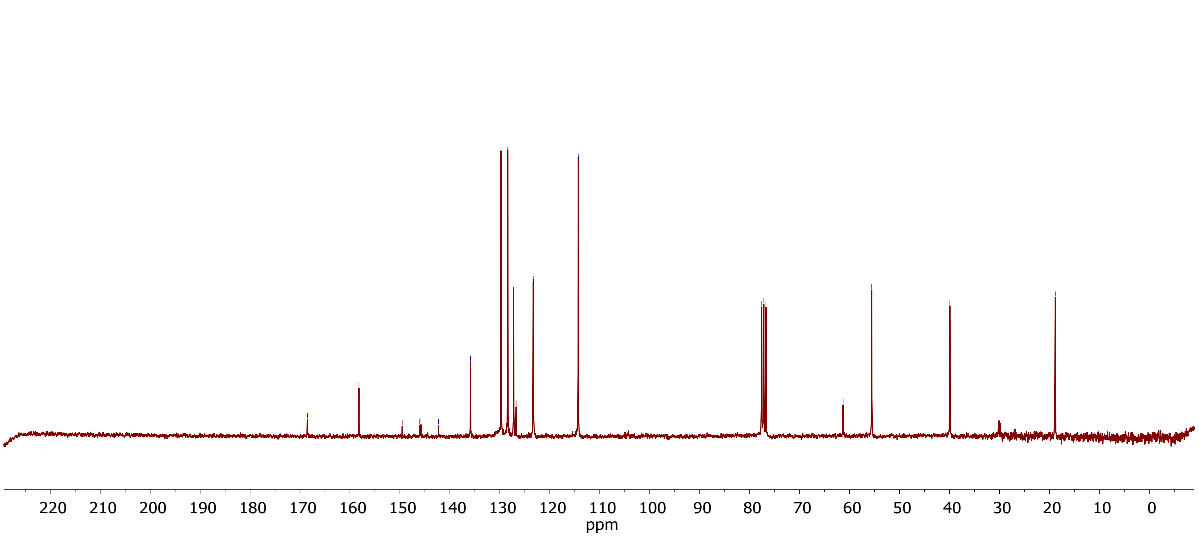

Spectrum ^{19}F NMR (188 MHz, $CDCl_3$)

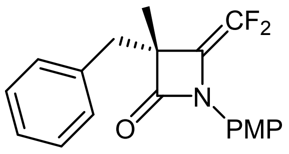

***rac* 2h**

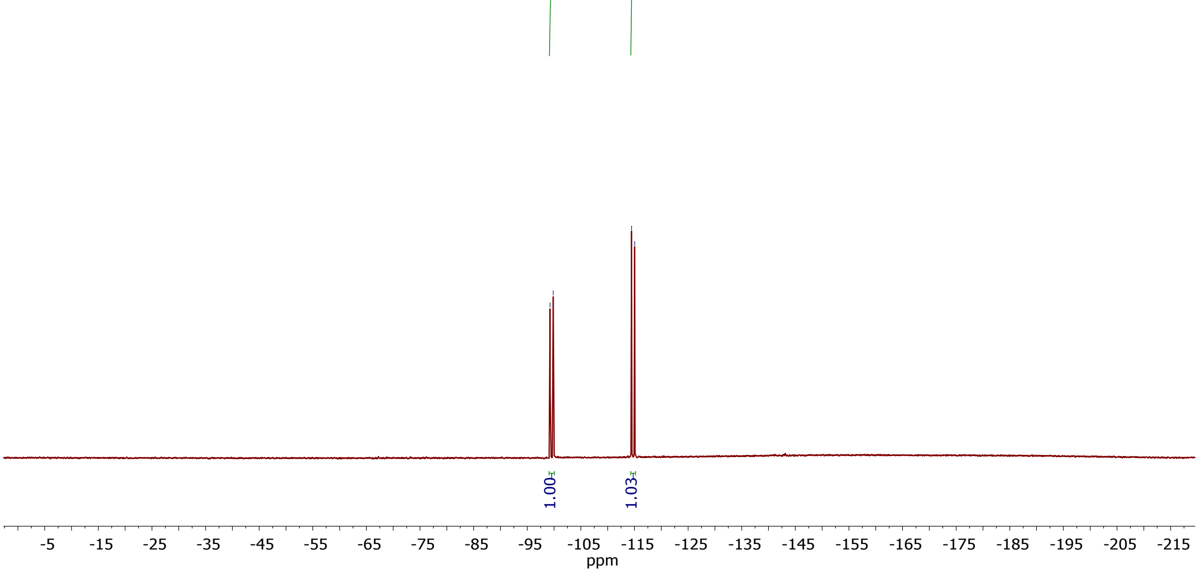

Spectrum ^{1}H NMR (600 MHz, $CDCl_3$)

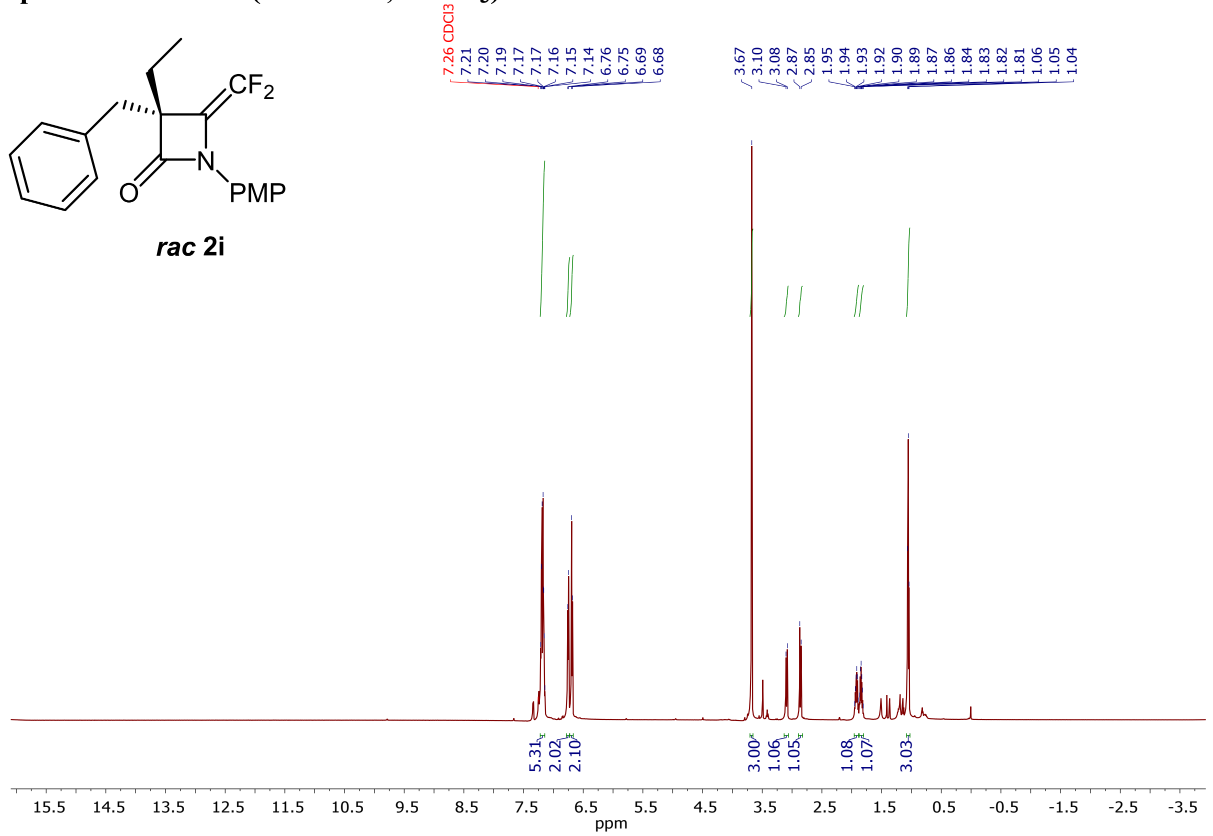

Spectrum ^{13}C NMR (151 MHz, $CDCl_3$)

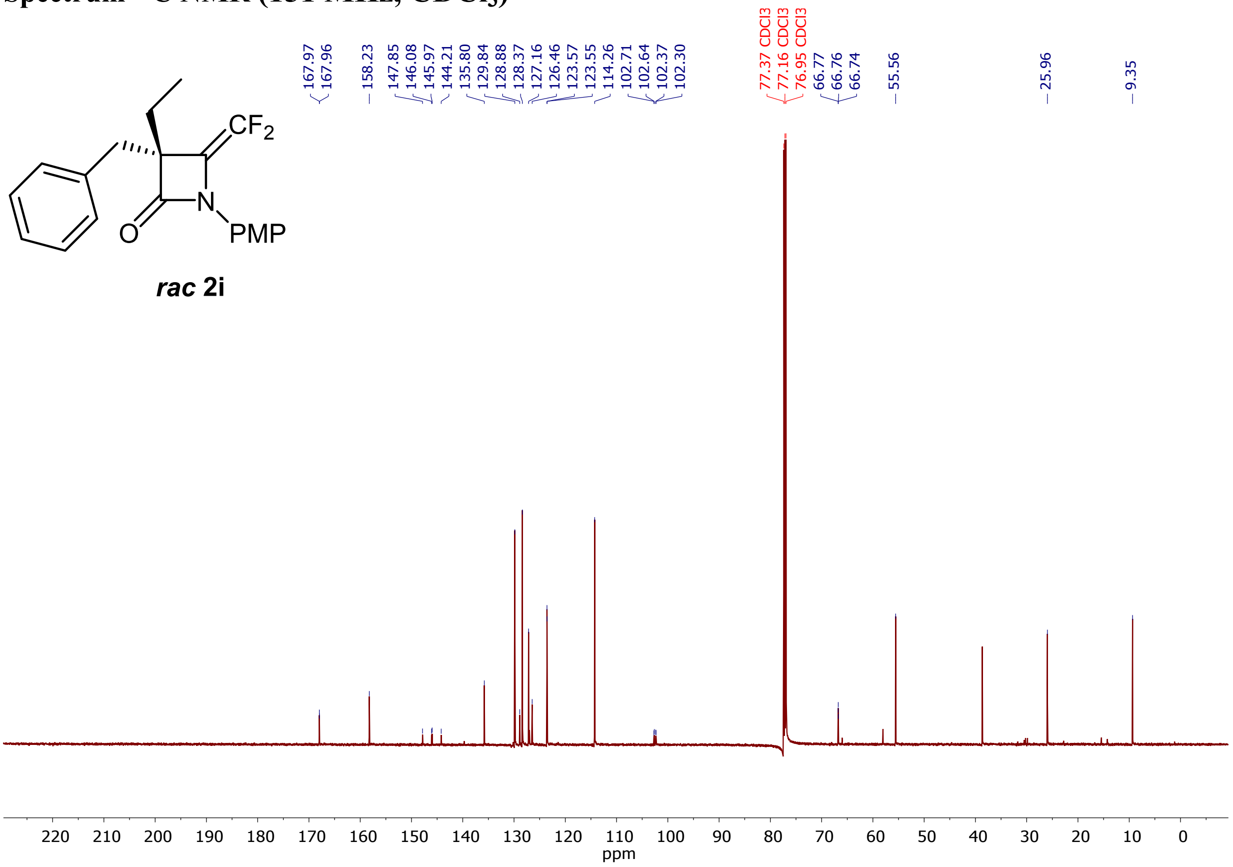

Spectrum ^{19}F NMR (565 MHz, $CDCl_3$)

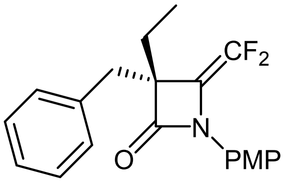

***rac* 2i**

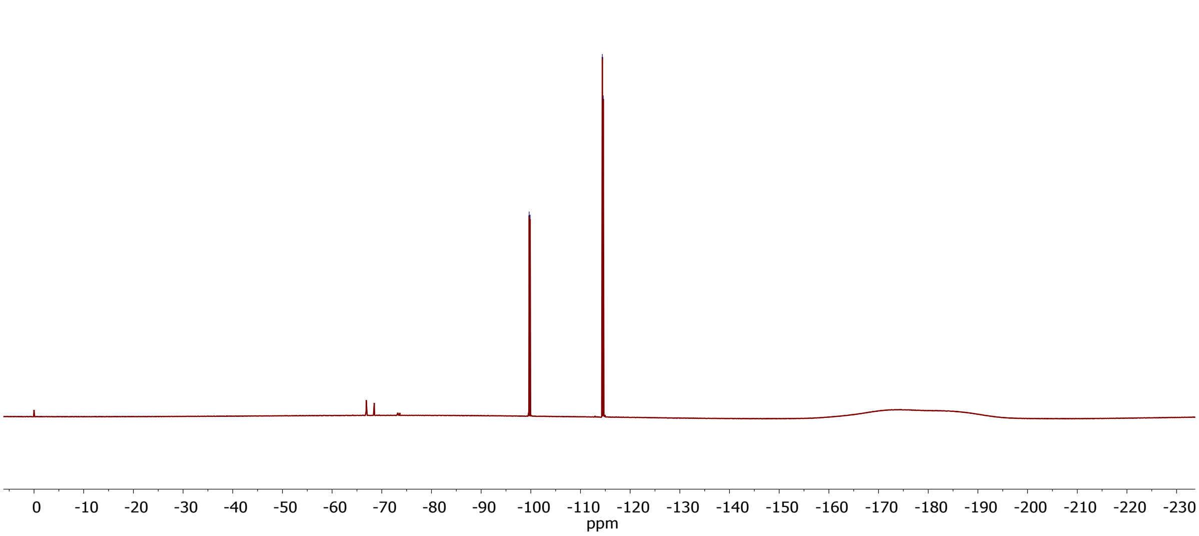

Spectrum ^{1}H NMR (400 MHz, $CDCl_3$)

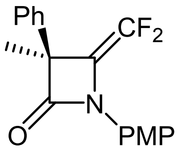

***rac* 2j**

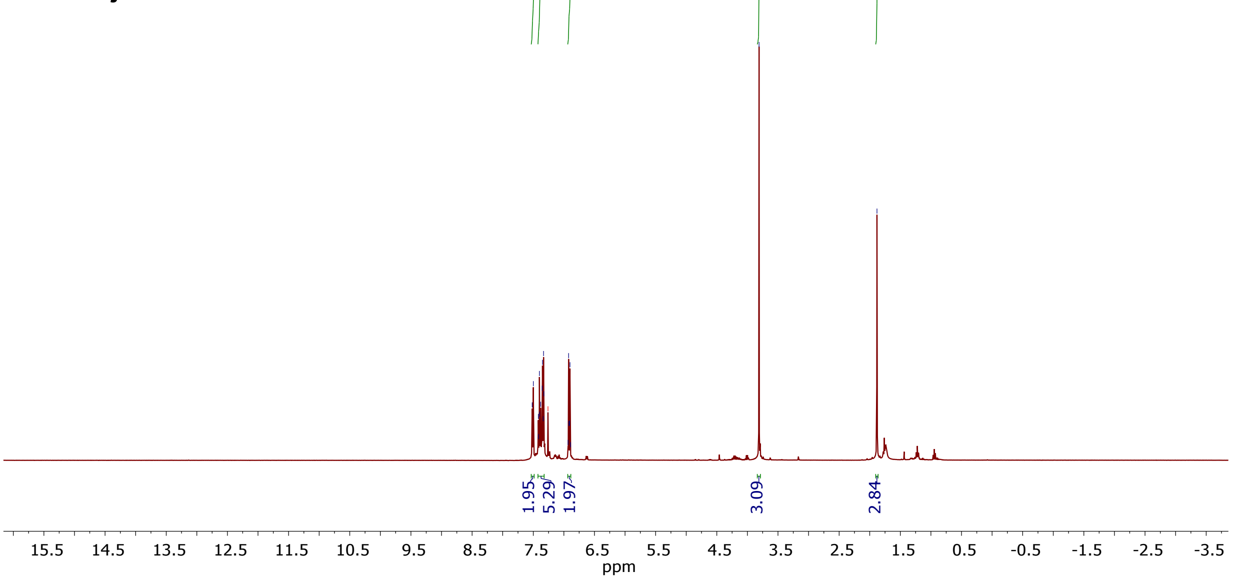

Spectrum ^{13}C NMR (101 MHz, $CDCl_3$)

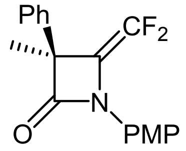

***rac* 2j**

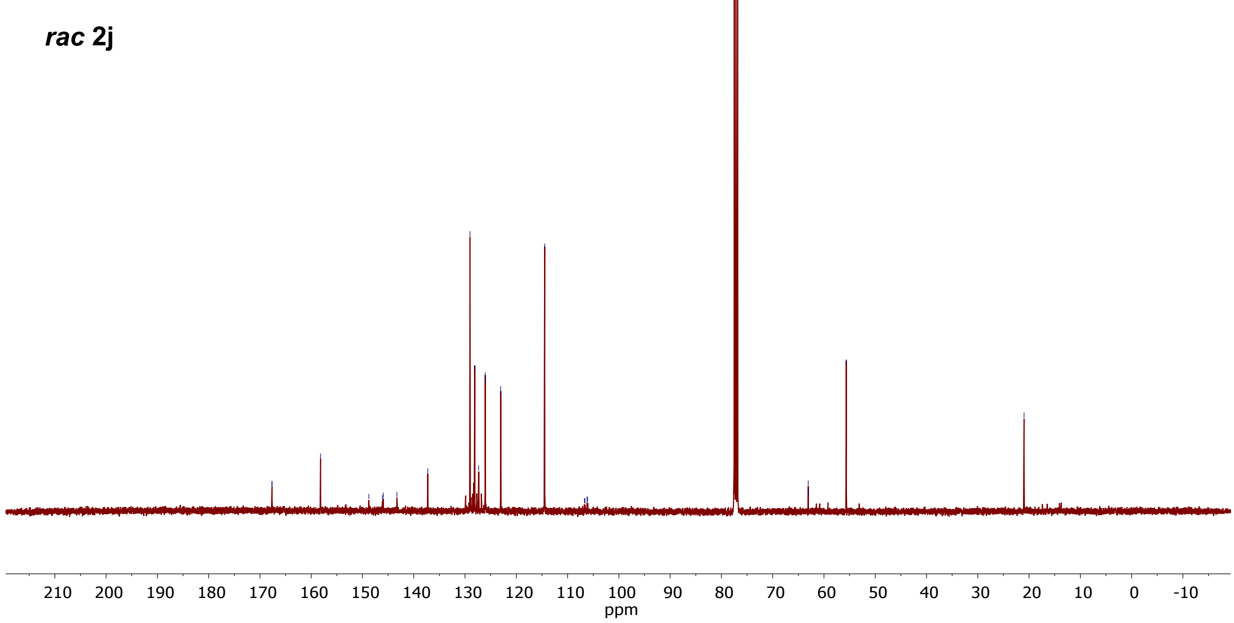

Spectrum ^{19}F NMR (376 MHz, $CDCl_3$)

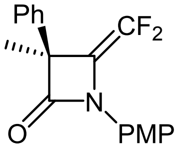

***rac* 2j**

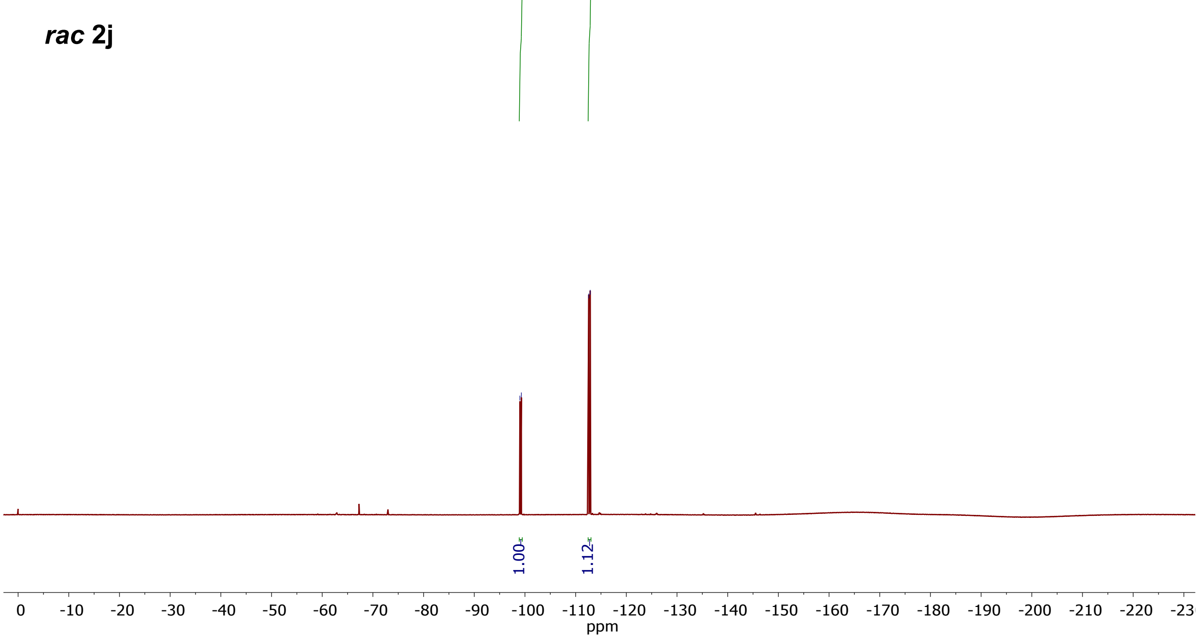

Spectrum ^{1}H NMR (400 MHz, $CDCl_3$)

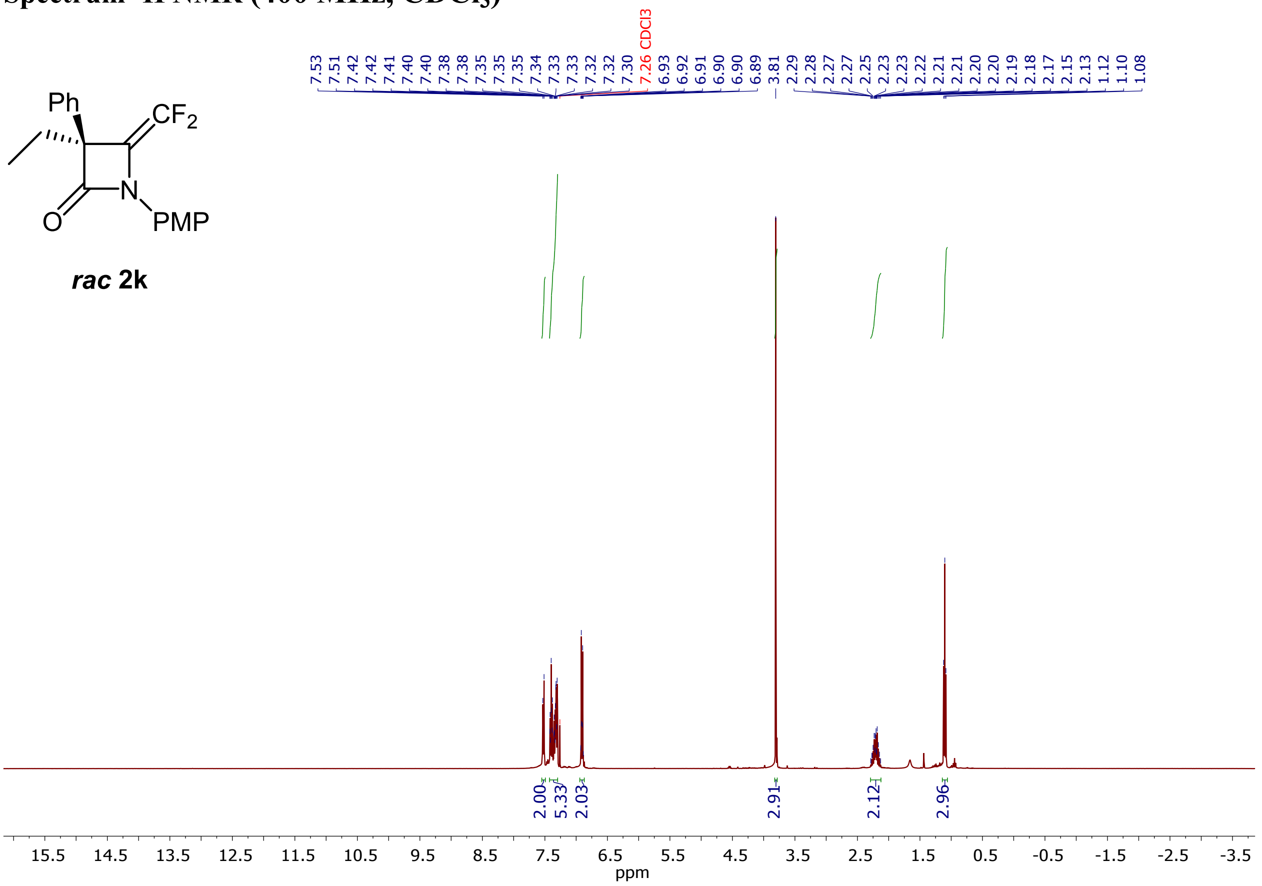

Spectrum ^{13}C NMR (101 MHz, $CDCl_3$)

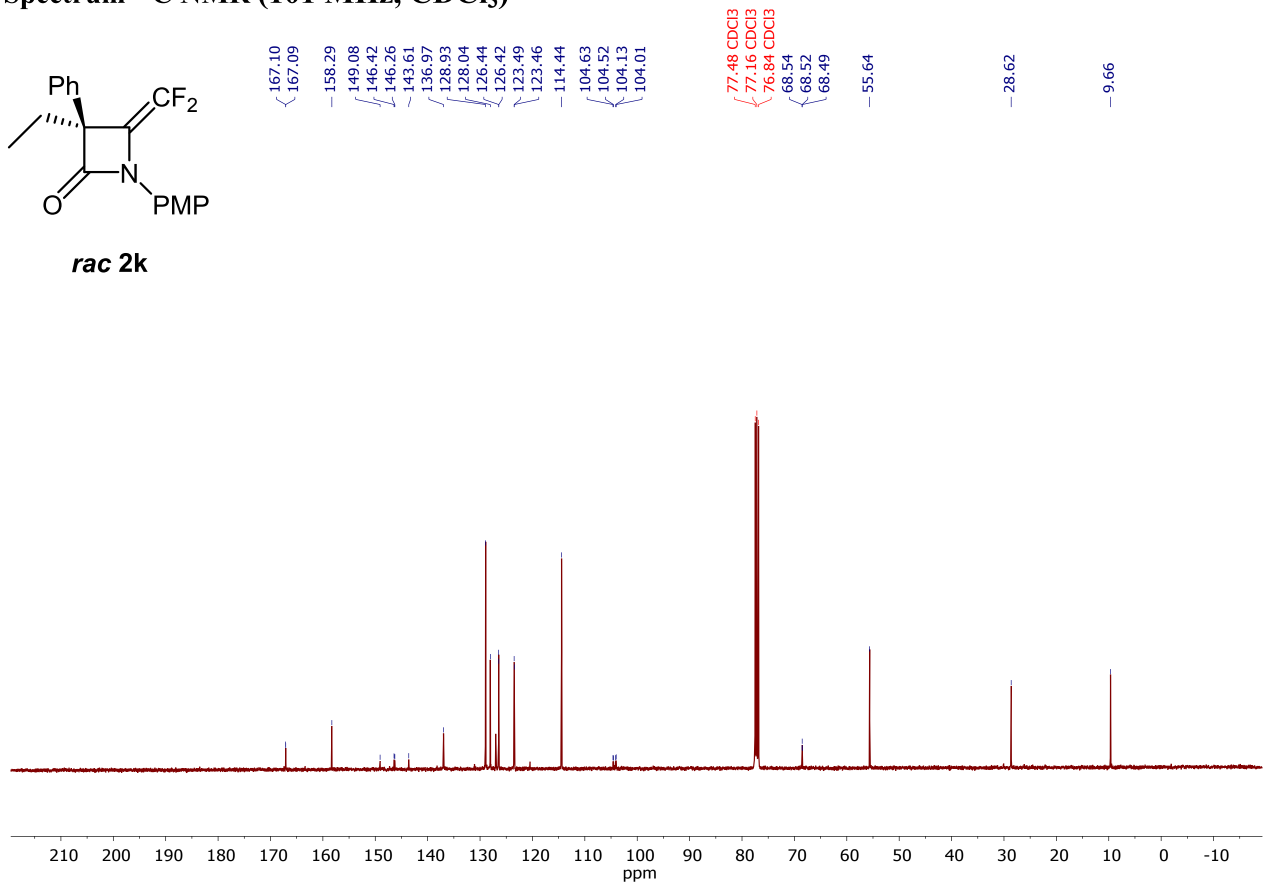

Spectrum ^{19}F NMR (376 MHz, $CDCl_3$)

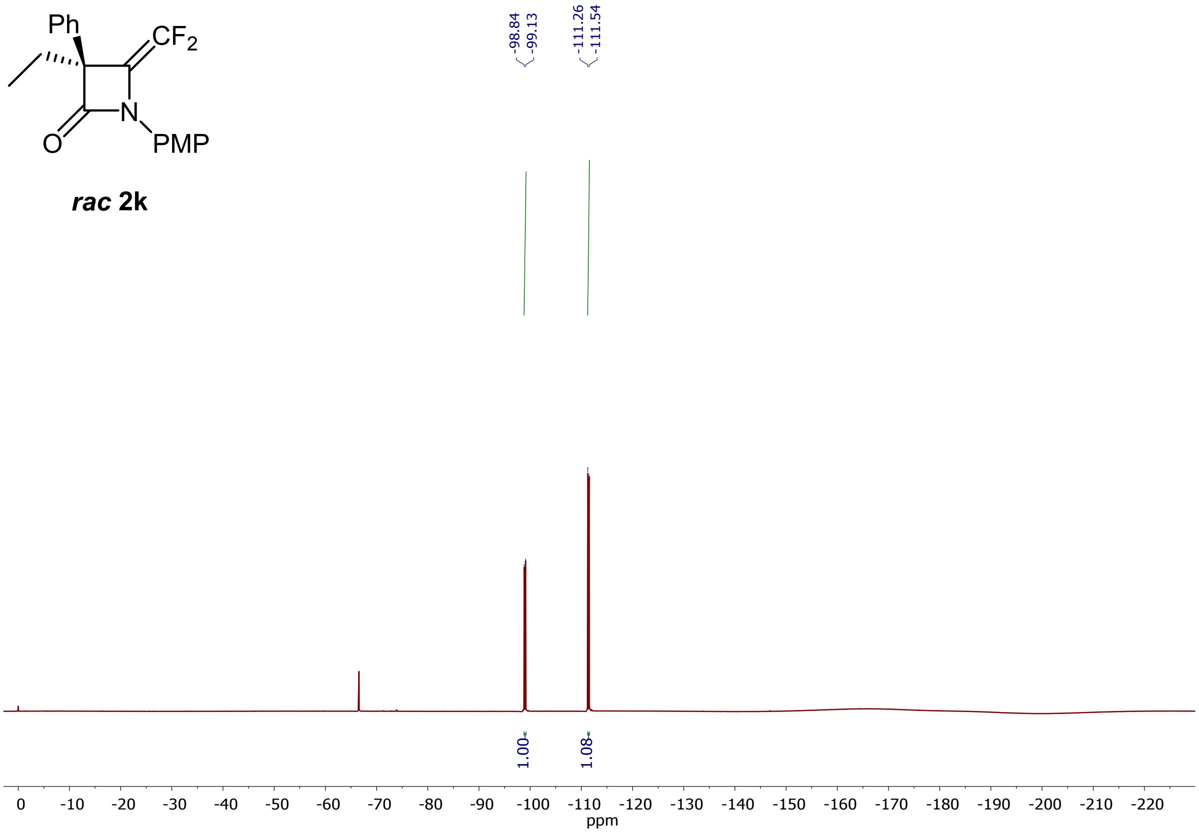

rac **2k**

Spectrum ^{1}H NMR (400 MHz, $CDCl_3$)

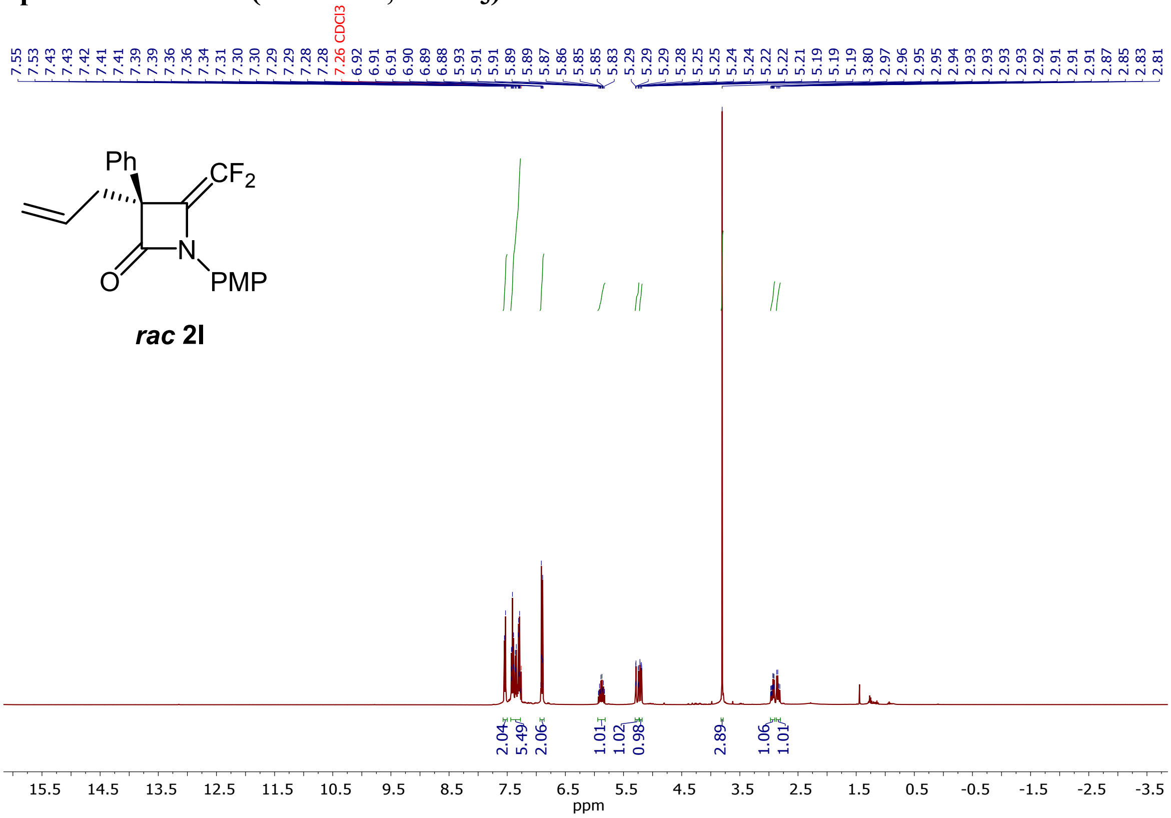

rac **2l**

Spectrum ^{13}C NMR (101 MHz, CDCl$_3$)

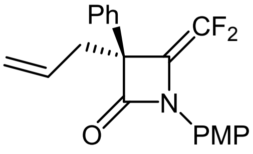

rac **2l**

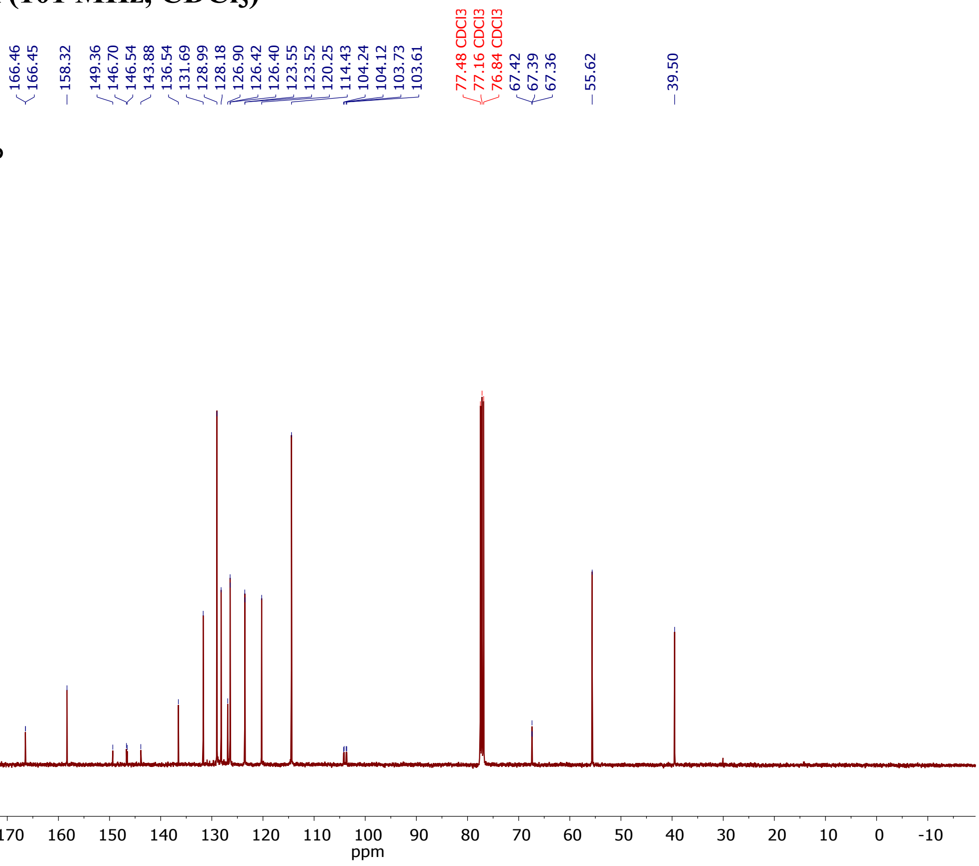

Spectrum ^{19}F NMR (376 MHz, CDCl$_3$)

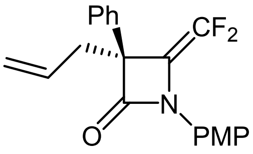

rac **2l**

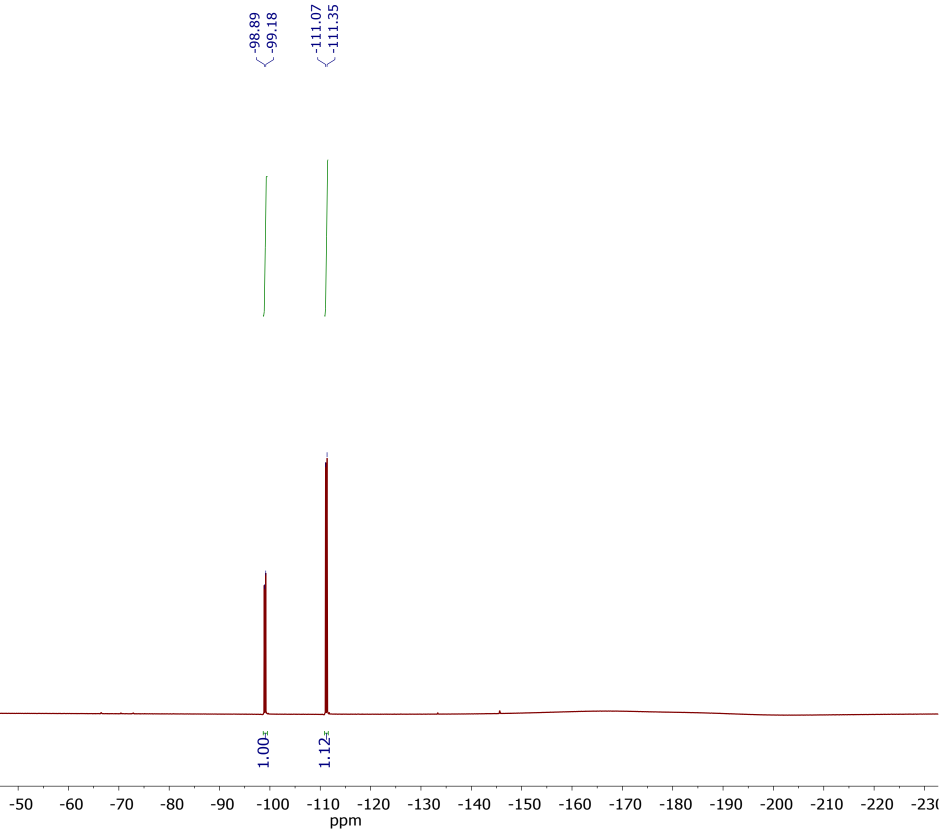

Spectrum ^{1}H NMR (600 MHz, $CDCl_3$)

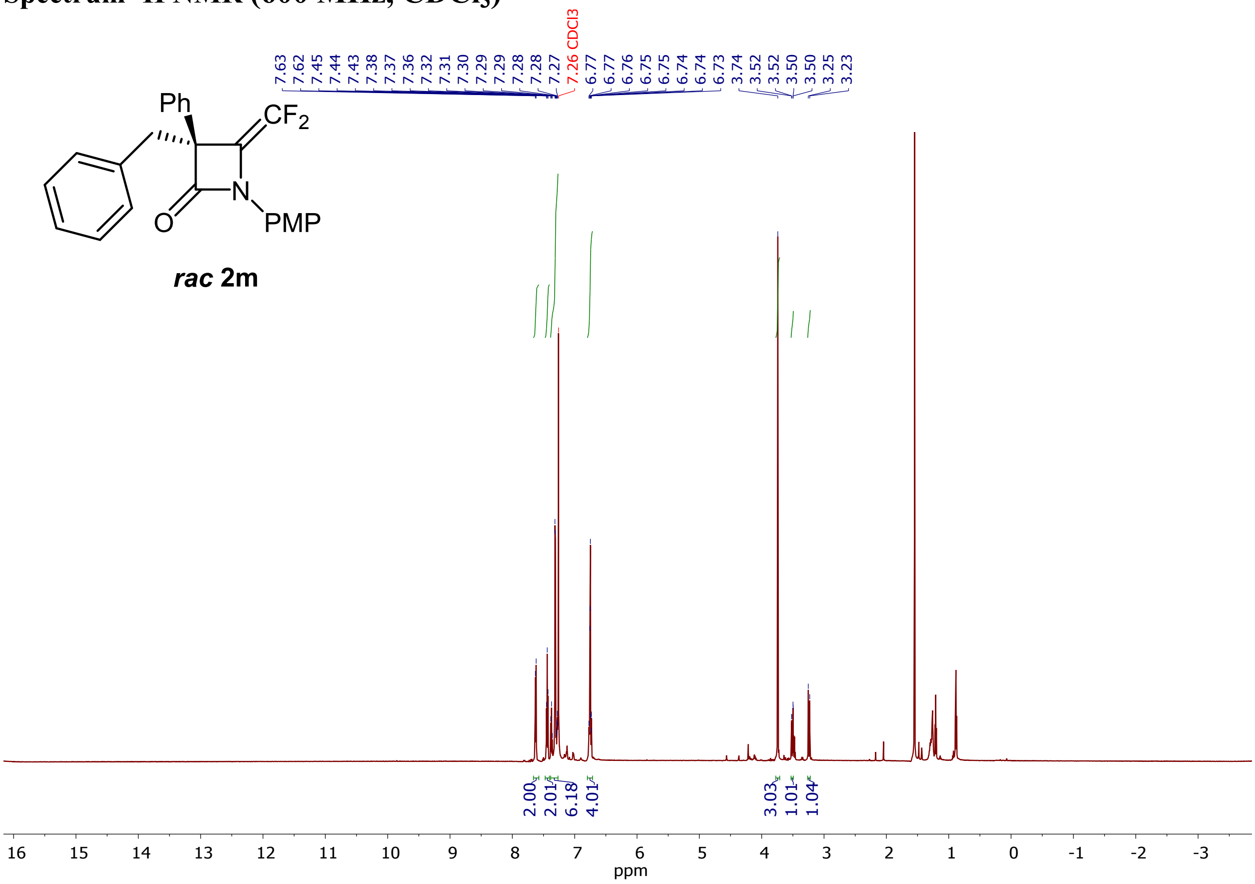

Spectrum ^{13}C NMR (151 MHz, $CDCl_3$)

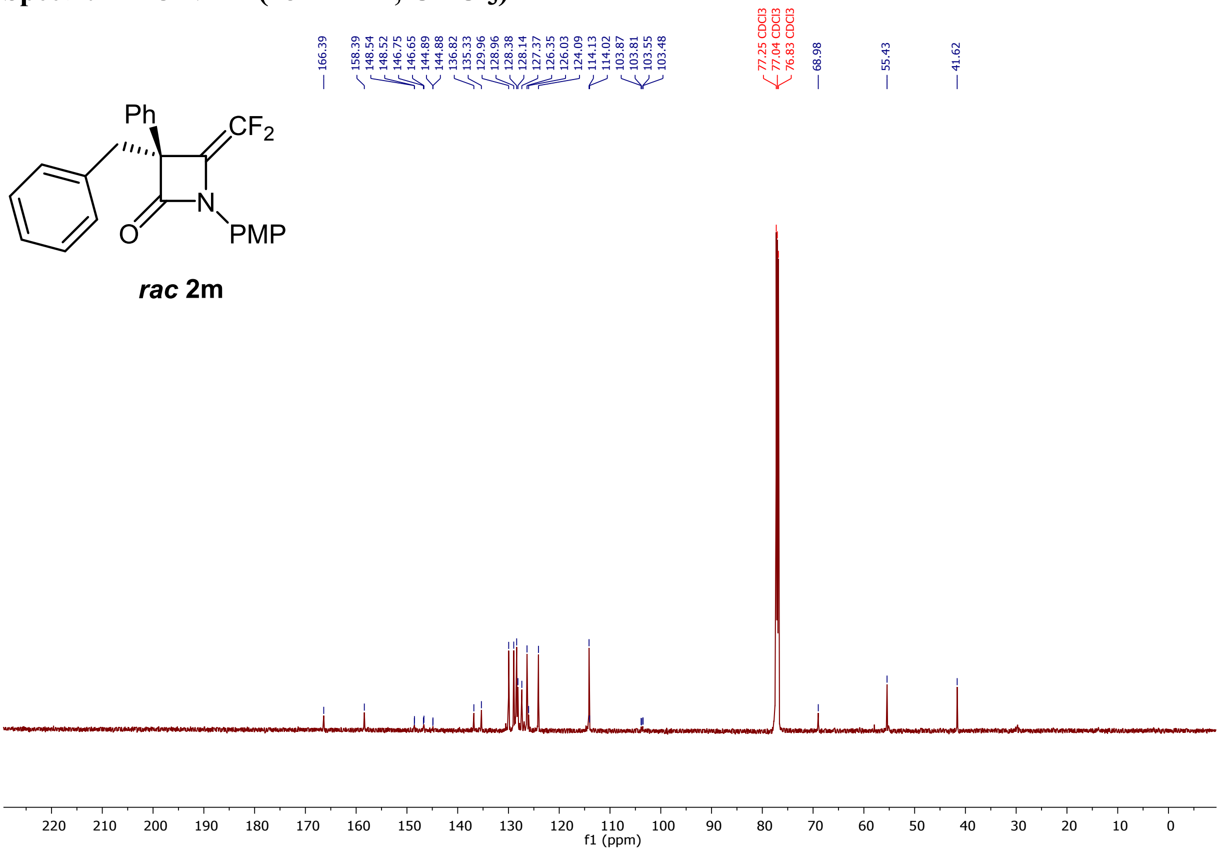

Spectrum ^{19}F NMR (565 MHz, $CDCl_3$)

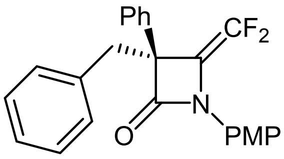

***rac* 2m**

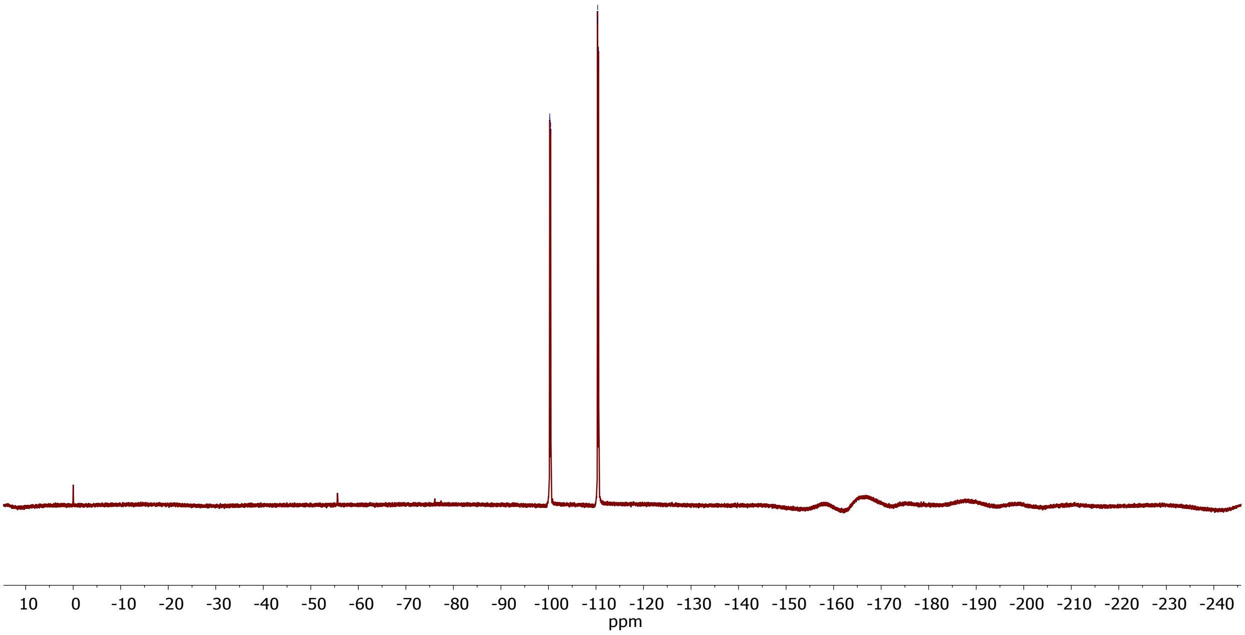

Spectrum ^{1}H NMR (400 MHz, $CDCl_3$)

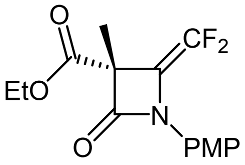

***rac* 2o**

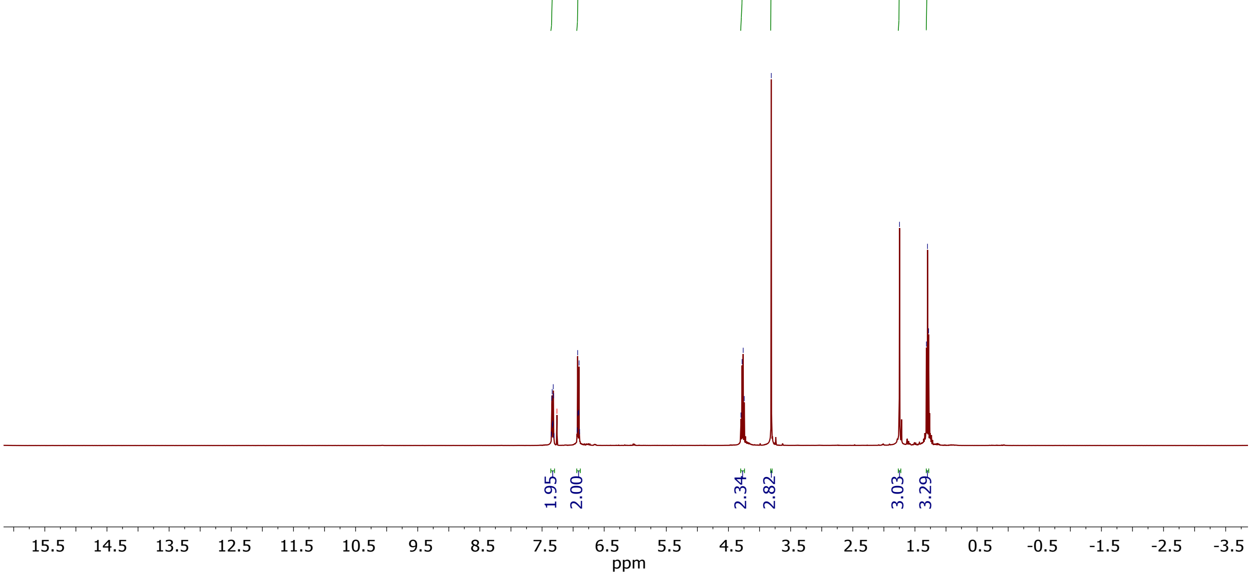

Spectrum ^{13}C NMR (101 MHz, $CDCl_3$)

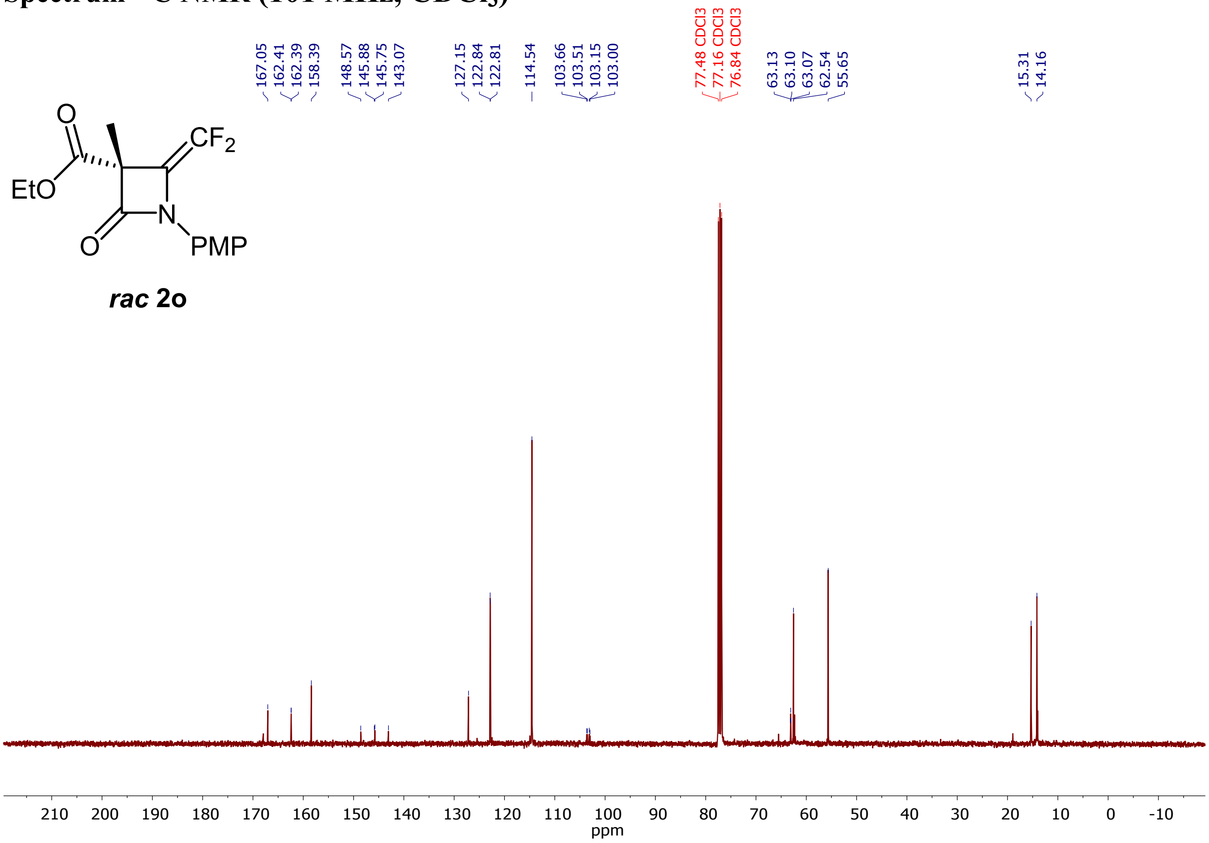

Spectrum ^{19}F NMR (376 MHz, $CDCl_3$)

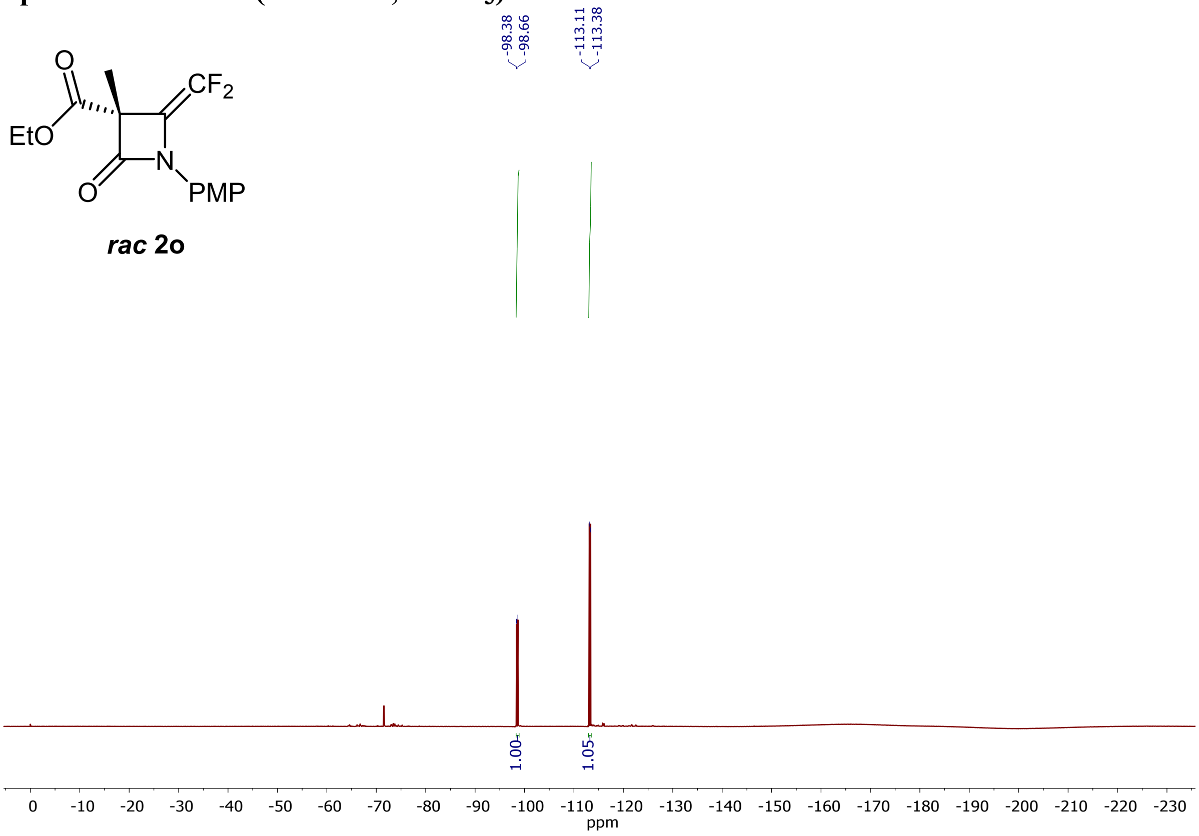

Spectrum ^{1}H NMR (600 MHz, $CDCl_3$)

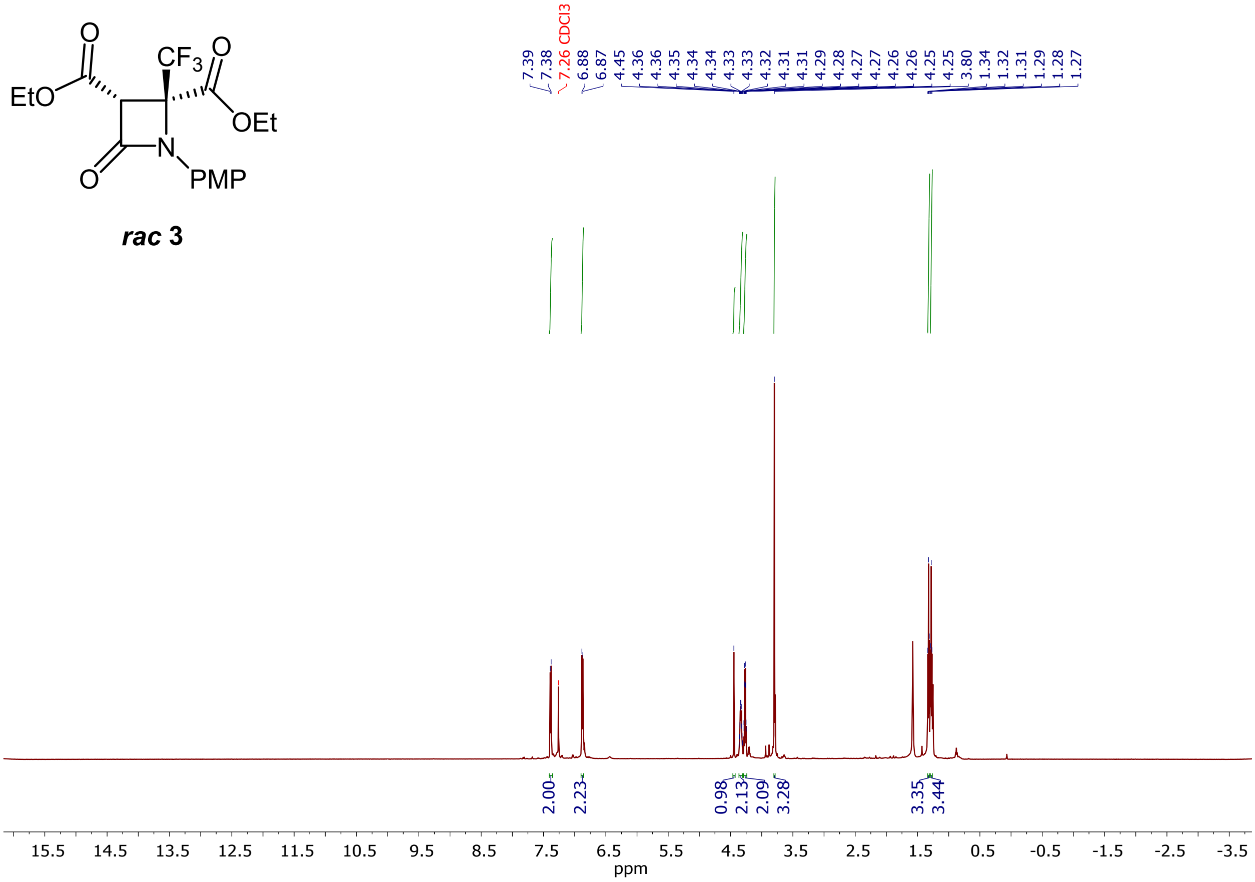

***rac* 3**

Spectrum ^{13}C NMR (151 MHz, $CDCl_3$)

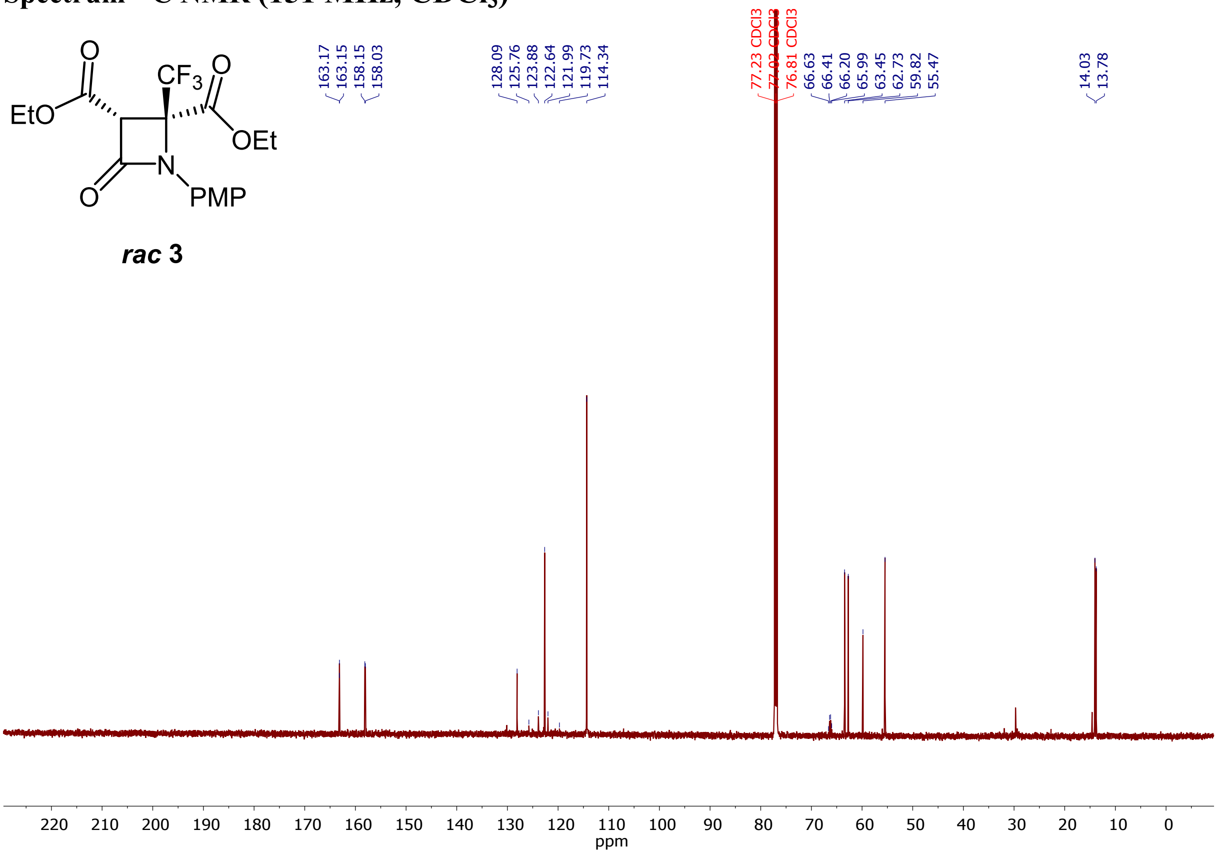

***rac* 3**

Spectrum ^{19}F NMR (565 MHz, $CDCl_3$)

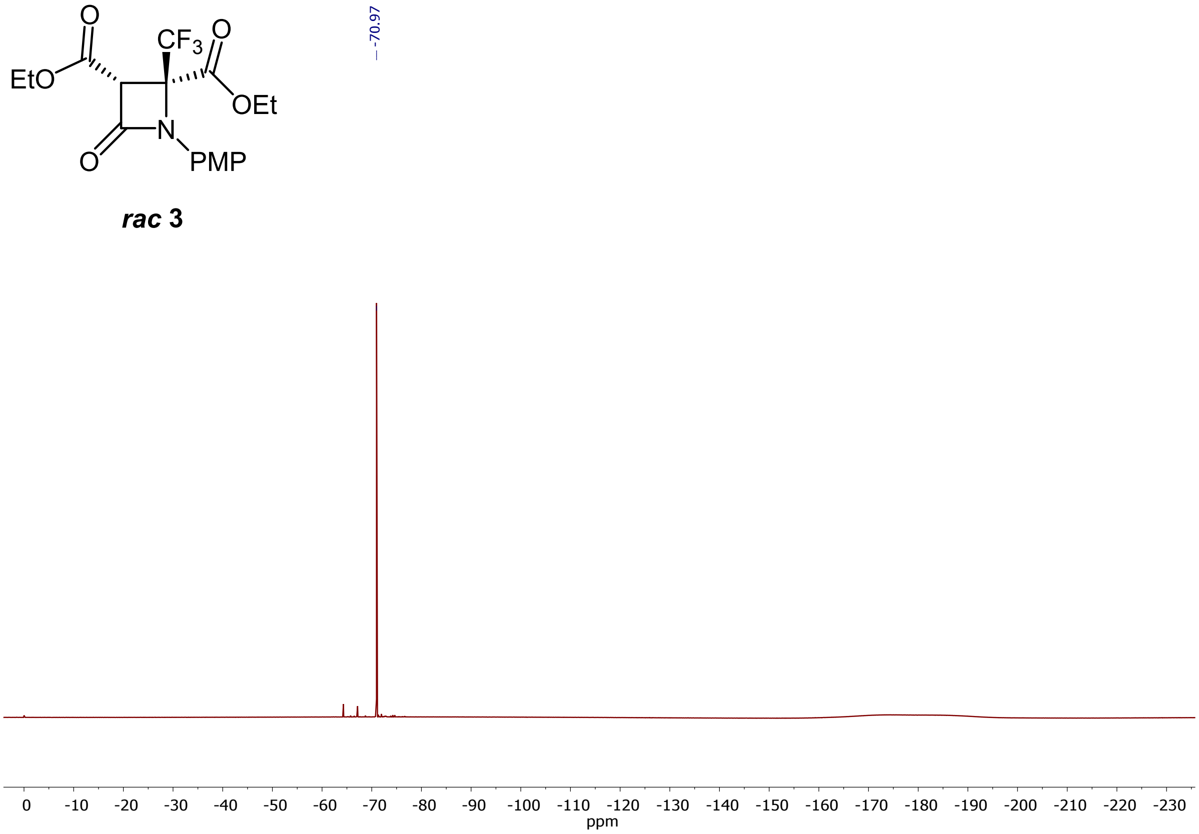

Spectrum ^{1}H-^{1}H NMR 2D COSY, (600 MHz, $CDCl_3$)

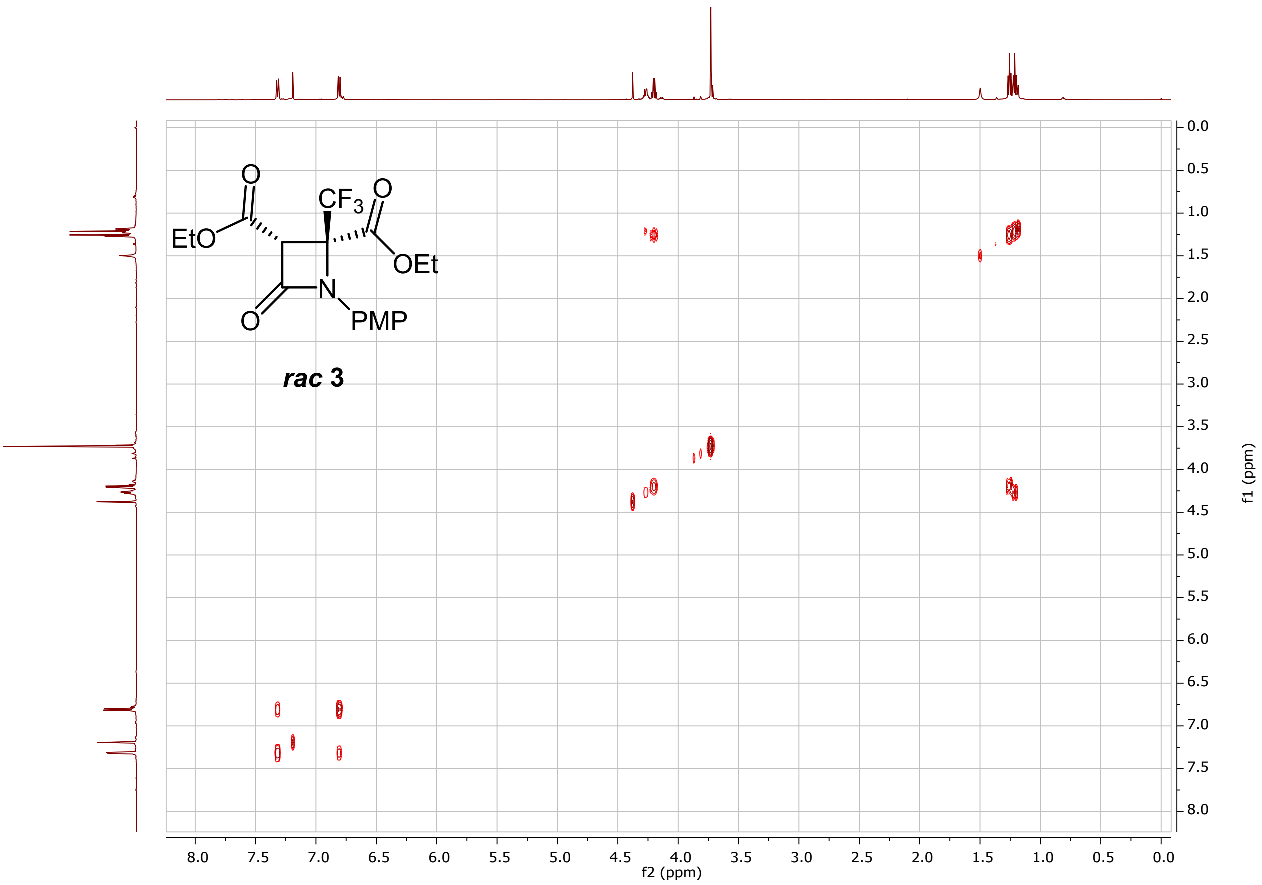

Spectrum ¹H (600 MHz)-¹³C (151 MHz) NMR 2D HSQC, $CDCl_3$

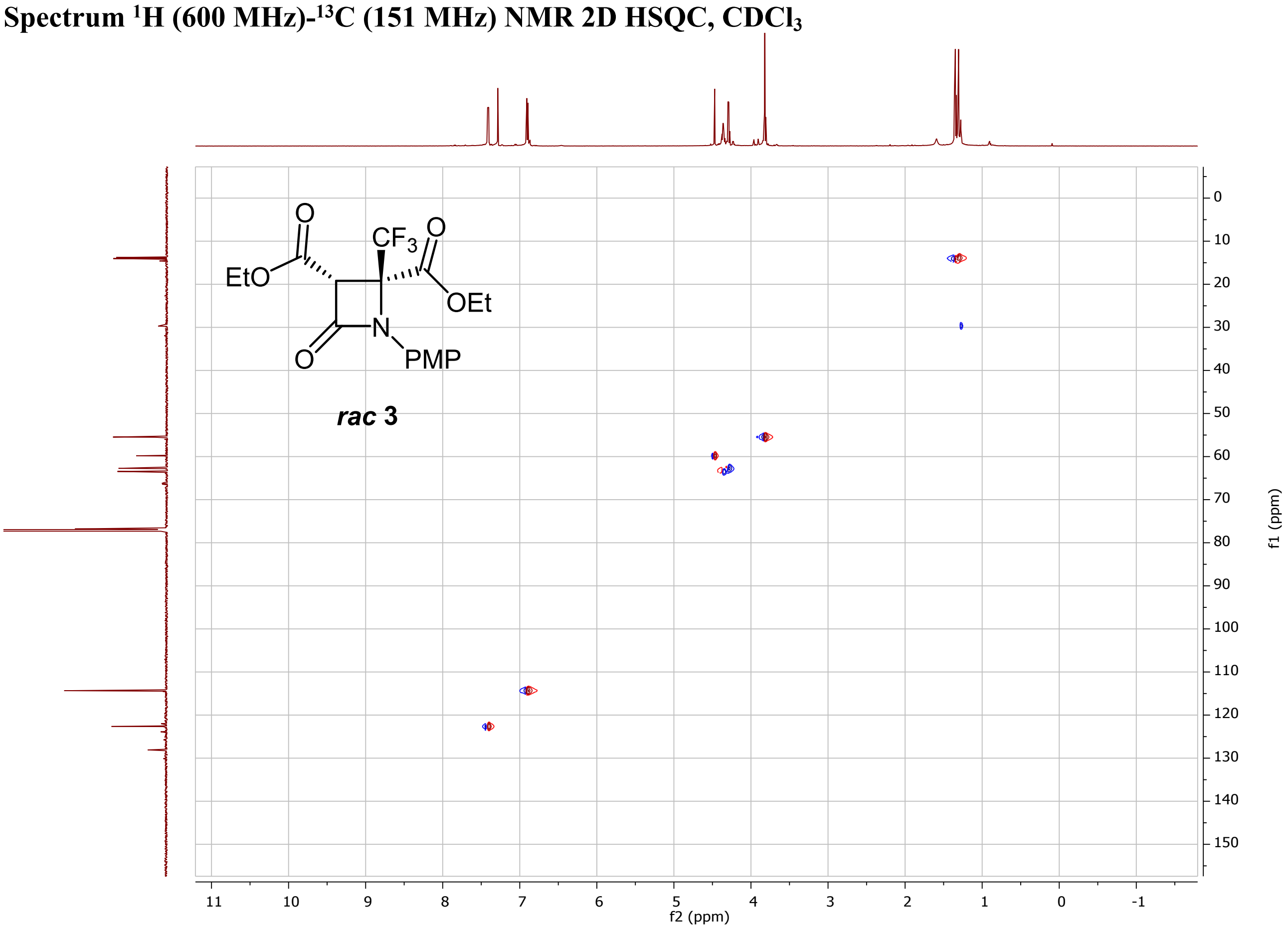

Spectrum ¹H (600 MHz)-¹³C (151 MHz) NMR 2D HMBC, $CDCl_3$

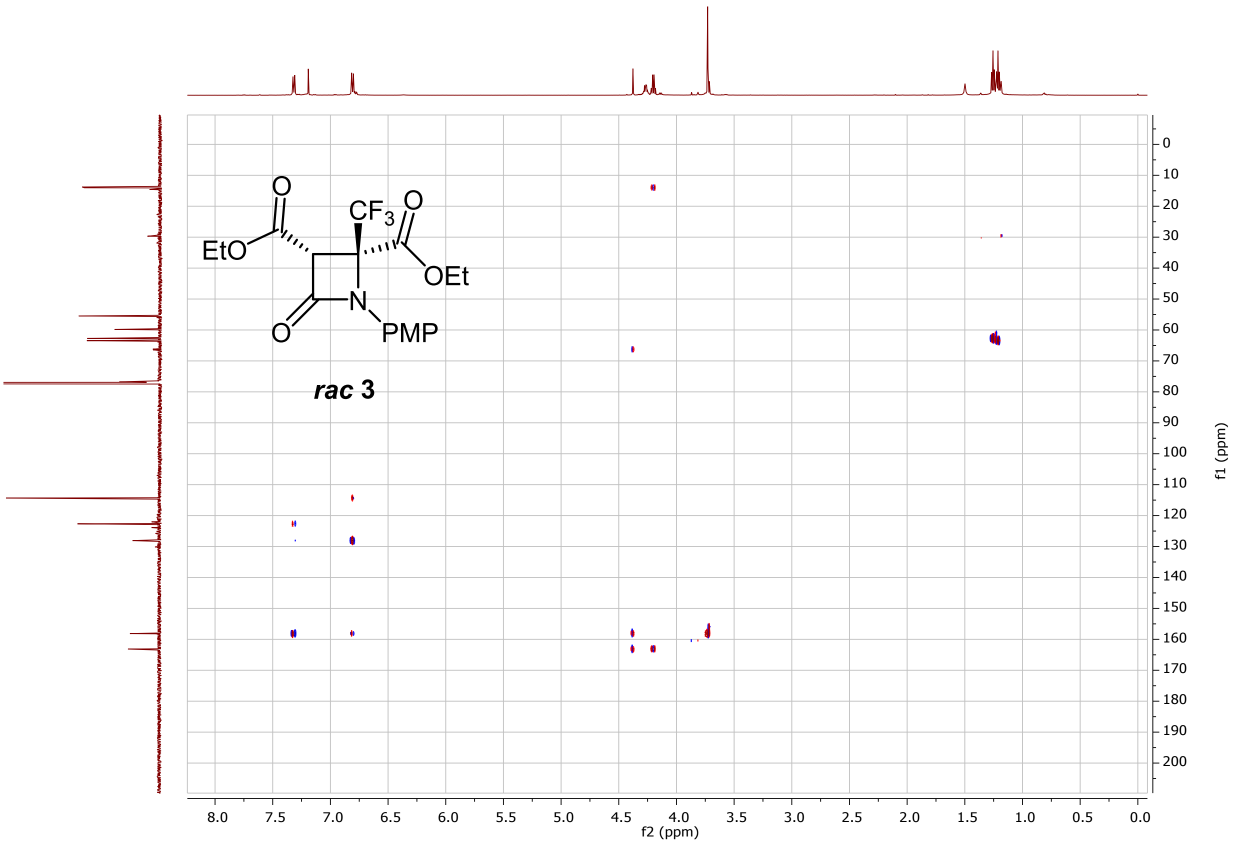

Spectrum ^{1}H (600 MHz)-^{19}F (565 MHz) NMR 2D HOESY, $CDCl_3$

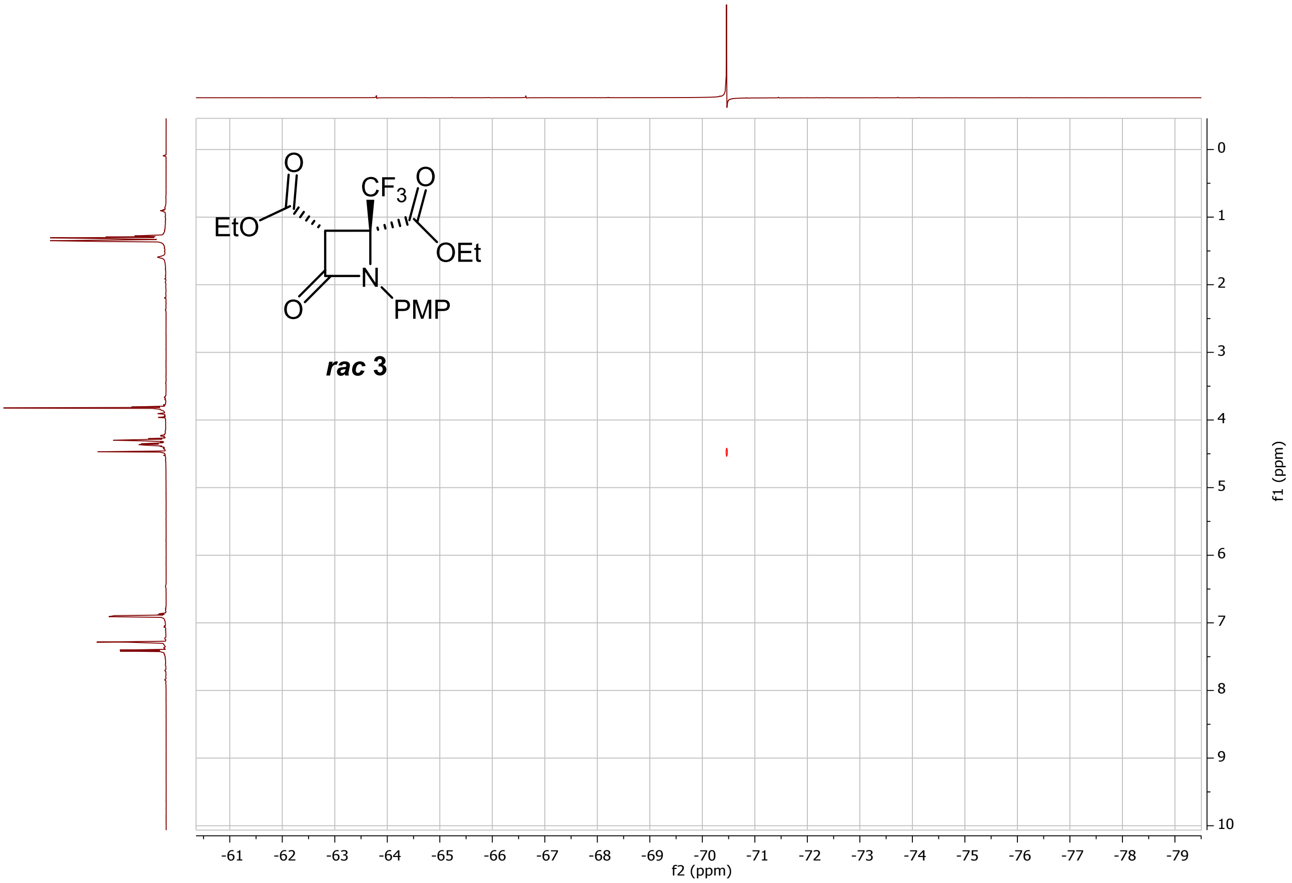

Spectrum ^{1}H (600 MHz)-^{19}F (151 MHz) NMR 2D HOESY (zoom of the signal), $CDCl_3$

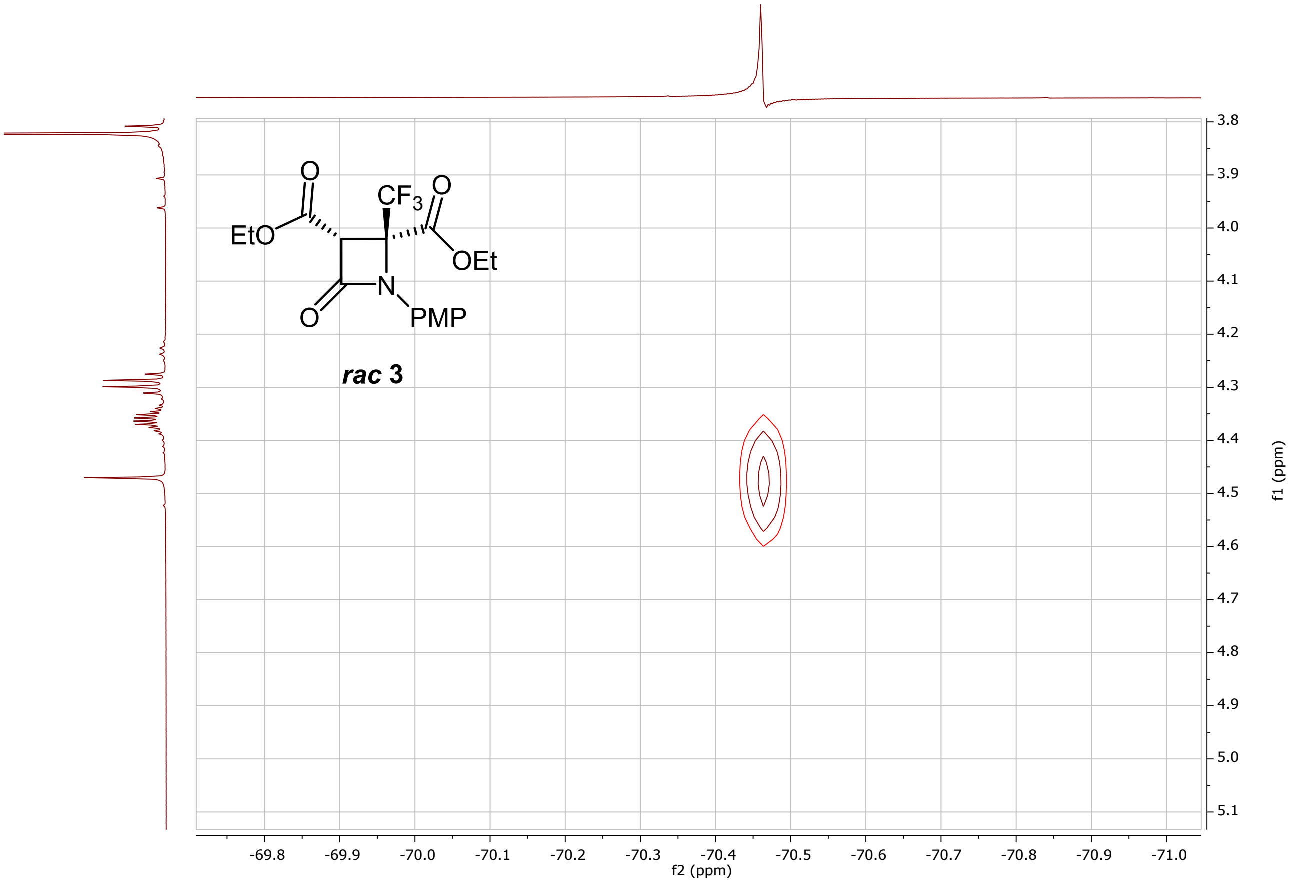

Spectrum ^{1}H NMR (600 MHz, $CDCl_3$)

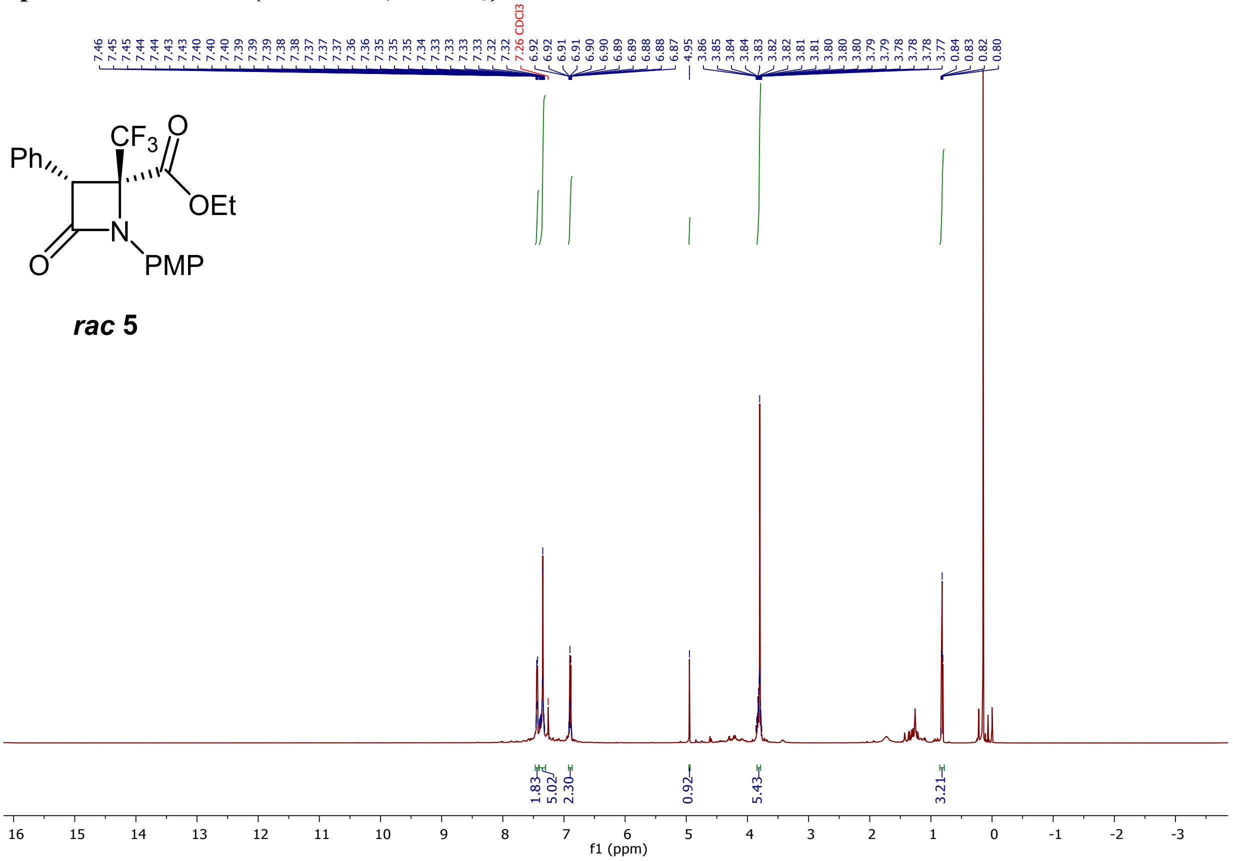

Spectrum ^{13}C NMR (151 MHz, $CDCl_3$)

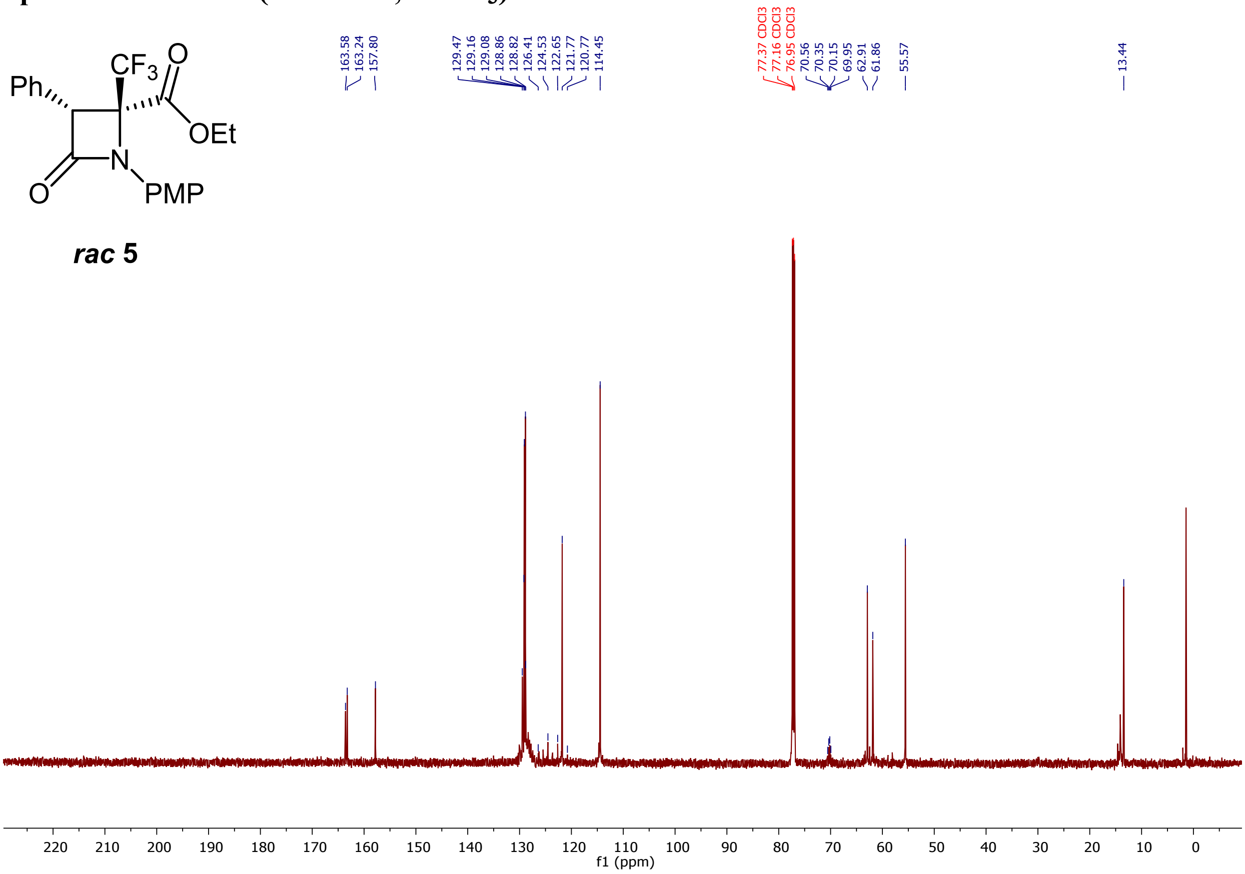

Spectrum ^{19}F NMR (565 MHz, $CDCl_3$)

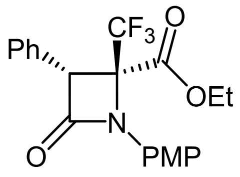

***rac* 5**

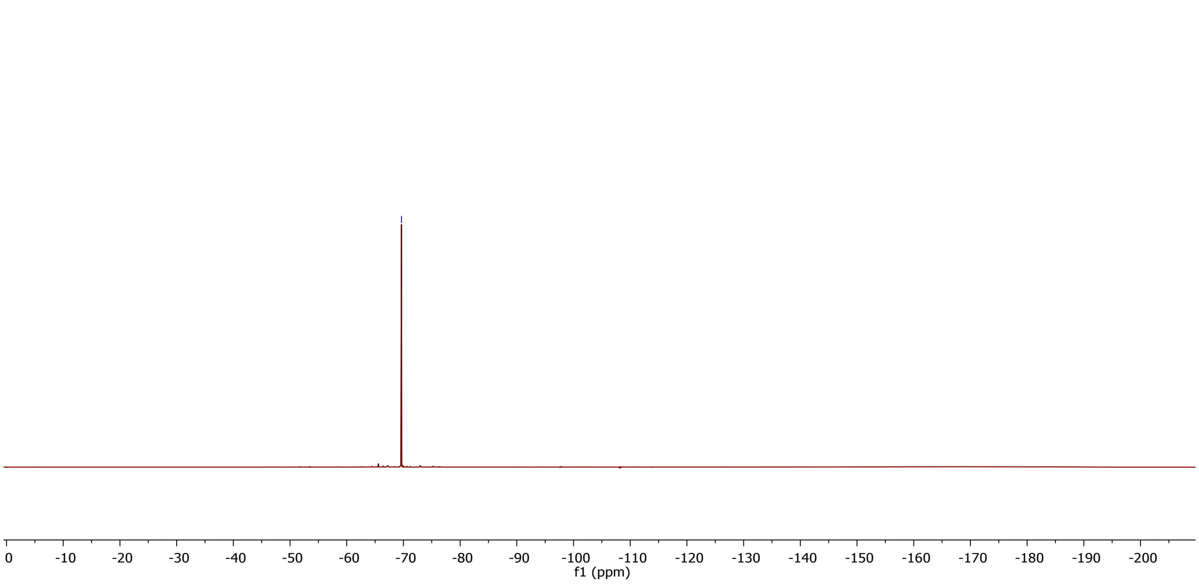

Spectrum ^{1}H (600 MHz)-^{19}F (565 MHz) NMR 1D HOESY, $CDCl_3$

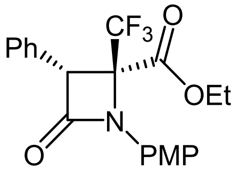

***rac* 5**

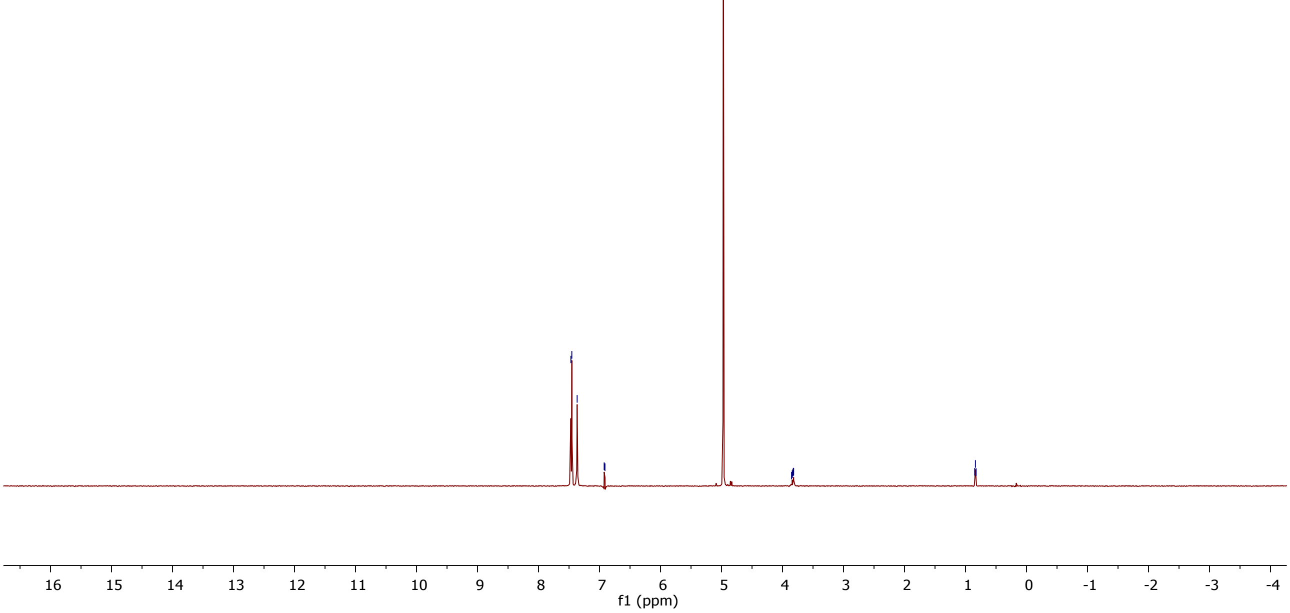

Spectrum ^{1}H (600 MHz)-^{19}F (565 MHz) NMR 2D HOESY, $CDCl_3$

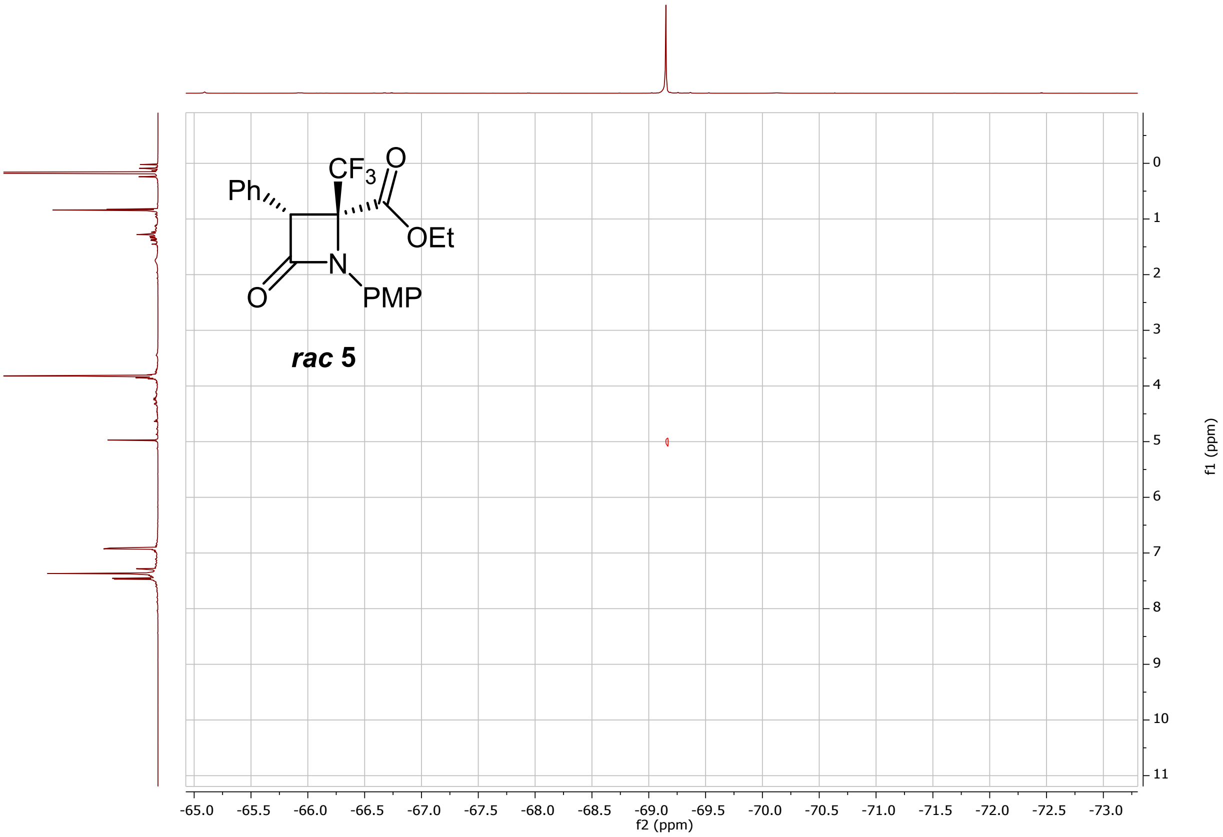

Spectrum ^{1}H (600 MHz)-^{19}F (565 MHz) NMR 2D HOESY (zoom of the signal), $CDCl_3$

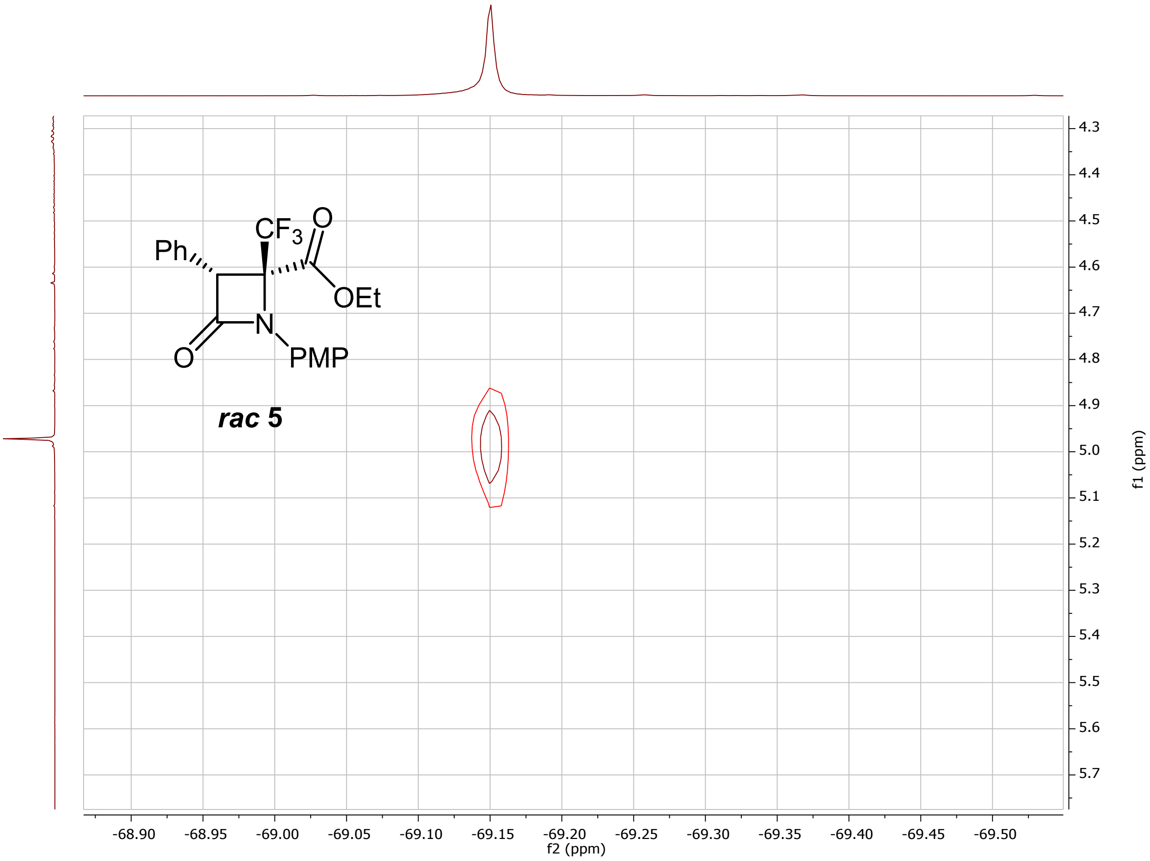

Spectrum ¹H NMR (600 MHz, $CDCl_3$)

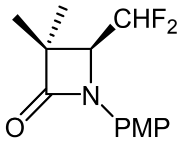

8

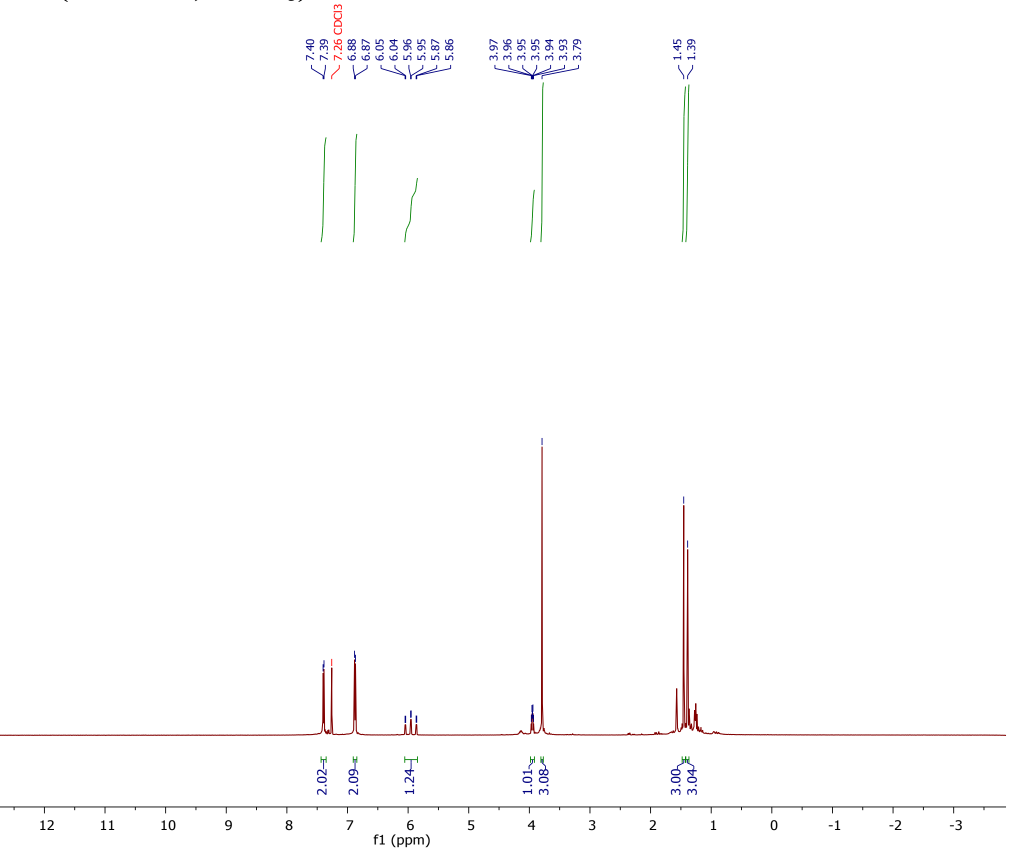

Spectrum ¹³C NMR (151 MHz, $CDCl_3$)

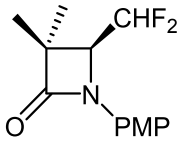

8

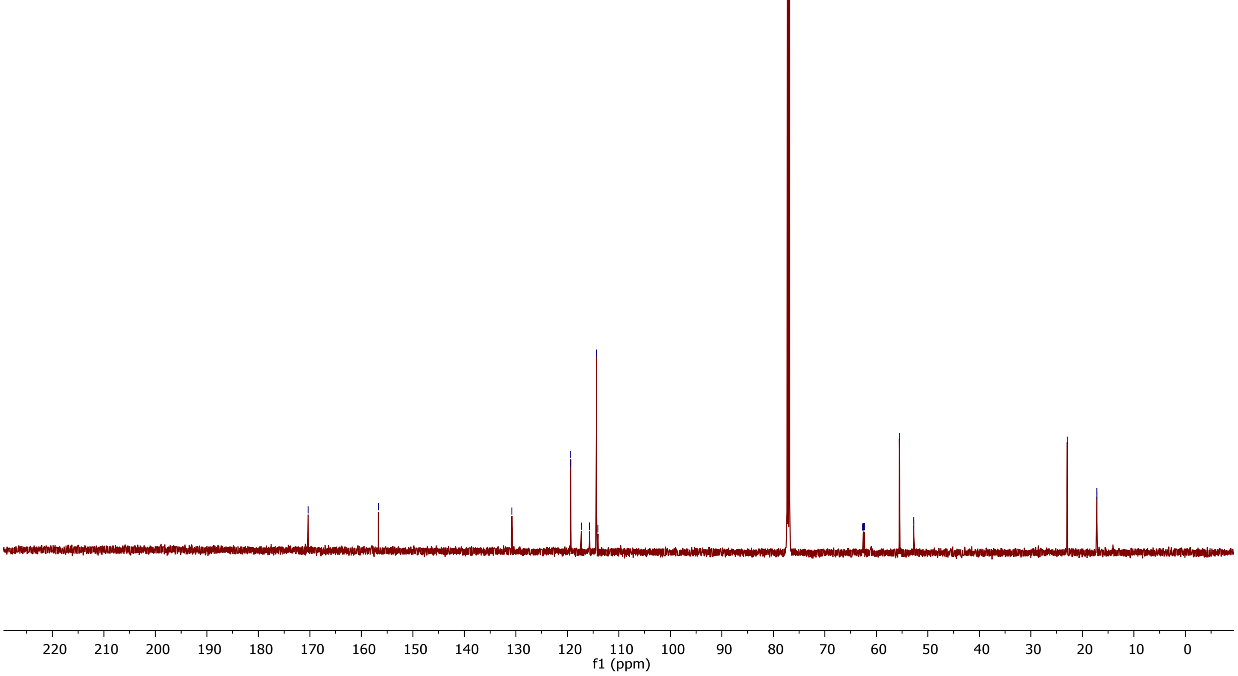

Spectrum ^{19}F NMR (565 MHz, $CDCl_3$)

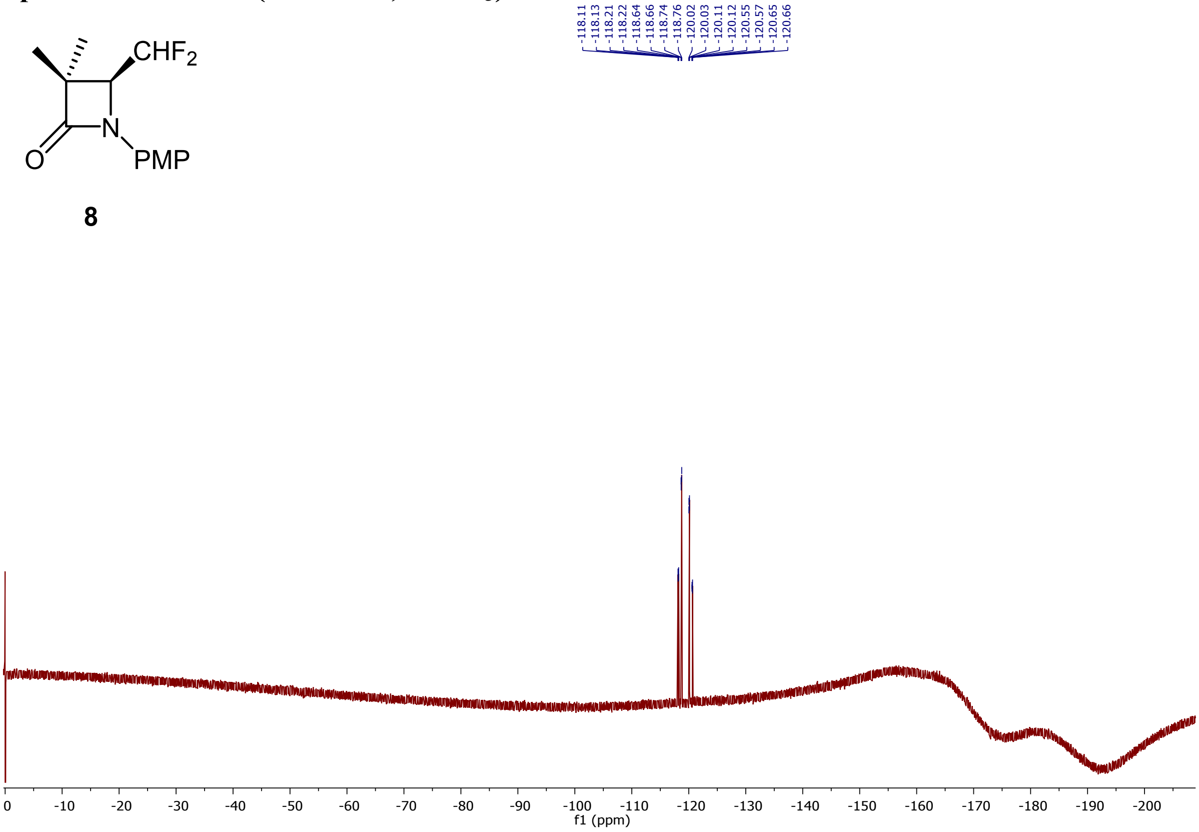

8

Spectrum ^{1}H NMR (600 MHz, $CDCl_3$)

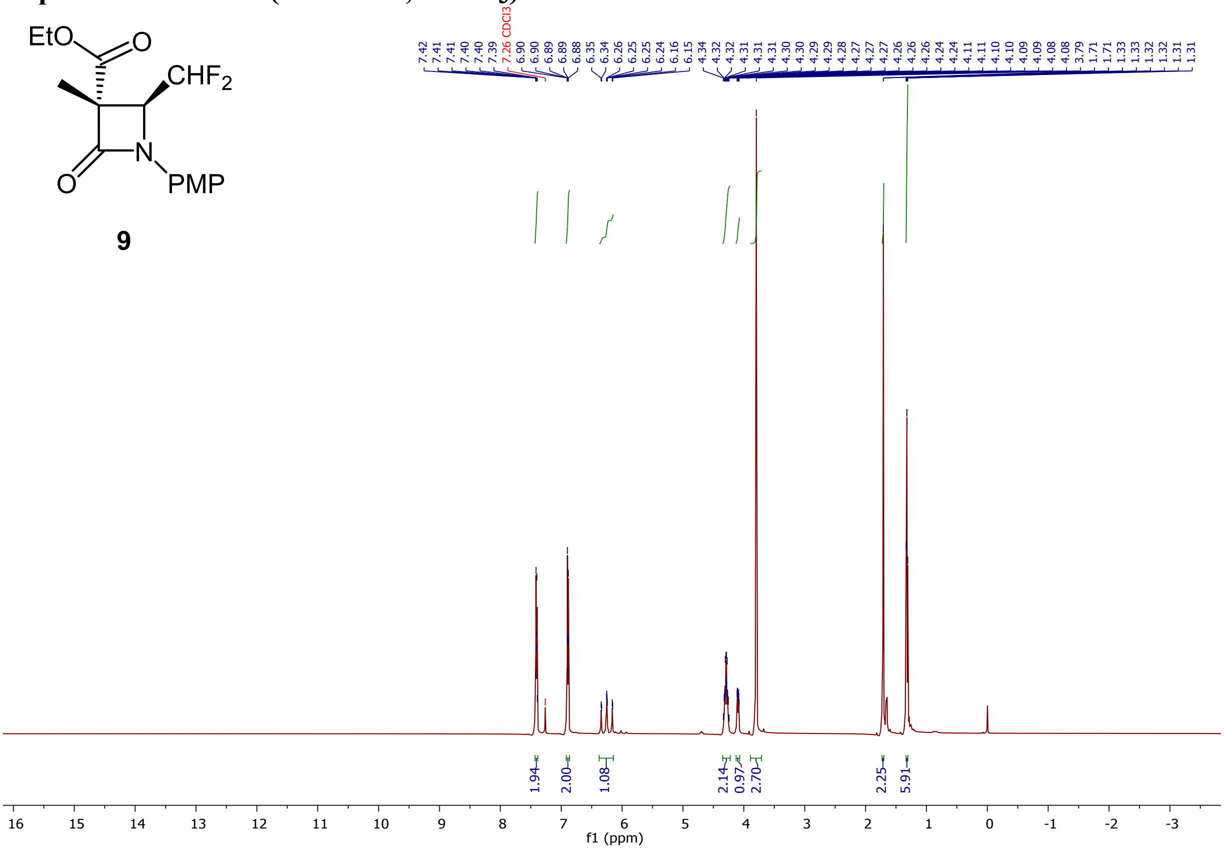

9

Spectrum ^{13}C NMR (151 MHz, $CDCl_3$)

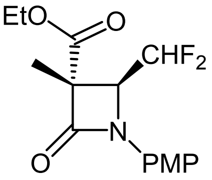

9

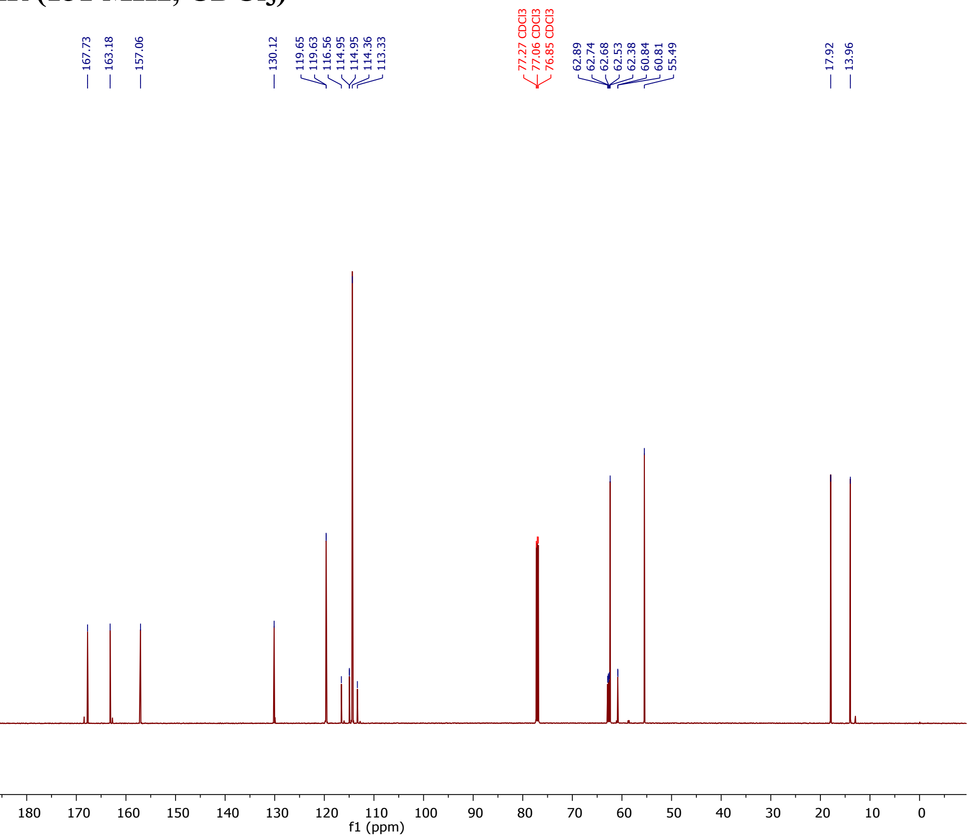

Spectrum ^{19}F NMR (565 MHz, $CDCl_3$)

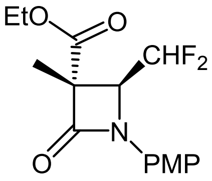

9

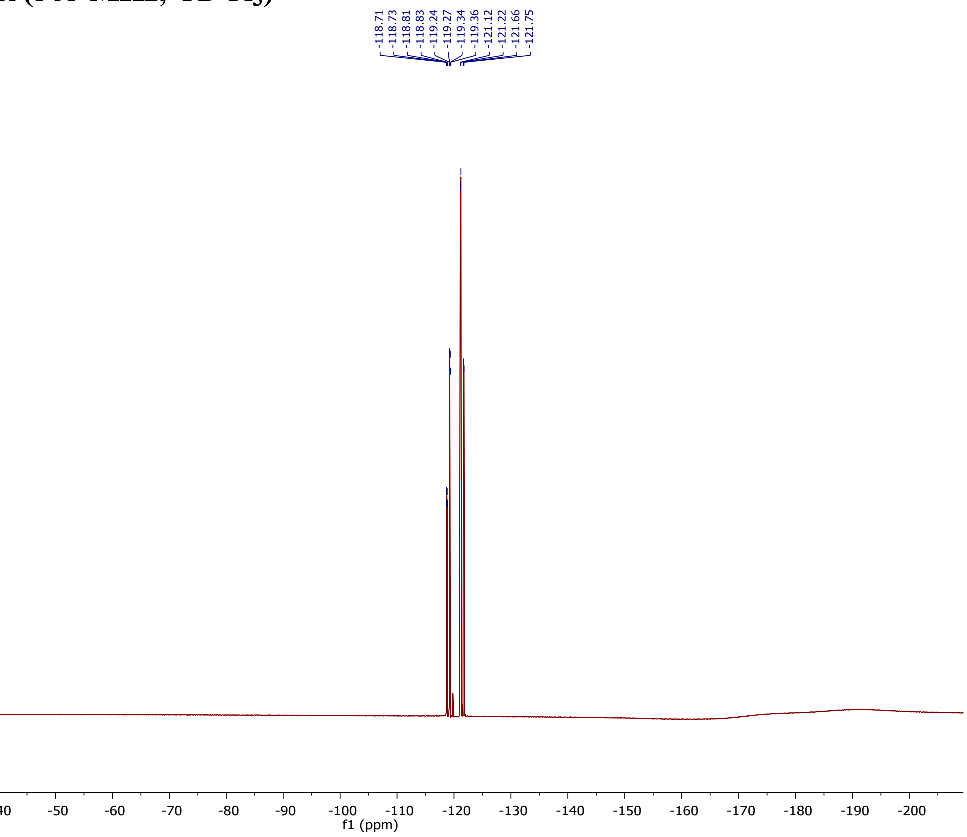

Spectrum ^{1}H NMR (600 MHz, $CDCl_3$)

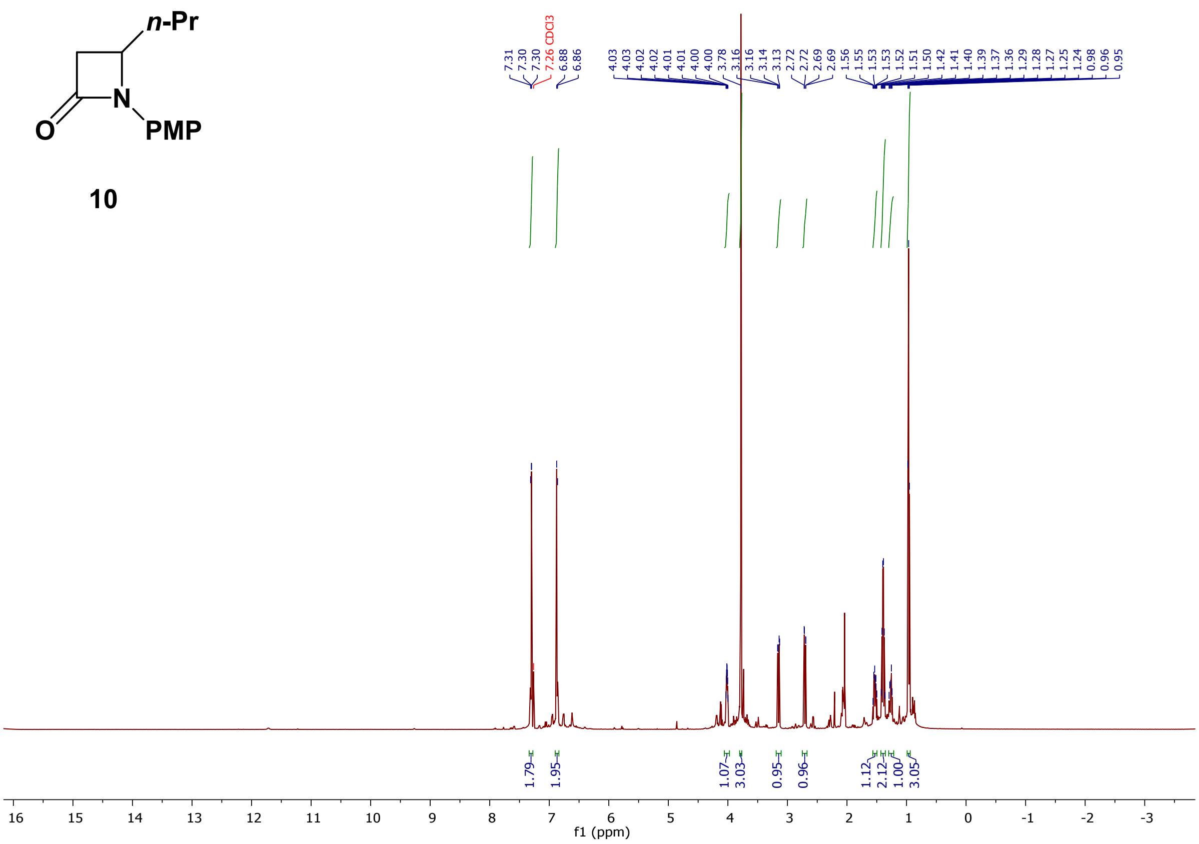

Spectrum ^{13}C NMR (151 MHz, $CDCl_3$)

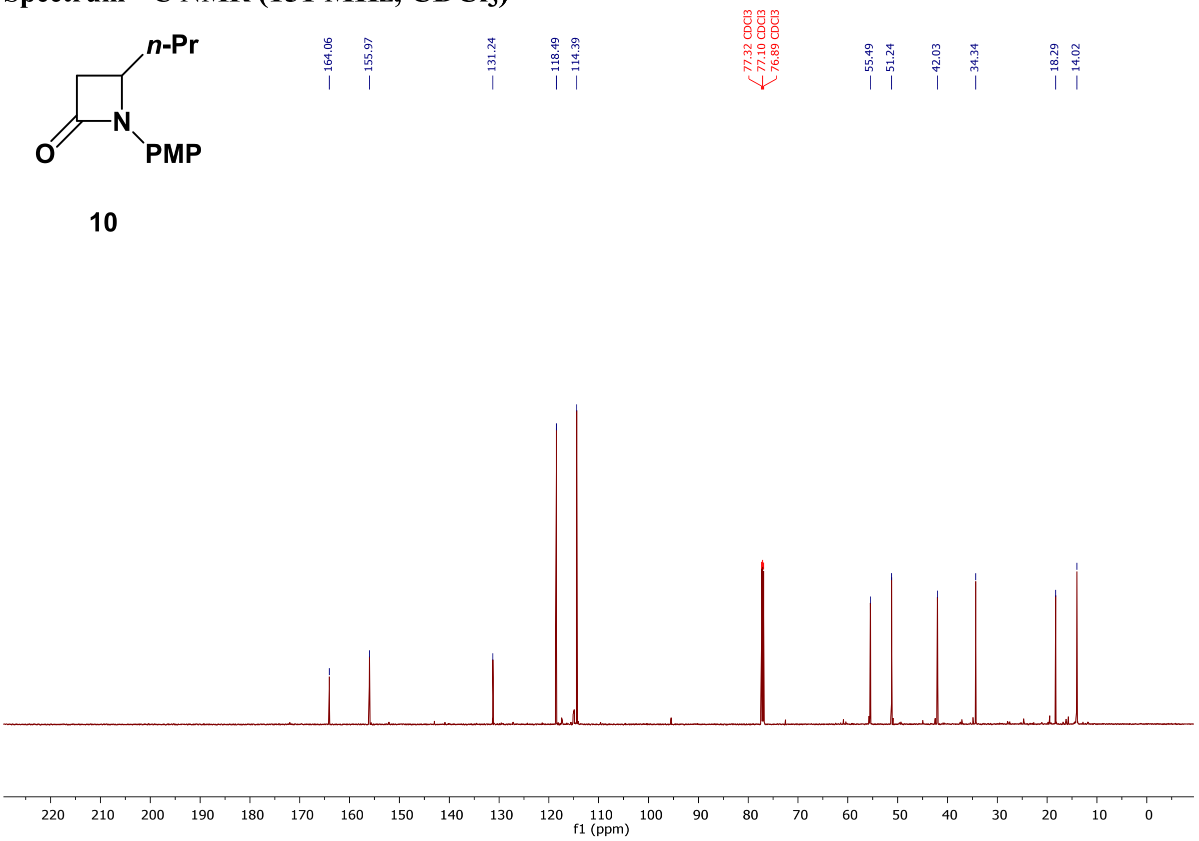

Supplement: Supplementary file 1 — ol3c04094_si_001.pdf [file ol3c04094_si_001.pdf]
